# The genome draft of coconut (*Cocos nucifera*)

**DOI:** 10.1093/gigascience/gix095

**Published:** 2017-10-05

**Authors:** Yong Xiao, Pengwei Xu, Haikuo Fan, Luc Baudouin, Wei Xia, Stéphanie Bocs, Junyang Xu, Qiong Li, Anping Guo, Lixia Zhou, Jing Li, Yi Wu, Zilong Ma, Alix Armero, Auguste Emmanuel Issali, Na Liu, Ming Peng, Yaodong Yang

**Affiliations:** 1 Hainan Key Laboratory of Tropical Oil Crops Biology/Coconut Research Institute, Chinese Academy of Tropical Agricultural Sciences, Av. Wenqing No. 496, Wenchang, Hainan 571339, P. R. China; 2 Institute of Tropical Bioscience and Biotechnology, Chinese Academy of Tropical Agricultural Science, Rd. Xueyuan No. 4, Haikou, Hainan 571101, P. R. China; 3 BGI Genomics, BGI-Shenzhen, Building NO.7, BGI Park, No. 21 Hongan 3rd Street, Yantian District, Shenzhen 518083, China; 4 AGAP, Université de Montpellier, CIRAD, INRA, Montpellier Supagro, F-34398, Montpellier, France; 5 CIRAD, UMR AGAP, F-34398, Montpellier France; 6 Montpellier Supagro, UMR AGAP, F-34398, Montpellier, France; 7 Station Cocotier Marc Delorme, Centre National De Recherche Agronomique (CNRA) 07 B.P. 13, Port Bouet, Côte d’Ivoire

**Keywords:** coconut palm, genome, assembly, annotation

## Abstract

Coconut palm (*Cocos nucifera*,2n = 32), a member of genus *Cocos* and family Arecaceae (Palmaceae), is an important tropical fruit and oil crop. Currently, coconut palm is cultivated in 93 countries, including Central and South America, East and West Africa, Southeast Asia and the Pacific Islands, with a total growth area of more than 12 million hectares [1]. Coconut palm is generally classified into 2 main categories: “Tall” (flowering 8–10 years after planting) and “Dwarf” (flowering 4–6 years after planting), based on morphological characteristics and breeding habits. This Palmae species has a long growth period before reproductive years, which hinders conventional breeding progress. In spite of initial successes, improvements made by conventional breeding have been very slow. In the present study, we obtained *de novo* sequences of the *Cocos nucifera* genome: a major genomic resource that could be used to facilitate molecular breeding in *Cocos nucifera* and accelerate the breeding process in this important crop. A total of 419.67 gigabases (Gb) of raw reads were generated by the Illumina HiSeq 2000 platform using a series of paired-end and mate-pair libraries, covering the predicted *Cocos nucifera* genome length (2.42 Gb, variety “Hainan Tall”) to an estimated ×173.32 read depth. A total scaffold length of 2.20 Gb was generated (N50 = 418 Kb), representing 90.91% of the genome. The coconut genome was predicted to harbor 28 039 protein-coding genes, which is less than in *Phoenix dactylifera* (PDK30: 28 889), *Phoenix dactylifera* (DPV01: 41 660), and *Elaeis guineensis* (EG5: 34 802). BUSCO evaluation demonstrated that the obtained scaffold sequences covered 90.8% of the coconut genome and that the genome annotation was 74.1% complete. Genome annotation results revealed that 72.75% of the coconut genome consisted of transposable elements, of which long-terminal repeat retrotransposons elements (LTRs) accounted for the largest proportion (92.23%). Comparative analysis of the antiporter gene family and ion channel gene families between *C. nucifera* and *Arabidopsis thaliana* indicated that significant gene expansion may have occurred in the coconut involving Na^+^/H^+^ antiporter, carnitine/acylcarnitine translocase, potassium-dependent sodium-calcium exchanger, and potassium channel genes. Despite its agronomic importance, *C. nucifera* is still under-studied. In this report, we present a draft genome of *C. nucifera* and provide genomic information that will facilitate future functional genomics and molecular-assisted breeding in this crop species.

## Data Description

### Background

Coconut palm (*Cocos nucifera*, 2n = 32), the only species in the genus *Cocos* in the family *Arecaceae*, is a tropical oil crop and widely cultivated in tropical regions due to its extensive application in agriculture and industry. Coconut palm is thought to have originated from the Southwest and Western Pacific region (including the Malay Peninsula and Archipelago, New Guinea, and the Bismarck Archipelago). At present, this tropical tree crop is distributed across 93 tropical countries [2], including Central and South America, East and West Africa, Southeast Asia, and the Pacific Islands, and is grown over 12 million hectares of land [1].

**Figure 1:**
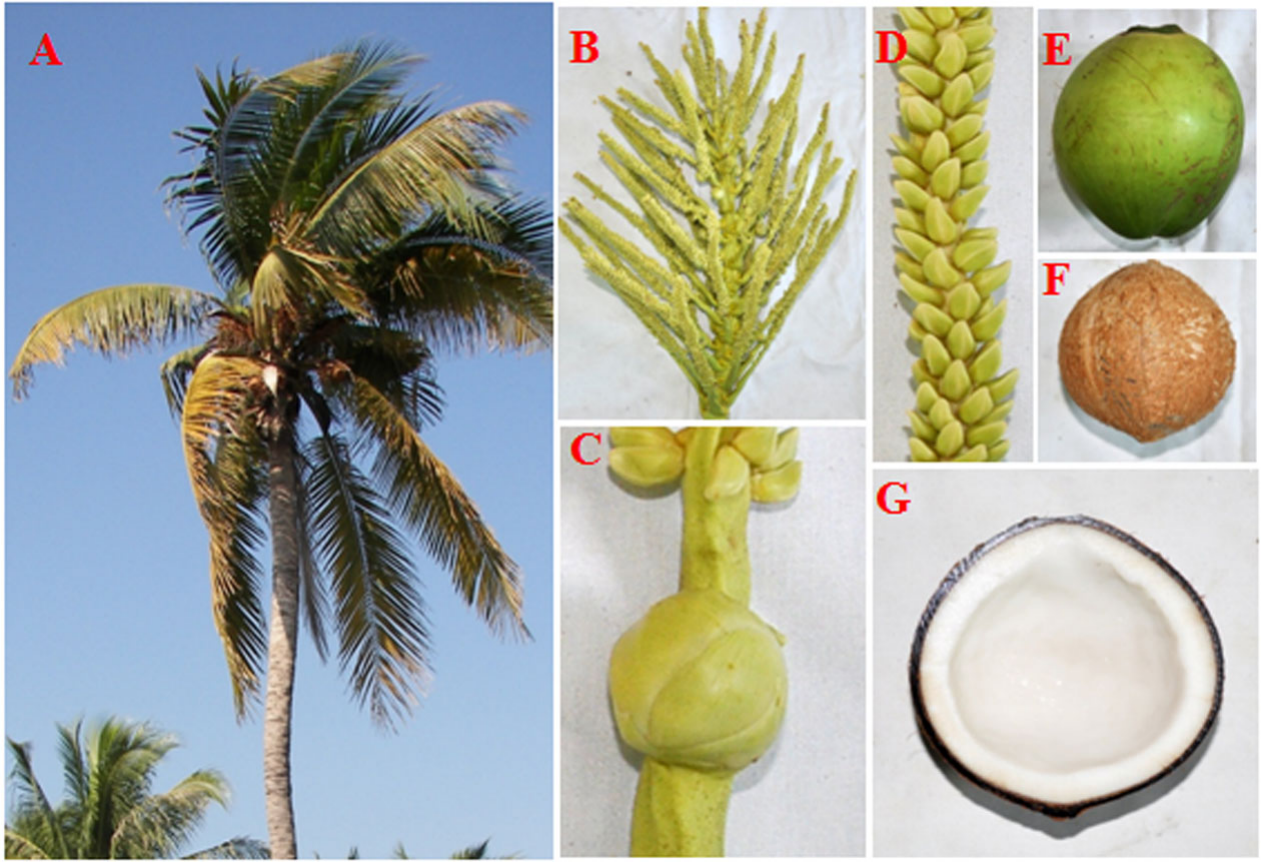
Morphological characteristic of the coconut tree **(A)**, spica **(B)**, female flower **(C)**, male flower **(D)**, coconut nut **(E)**, coconut nut without skin **(F)**, and vertical section of coconut nut **(G)**.

In China, coconut palm grows in the subtropical regions—Hainan and Yunnan provinces—as an economic and ornamental plant. Coconut palm is cultivated over approximately 43 000 hectares in Hainan, with the “Hainan Tall” (HAT) variety covering 36 000 hectares [3]. The HAT coconut needs 8–10 years to enter its reproductive stage and has a height of 20–30 meters, with a medium to large sized nut. The HAT cultivar is highly tolerant to salt and drought stress, but sensitive to temperatures below 10°C. Coconut palm can disseminate through ocean currents: floating nuts sprout and grow naturally upon washing up on beaches. The ability to adapt to a high-salt environment is closely related to this dissemination feature and to these natural growth conditions. The morphological characteristics of the HAT cultivar are shown in Fig. 1. Here, we present the genome sequence of the Hainan Tall coconut and an analysis of the antiporter and ion channel gene families, relevant to salinity tolerance. As draft genome sequences of coconut relatives (e.g., *Elaeis guineensis* [4] and *Phoenix dactylifera* [5, 6]) have previously been reported, we also performed a comparative analysis between the coconut and these relative species for genome assembly and annotation characteristics.

## Data Description

### Sample collection and sequencing strategy

The genomic DNA was extracted from the spear leaf of an individual of the variety “Hainan Tall” coconut (*Cocos nucifera* L. Taxonomy ID: 13 894; 19°33’3”N, 110°47’25”E) from the coconut garden of the Coconut Research Institute (Wenchang, Hainan Province, China) by using the CTAB extraction method [7]. Subsequently, 4 paired-end (PE) libraries with insert sizes of 170 bp, 500 bp, 450 bp, and 800 bp and 5 mate-pair (MP) libraries with insert sizes of 2 Kb, 5 Kb, 10 Kb, 20 Kb, and 40 Kb were constructed using the standard procedure provided by Illumina (San Diego, CA, USA). After library preparation and quality control of the DNA samples, template DNA fragments were hybridized to the surface of the flow cells on an Illumina HiSeq2000 sequencer, amplified to form clusters, and then sequenced by following the standard Illumina manual. Finally, we generated 714.67 Gb of raw reads from all constructed libraries. The raw outputs for each sequenced library are summarized in Table 1. Before assembly, the raw reads were pretreated using the following stringent filtering processes via SOAPfilter (v2.2) [8] software: (1) removed reads with 25% low-quality bases (quality scores ≤7); (2) removed reads with N bases more than 1%; (3) discarded reads with adapter contamination and/or polymerase chain reaction duplicates; (4) removed reads with undersized insert sizes. Finally, 419.08 Gb (estimated 173.17 × read depth) of high-quality sequences were obtained for genome assembly.

**Table 1:** Data outputs produced by sequencing different insert size libraries.

Library type	Lane	Reads length, bp	Insert size, bp	Raw data, Gb	Clean data, Gb
PE101	3	100	170	128.75 (53.20)	111.32 (46)
PE251	2	250	450	73.86 (30.52)	56.42 (23.31)
PE101	2	100	500	64 (26.45)	65.11 (26.90)
PE101	2	100	800	78.16 (32.30)	64.90 (26.82)
MP50	3	49	2000	128.6 (53.14)	60.70 (25.08)
MP50	2	49	5000	71.75 (29.65)	18.62 (7.69)
MP50	2	49	10 000	74.65 (30.85)	18.53 (7.66)
MP50	2	49	20 000	70.7 (29.21)	19.35 (7.99)
MP50	1	49	40 000	24.2 (10.08)	4.13 (1.71)
Total	19			714.67 (295.32)	419.08 (173.17)

The sequencing depth is shown in parentheses, calculated based on a genome size of 2.42 G. Clean data were obtained by filtering raw data with low-quality and duplicate reads.

### *De novo* assembly of short reads of *Cocos nucifera*

**Figure 2:**
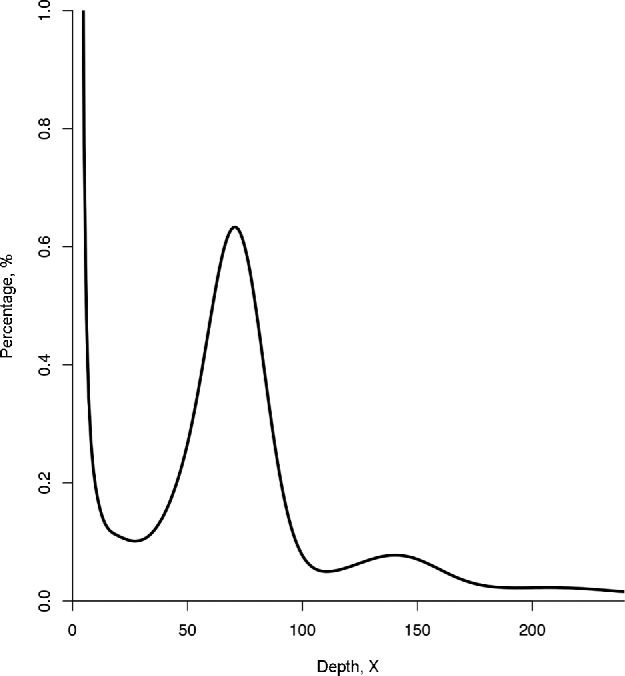
K-mer analysis of the coconut genome.

We used 209.38 Gb of clean reads of the short-insert libraries (excluding the 450-bp library) to estimate the coconut genome size by k-mer frequency distribution analysis [8]. The genome size (G) of *Cocos nucifera* could be estimated by the following formula:

\begin{equation*}{\rm G} = {\rm N} \times ({\rm L} - {\rm K} + 1)/{\rm K}\_{\rm depth},\end{equation*}

where N represents the total of number of reads, L represents the read length, K represents the k-mer value used in the analysis, and K_depth refers to the main peak in the k-mer distribution curve. In our calculations, N was 2 049 520 223, L was 100, and K_depth was 71 for K = 17. As a result, the *Cocos nucifera* genome was estimated to be 2.42 gigabases (Gb). K-mer size distribution analysis (Fig. 2) indicated that *Cocos nucifera* was a diploid species with low heterozygosity and a high proportion of repetitive sequences.

We then assembled the *Cocos nucifera* genome using the software SOAPdenovo2 (SOAPdenovo2, RRID:SCR_014986) in 3 steps: contig construction, scaffold construction, and gap filling. In the contig construction step, the SOAPdenovo2 was run with the parameters “pregraph -K 63 -R -d 1” to construct de Bruijn graphs from paired-end libraries with insert sizes ranging from 170 to 800 bp. The k-mers from the de Bruijn graphs were then used to form contiguous sequences (contigs) with the parameters “contig -R” by clipping tips, merging bubbles, and removing low-coverage links. In the scaffold construction step, the orders of the contigs were determined by using paired-end and mate-pair information with parameters “map -k 43” and “scaff -F -u”. In more detail, SOAPdenovo2 maps the reads from paired-end and mate-pair libraries to contigs based on a hash table (keys are unique k-mers on contigs; values are positions). In such cases, 2 contigs are considered to be linked if the bridging of the contigs is supported by 5 paired-end read pairs or 3 mate-pair read pairs. In the gap filling step, gaps within scaffolds were filled by utilizing KGF [8] v1.06 and GapCloser v1.12-r6 (GapCloser, RRID:SCR_015026) [8] with paired-end libraries (having an insert size from 170 to 800 bp in cases, where 1 end could be mapped to 1 contig and the other end extended into a gap). To optimize the assembled sequence, Rabbit (a Poisson-based k-mer model software [9]) was used to remove the redundant sequences. A final length of 2.20 Gb for the scaffolds was obtained and used for further analysis, accounting for 90.91% of the predicted genome size and larger than the African oil palm and date palm genomes (Table 2). Meanwhile, the N50 of the obtained contigs was 72.64 Kb and 418.06 Kb for the scaffolds, which have excluding scaffolds of less than 100 bp. The comparison of N50 values for the assembled coconut genome and for the 4 previously published palm genomes *Elaeis guineensis* [4], *Elaeis oleifera* [4], *Phoenix dactylifera* (PDK30) [5], and *Phoenix dactylifera* (DPV01) [6] is listed in Table 2.

**Table 2:** Comparison analysis of genome sizes, assembly, and annotation of 4 palmae species, including coconut, *Phoenix dacylifera* (PDK30 and DPV01, 2 different versions), *Elaeis guineensis* (EG), and *Elaeis oleifera* (EO).

	Sequencing	Sequence	Estimated	Assembly	Contig	Scaffold	Gene	TEs,
Species	technology	coverage	size, Gb	size, Gb	N50, Kb	N50, Kb	number	%
*Phoenix dactylifera* (PDK30)	Illumina GAIIx	×53.4	0.66	0.38	6.44	30.48	28 889	23.6
*Phoenix dactylifera* (DPV01)	454, SOLiD, ABI3730	×139	0.67	0.56	10.81	334.08	41 660	38.87
*Elaeis guineensis* (African oil palm)	454	×16	1.8	1.54	9.37	1045.41	34 802	43.24
*Elaeis oleifera* (American oil palm)	454	×16	1.8	1.40	8.45	333.11	–	–
*Cocos nucifera* (Hai nan Tall)	Illumina HiSeq	×173	2.42	2.20	72.64	418.07	28 039	72.75

Coconut: *Cocos nucifera* (Hainan Tall); PDK30: *Phoenix dactylifera* (PDK30); DPV01: *Phoenix dactylifera* (DPV01); EG: *Elaeis guineensis* (Africanoil palm E5 build); EO: *Elaeis oleifera* (American oil palm, O8-build). TE results were obtained using the same pipeline as for the coconut genome

### Genome evaluation

The 57 304 unigenes (transcript obtained from 3 different tissues, spear leaves, young leaves, and fruit flesh), as previously reported by Fan et al. [10], were aligned to the assembled genome of *Cocos nucifera* using BLAT (BLAT, RRID:SCR_011919) [11] with default parameters. The alignment results indicated that the assembled genome of *Cocos nucifera* covered 96.78% of the expressed unigenes, suggesting that a high level of coverage has been reached for the assembled genome (Table 3).

**Table 3:** The gene coverage of *Cocos nucifera* based on transcriptome data.

Data set	Number	Total length, bp	Base coverage by assembly	Sequence coverage by assembly, %
All	57 304	43 090 665	96.78	99.57
>200 bp	57 304	43 090 665	96.78	99.57
>500 bp	25 713	33 470 388	96.36	99.85
>1000 bp	13 796	25 004 919	95.99	99.94

We also evaluated the level of genome completeness for the assembled sequences by using BUSCO v2.0 (BUSCO, RRID:SCR_015008) [12], which quantitatively assesses genome completeness using evolutionarily informed expectations of gene content from near-universal single-copy orthologs selected from OrthoDB v9 (OrthoDB, RRID:SCR_011980; plant set) [13]. BUSCO analysis showed that 90.8% and 3.4% of the 1440 expected plant genes were identified as complete and fragmented genes, respectively, while 5.8% of genes were considered to be missing from the assembled coconut genome sequence. The comparative results of the BUSCO estimation in the coconut and in the 4 other palm genome sequences indicates that the smallest fraction of missing genes as predicted by BUSCO was found in the coconut genome assembly (Table 4).

**Table 4:** The comparative analysis of assembly results of 5 palm species with BUSCO software, including coconut, *Phoenix dacylifera* (PDK30 and DPV01, 2 varieties), *Elaeis guineensis* (EG), and *Elaeis oleifera* (EO).

	Coconut		PDK30		DPV01		EG		EO	
BUSCOs	No.	P, %	No.	P, %	No.	P, %	No.	P, %	No.	P, %
Total	1440		1440		1440		1440		1440	
Complete single-copy	1192	82.8	1042	72.4	1160	80.6	1100	76.4	1004	69.7
Complete duplicated	115	8.0	81	5.6	134	9.3	116	8.1	63	4.4
Fragment	49	3.4	98	6.8	42	2.9	60	4.2	84	5.8
Missing	84	5.8	219	15.2	104	7.2	164	11.3	289	20.1

Coconut: *Cocos nucifera* (the Hainan Tall); PDK30: *Phoenix dactylifera* (PDK30); DPV01: *Phoenix dactylifera* (DPV01); EG: *Elaeis guineensis* (African oil palm E5 build); EO: *Elaeis oleifera* (American oil palm, O8-build).

### Repeat annotation

We combined homology-based annotation and a *de novo* method to identify transposable elements (TEs) and the tandem repeats in the *Cocos nucifera* genome. In the homology-based annotation step, TEs were identified by searching against the Repbase library (v20.04) [14] with RepeatMasker (v4.0.5; RepeatMasker, RRID:SCR_012954) [15] and RepeatProteinMasker (v4.0.5) [15]. In the *de novo* step, *de novo* libraries were constructed based on the genome sequences using the *de novo* prediction program RepeatModeler (RepeatModeler, RRID:SCR_015027) and LTR_FINDER (LTR_FINDER, RRID:SCR_015247) [16] by removing contaminant and multi-copy genes. Subsequently, novel transposable elements were identified and classified using RepeatMasker. Tandem repeat sequences were identified by Tandem Repeat Finder (TRF) software [17] with the following parameters “Match = 2, Mismatch = 7, Delta = 7, PM = 80, PI = 10, Minscore = 50 and MaxPerid = 2000”. The total length of the tandem repeat sequences predicted by the software was 151 229 585 bp, comprising 6.86% of the coconut genome. Finally, 1.6 Gb of non-redundant repetitive elements were identified, accounting for 74.48% of the coconut genome. Transposable elements took up 72.75% of the total 1.6 Gb of repetitive elements, with the long-terminal repeat retrotransposon (LTR) class accounting for 92.23% of all TEs and 67.1% of the coconut genome (Table 5).

**Table 5:** Classification of predicted transposable elements in the coconut genome.

	Repabse TEs	Protein TEs	*De novo* TEs	Combined TEs	
				
	Length	Length	Length	Length	Percentage
DNA	20 936 158	24 655 089	35 131 002	58 119 982	2.64
LINE	4 251 185	9 631 472	7 610 172	19 197 064	0.87
SINE	85 717	0.00	186 364	270 055	0.012
LTR	361 968 154	512 700 933	1 419 281 798	1 478 182 089	67.10
Other	8145	0.00	0.00	8145	0.0004
Unknown	0.00	12 360	139 084 335	139 096 695	6.31
Total	385 037 442	546 965 774	1 552 582 881	1 602 630 396	72.75

Note: Repabse TEs means RepeatMask against Repbase; Protein TEs means RepeatProteinMask result against Repbase protein; *De novo* TEs means RepeatMask against the *de novo* library; Combined TEs: the combined results of these 3 steps.

### Gene prediction

We combined 3 strategies to predict genes in the *Cocos nucifera* genome: homology-based, *de novo*, and transcript alignment. For homology-based annotation, the protein sequences of *Arabidopsis thaliana* [18], *Oryza sativa* [19], *Sorghum bicolor* [20], *Zea mays* [21], *Elaeis guineensis*, and *Phoenix dactylifera* (DPV01) were downloaded from each corresponding source (see “Availability of data sources”). The coconut genome was aligned against these downloaded databases using TBLASTN [22] with parameter “-e 1e-5 -F -m 8” and BLAST results were processed by solar (v0.9) with parameter “-aprot 2 genome2 -z” to determine the candidate gene loci. Next, we extracted the genomic sequences of candidate gene loci, along with 1 kb of flanking sequences, and applied GeneWise 2.2.0 (GeneWise, RRID:SCR_015054) [23] to define the intron—exon boundaries. The genes with pre-stop codon or frame-shifts were excluded from further analysis.

For *de novo* prediction, we randomly selected 1000 full-length genes (GeneWise score equal to 100, intact structure: start codon, stop codon, perfect intron-exon boundary) from gene models predicted by homology-based methods to train the model parameters for AUGUSTUS 2.5 (Augustus: Gene Prediction, RRID:SCR_008417) [24]. Two software programs, AUGUSTUS 2.5 and GENSCAN (GENSCAN, RRID:SCR_012902) 1.0 [25], were used to do *de novo* prediction on the repeat-masked genome of *Cocos nucifera*. Genes with incomplete structure or a protein coding length of less than 150 bp were filtered out.

Subsequently, genes from both homology-based and *de novo* methods were combined to obtain non-redundant gene sets by using GLEAN [26] with the following parameters: minimum coding sequence length of 150 bp and maximum intron length of 50 kb. Genes were filtered with the same thresholds as were used for homology-based annotation.

For transcriptome-based prediction, RNA-seq data (SRR606452), as previously reported by Fan et al. [10], were mapped onto the coconut genome to identify the splice junctions using the software TopHat v2.1.1 (TopHat, RRID:SCR_013035) [27]. The software Cufflinks v2.2.1 (Cufflinks, RRID:SCR_014597) [28] was then used to assemble transcripts with the aligned reads. The coding potential of these transcripts was identified using a fifth-order Hidden Markov Model, which was estimated with the same gene sets used in AUGUSTUS training by train GlimmerHMM, an application in the GlimmerHMM package (GlimmerHMM, RRID:SCR_002654) [29]. The transcripts with intact open reading frames (ORFs) were extracted, and the longest transcript was retrieved as a representative of a gene from multiple transcripts on the same locus.

**Figure 3:**
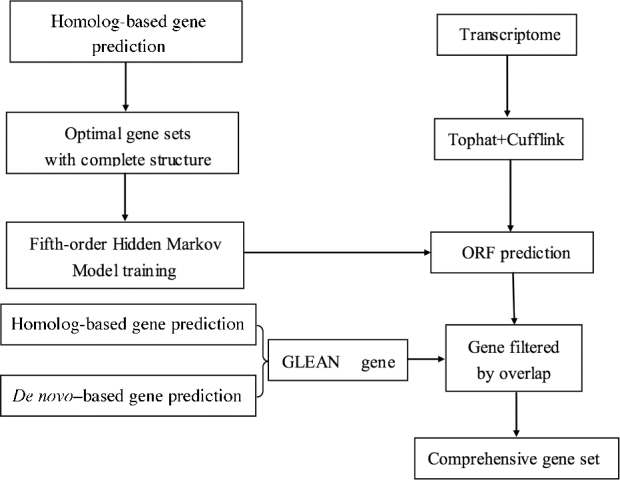
The protocol for integrating GLEAN and transcriptome data.

Finally, we merged the GLEAN and the transcriptome result to form a comprehensive gene set using an in-house annotation pipeline with the following steps: first, all-to-all BLASTP analysis of protein sequences was performed between GLEAN results and transcript assemblies, with an E-value cutoff of 1e-10. These transcript assemblies were added to the GLEAN result to form untranslated region (UTRs) or alternative splicing products, depending on whether the coverage and identity of the alignment results reached 0.9 or not. If the transcript assemblies had no BLAST hit with the GLEAN results, these transcript assemblies were added to the final gene set as a novel gene. The protocol for integrating GLEAN and transcriptome data is shown in Fig. 3.

### Gene evaluation

The annotation processes identified 28 039 protein-coding genes (Table 2), which is less than the predicted gene numbers of *Phoenix dactylifera* (PDK30, 28 889), *Phoenix dactylifera* (DPV01, 41 660), and *Elaeis guineensis* (34 802). Meanwhile, the BUSCO evaluation showed that 74.1% and 11.2% of 1440 expected plant genes were identified as complete and fragmented, with 14.7% of genes considered missing in the gene sets. The BUSCO results showed that our gene prediction was more complete than that of *Phoenix dactylifera* (PDK30) and *Elaeis guineensis*, but less complete than that of *Phoenix dactylifera* (DPV01) (Table 6).

**Table 6:** The comparative analysis of gene prediction results of 4 palm species with BUSCO software.

	Coconut		PDK30		DPV01		EG	
BUSCOs	No.	P, %	No.	P, %	No.	P, %	No.	P, %
Total	1440		1440		1440		1440	
Complete single-copy	965	74.1	748	51.9	1195	83.0	555	38.5
Complete duplicated	102	7.1	81	5.6	159	11.0	53	3.7
Fragment	162	11.2	255	17.7	44	3.1	270	18.8
Missing	211	14.7	356	24.8	42	2.9	562	39.0

Note: Coconut: *Cocos nucifera* (the Hainan Tall); PDK30: *Phoenix dactylifera* (PDK30); DPV01: *Phoenix dactylifera* (DPV01); EG: *Elaeis guineensis* (African oil palm E5 build). The gene of *Elaeis oleifera* (American oil palm, O8-build) was missing, not attained from the public database.

### Gene function

Gene function annotation was done based on sequence similarity and domains conservation. First, the coconut protein coding genes were aligned against the KEGG (KEGG, RRID:SCR_012773) protein database [30], SwissProt, and TrEMBL [31], using BLASTP at a cut-off E-value threshold of 10^−5^. Subsequently, the best match from the alignment was used to represent the gene function. We obtained 18 445 KEGG, 18 867 Swissprot, and 24 882 Tremble annotated genes. Second, InterProScan (InterProScan, RRID:SCR_005829) 5.11–51.0 software [32] was employed to identify the motif and domain based on the public databases Pfam (Pfam, RRID:SCR_004726) [33], PRINTS (PRINTS, RRID:SCR_003412) [34], ProDom (ProDom, RRID:SCR_006969) [35], SMART (SMART, RRID:SCR_005026) [36], PANTHER (PANTHER, RRID:SCR_004869) [37], TIGRFAM (JCVI TIGRFAMS, RRID:SCR_005493) [38], and SUPERFAMILY (SUPERFAMILY, RRID:SCR_007952) [39]. The gene function annotation demonstrated that 21 087 of the coconut proteins had conserved motifs, and 1622 gene ontology (GO) terms were assigned to 15 705 coconut proteins from the corresponding InterPro (InterPro, RRID:SCR_006695) entry [40]. In total, approximately 89.41% of these genes were functionally annotated using the above methods.

### Gene family construction

**Figure 4:**
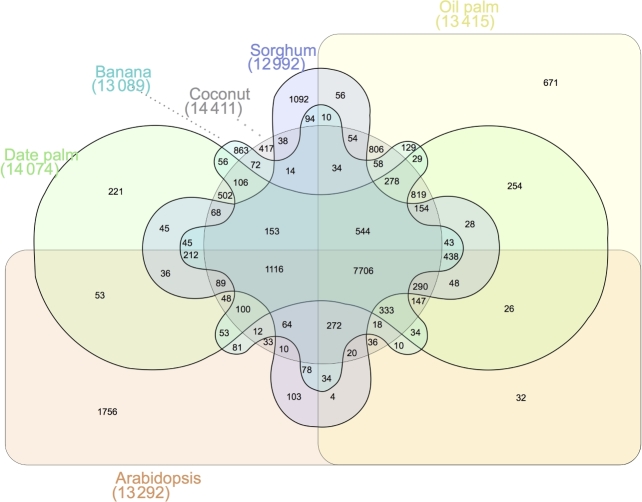
Groups of orthologues shared among the angiosperms *Cocos nucifera* (Coconut), *Elaeis guineensis* (Oil palm), *Phoenix dactylifera* (Date palm), *Sorghum bicolor* (Sorghum), *Musa acuminate* (Banana) and *Arabidopsis thaliana* (Arabidopsis). Venn diagram generated by http://www.interactivenn.net/.

Protein sequences of 13 angiosperms, including *Elaeis guineensis*, *Phoenix dactylifera* (DPV01), *Sorghum bicolor*, *Prunus persica*, *Solanum tuberosum*, *Glycine max*, *Arabidopsis thaliana*, *Theobroma cacao*, *Vitis vinifera*, *Musa acuminata*, *Carica papaya*, *Populus trichocarpa*, and *Amborella trichopoda*, were downloaded from each corresponding ftp site (see “Availability of data sources”). For genes with alternative splicing variants, the longest transcripts were selected to represent the gene. The gene numbers of *Elaeis guineensis* and *Phoenix dactylifera* (DPV01) were greatly different from the research paper published in 2013 [4, 6], because genes of these 2 species were re-predicted using the NCBI Prokaryotic Genome Annotation Pipeline, which seemed to be more reasonable. Similarities between paired sequences were calculated using BLASTP with an E-value threshold of 1e-5. OrthoMCL (OrthoMCL DB: Ortholog Groups of Protein Sequences, RRID:SCR_007839) [41] was used to identify gene family based on the similarities of the genes and a Markov Chain Clustering (MCL) with default parameters. About 79.80% of *Cocos nucifera* genes were assigned to 14 411 families, of which 282 families only existed in *Cocos nucifera* (coconut specific families) (Table 7). Fig. 4 shows the shared gene families for orthologous genes. There are 544 orthologous families shared by 5 monocot species and 7706 orthologous families shared by all monocot and dicot species, suggesting 544 monocot unique functions shared by 5 monocot species and 7706 ancestral functions in the most recent common ancestor of the angiosperms.

**Table 7:** Statistical analysis of gene families of different species.

Species	Genes number	Genes in families	Unclustered genes	Family number	Unique families	Average genes per family
*C. nucifera*	28 039	22 376	5663	14 411	282	1.55
*E. guineensis*	30 430	22 021	8409	13 415	262	1.64
*P. dactylifera*	24 908	22 193	2715	14 074	112	1.58
*S. bicolor*	27 159	22 016	5143	12 992	916	1.69
*P. persica*	27 792	24 276	3516	14 443	497	1.68
*S. tuberosum*	34 879	28 288	6591	13 206	1119	2.14
*G. max*	42 859	38 104	4755	14 589	1145	2.61
*A. thaliana*	26 637	22 990	3647	13 292	674	1.73
*T. cacao*	28 624	23 776	4848	14 928	625	1.59
*V. vinifera*	25 329	19 122	6207	13 309	599	1.44
*M. acuminata*	36 538	24 354	12 184	13 089	620	1.86

### Phylogenetic analysis

We extracted 247 single-copy orthologous genes derived from the gene family analysis step, and then aligned the protein sequences of each family with MUSCLE (v3.8.31; MUSCLE, RRID:SCR_011812) [42]. Next, the protein alignments were converted to corresponding coding sequences (CDS) using an in-house Perl script. These coding sequences of each single-copy gene family were concatenated to form 1 super gene for each species. The nucleotides at positions 2 (phase 1 site) and 3 (4 degenerate sites) of codon were extracted separately to construct the phylogenetic tree by PhyML 3.0 (PhyML, RRID:SCR_014629) [43] using a HKY85 substitution model and a gamma distribution across sites. The tree constructed by phase 1 sites was consistent with the tree constructed by 4 degenerate sites.

### Divergence time

**Figure 5:**
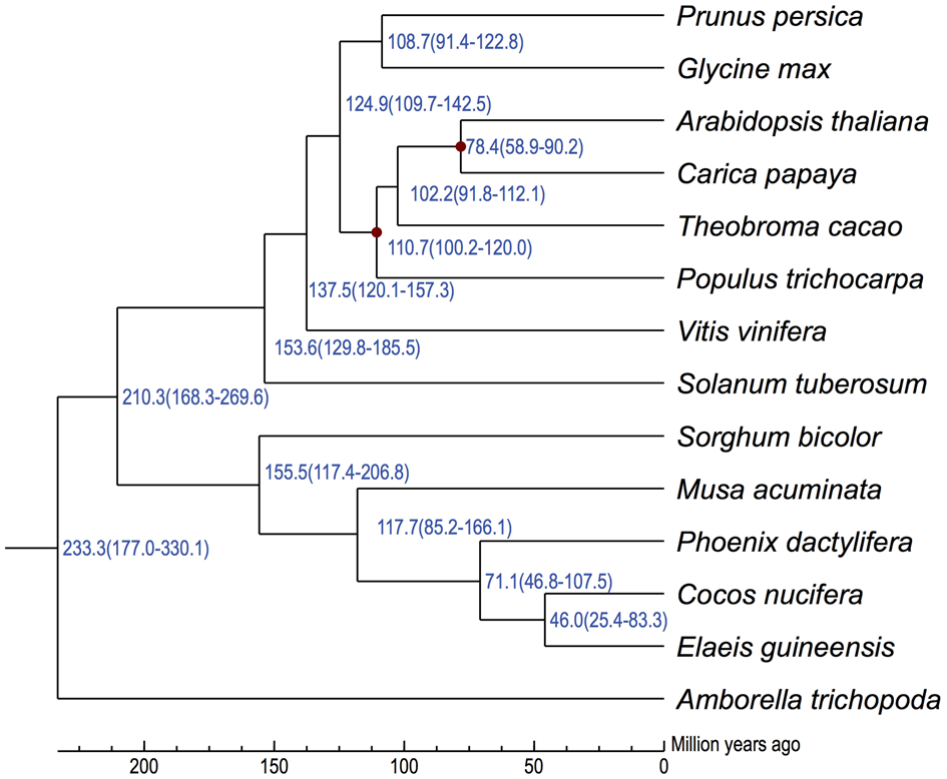
Estimation of divergence time. The blue numbers on the nodes are the divergence time from present (million years ago); the red nodes indicate the previously published calibration times.

The Bayesian relaxed molecular clock approach was used to estimate species divergence time using MCMCTREE in PAML (PAML, RRID:SCR_014932) [44], based on the 4 degenerate sites and the data set used in phylogenetic analysis, with previously published calibration times (divergence between *Arabidopsis thaliana* and *Carica papaya* was 54–90 Mya, divergence between *Arabidopsis thaliana* and *Populus trichocarpa* was 100–120 Mya) [45]. The divergence time between coconut and oil palm is about 46.0 Mya (25.4–83.3 Mya) (Fig. 5), which is less than the divergence time between coconut and date palm.

### Identification of antiporter genes in coconut genome

Antiporters are transmembrane proteins involved in the exchange of substances within and outside the membrane. In Arabidopsis, the functions of antiporter genes have been well characterized experimentally, and this gene family was subdivided into 13 different functional groups. Among them, 3 functional clusters were involved in Na^+^/H^+^ antiporters, some of which were documented to be associated with salt tolerance [46, 47].

The amino acid sequences of 70 *antiporter* genes of Arabidopsis were downloaded from the Arabidopsis Information Resource TAIR website (TAIR, RRID:SCR_004618) [48] and used as queries for BLASTP against the predicted proteins in the *Cocos nucifera* genome with a cut-off E-value of 1e-10. A total of 126 *antiporter* genes were identified in coconut genome. Using local Hidden Markov Model–based HMMER (v3.0) searches and the Pfam database, 7 antiporter genes were excluded from further analysis because of the lack of conserved domain. The detailed information of the 119 antiporter genes is listed in Additional file 1.

**Figure 6:**
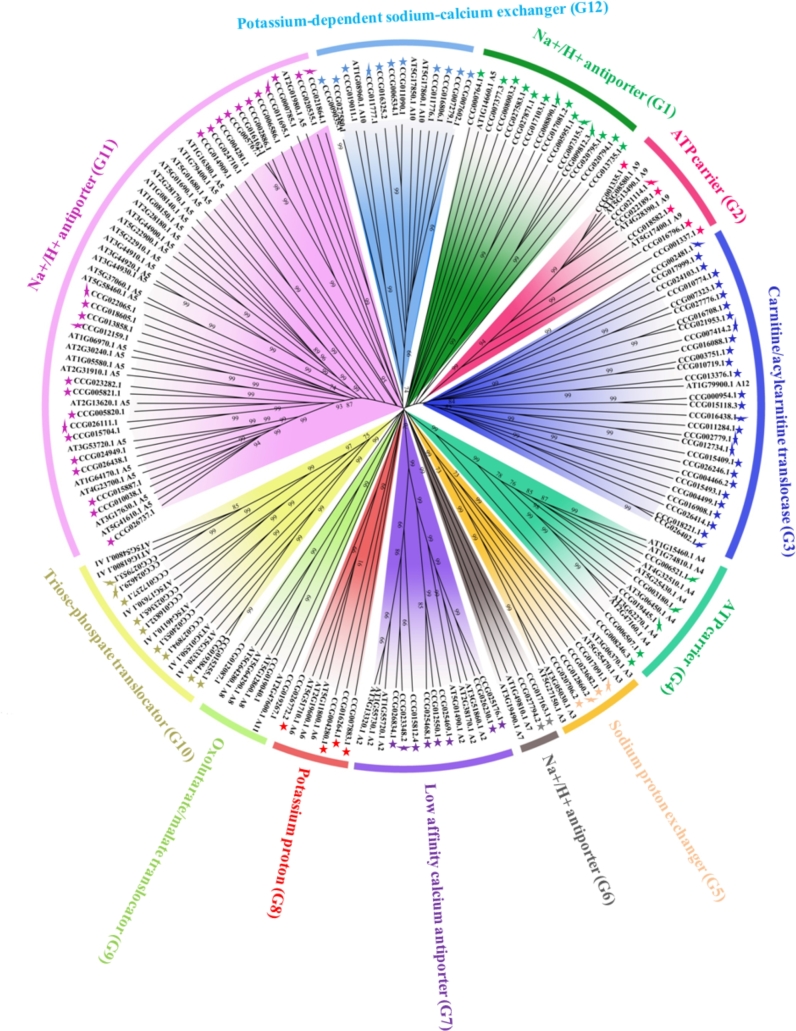
Phylogenetic tree of antiporter genes from *C. nucifera* and *Arabidopsis thaliana*. Every cluster is indicated with a different colored arc line arc. The potential function of every cluster is indicated with the function groups found in *Arabidopsis thaliana*. Colored stars indicate antiporter genes of *C. nucifera*.

In order to elucidate the evolutionary relationship and potential functions of the antiporters identified in the study, we applied phylogenetic analysis of *Arabidopsis* and *C. nucifera* antiporter proteins using the neighbor joining method (Fig. 6). Phylogenetic analysis showed that the 119 antiporter genes from *C. nucifera* can be subdivided into 12 groups and that almost all antiporter genes were clustered together with the functional groups in *Arabidopsis thaliana*.

Phylogenetic analysis showed that the number of antiporter genes was equal between *Arabidopsis thaliana* and *C. nucifera* for most groups, except for G1 (1 of 3 Na^+^/H^+^ antiporter family), G3 (carnitine/acylcarnitine translocase family), and G12 (potassium-dependent sodium-calcium exchanger). The G1 group (1 of 3 Na^+^/H^+^ antiporter families) contained only 1 Arabidopsis antiporter gene and but 14 *C. nucifera* antiporters (1-At/14-Cn), whereas G3 (carnitine/acylcarnitine translocase family) contained 1-At/29-Cn, and G13 (potassium-dependent sodium-calcium exchanger) contained 3-At/11-Cn. The Na^+^/H^+^ antiporter family had been reported to be associated with salt stress. The expansion of the Na^+^/H^+^ antiporter gene family in the coconut palm may be associated with the high salt tolerance of coconut. Meanwhile, carnitine/acylcarnitine translocase is involved in fatty acid transport across the mitochondrial membranes. This gene family expansion may be associated with accumulation of fatty acid in coconut pulp. Moreover, coconut water contains a high density of potassium ion, approximately 312 mg potassium ion per 100 g of coconut water [49]. In this study, the gene number of potassium-dependent sodium-calcium exchangers was also detected to be significantly increased compared to Arabidopsis.

### Identification of ion channel genes in coconut genome

**Figure 7:**
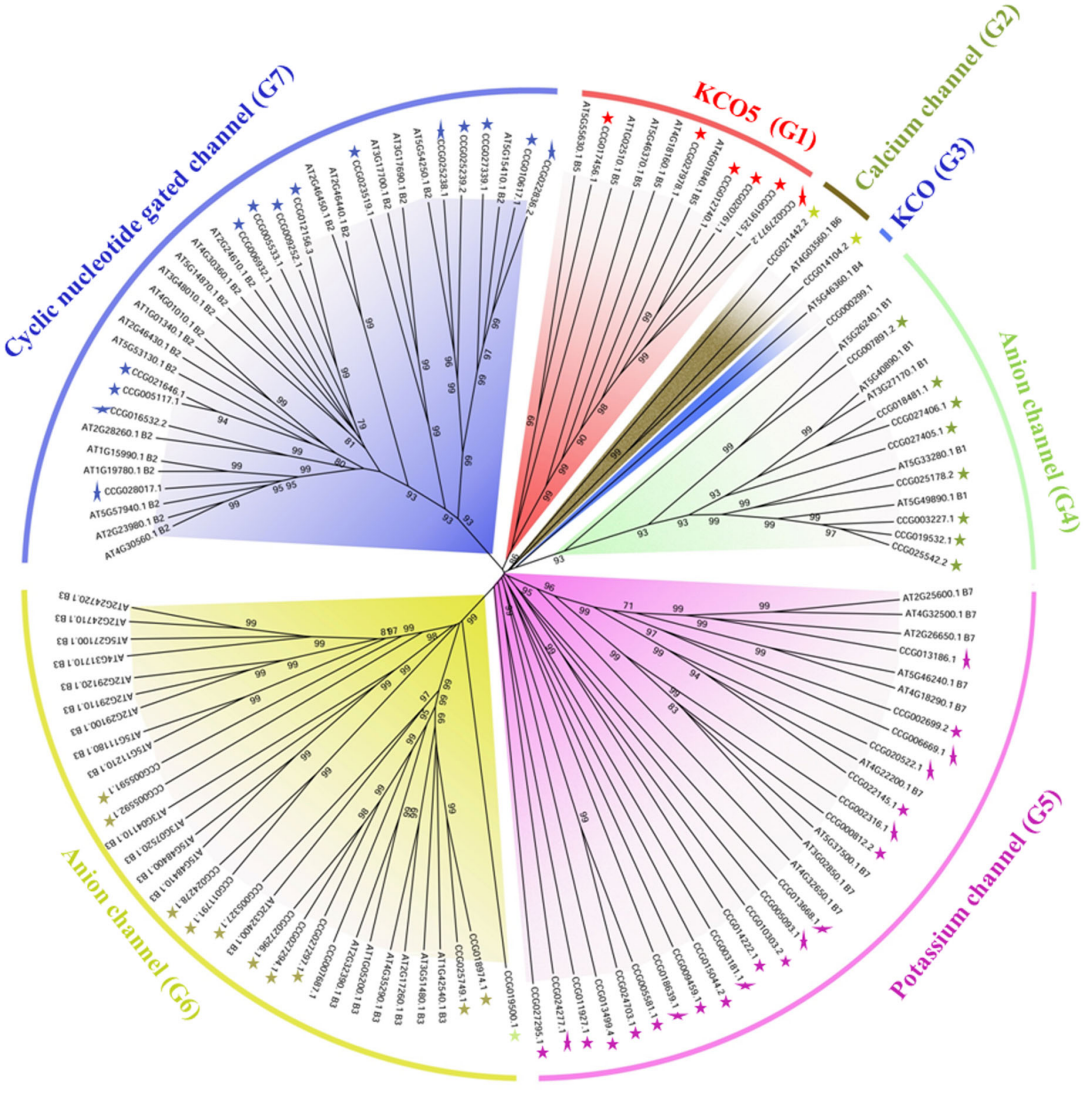
Phylogenetic tree of ion channel genes from *C. nucifera* and *Arabidopsis thaliana*. Every cluster was indicated with different colored arc line arc. The potential function of every cluster was indicated with the function groups found in *Arabidopsis thaliana*. Colored stars indicate ion channel genes of *C. nucifera*.

A total of 67 ion channel genes were identified in the coconut genome (Additional file 2). The amino acid sequences of 67 *C. nucifera* and 60 *Arabidopsis* ion channel genes were used to analyze their evolutionary relationship (Fig. 7). Almost all ion channel genes from *C. nucifera* can be clustered into the function groups found in *Arabidopsis thaliana*. The number of ion channel genes was equal between *Arabidopsis thaliana* and *Cocos nucifera* in most groups except for G5 (potassium channel). Many more genes (21) from *C. nucifera* than from *Arabidopsis thaliana* (9 genes) were present in group 5 (potassium channels). The gene family expansion may be associated with the accumulation of potassium ions in coconut water.

## Conclusion

*Cocos nucifera* (2n = 32) is an important tropical crop, and it is also used as an ornamental plant in the tropics. In the present study, we sequenced and *de novo* assembled the coconut genome. A total scaffold length of 2.2 Gb was generated, with scaffold N50 of 418 Kb. The divergence time of *Cocos nucifera* and *Elaeis guineensis* is more recent than that of *Cocos nucifera* and *Phoenix dactylifera*, suggesting a closer relationship between *C. nucifera* and *E. guineensis*. Comparative analysis of antiporter and ion channels between *C. nucifera* and *Arabidopsis thaliana* showed significant gene family expansions, maybe involving Na^+^/H^+^ antiporters, carnitine/acylcarnitine translocases, potassium-dependent sodium-calcium exchangers, and potassium channels. The expansion of these gene families may be associated with adaptation to salt stress, accumulation of fatty acid in coconut pulp, and potassium ions in coconut water. The data output of the coconut genome will provide a valuable resource and reference information for the development of high-density molecular makers, construction of high-density linkage maps, detection of quantitative trait loci, genome-wide association mapping, and molecular breeding.

## Availability of supporting data

Supporting data are available in the *Giga*DB database (*Giga*DB, RRID:SCR_004002) [50]. Raw data were deposited in the Sequence Read Archive (SRA539146) with the project accession code PRJNA374600 for the *Cocos nucifera* genome. Previously published RNA-seq data used for transcriptome-based prediction are available under accession number SRR606452.

## Availability of other angiosperms data sources

*Arabidopsis thaliana*, *Oryza sativa*, *Sorghum bicolor*, *Zea mays*, *Sorghum bicolor, Solanum tuberosum, Prunus persica*, *Theobroma cacao*, *Vitis vinifera*, *Musa acuminata*, *Carica papaya*, *Populus trichocarpa*, *Amborella trichopoda*: https://phytozome.jgi.doe.gov/pz/portal.html (phytozomev9.1)

*Elaeis guineensis*: ftp://ftp.ncbi.nlm.nih.gov/genomes/all/GCF/000/442/705/GCF_000442705.1_EG5/

*Phoenix dactylifera* (DPV01): ftp://ftp.ncbi.nlm.nih.gov/genomes/all/GCF/000/413/155/GCF_000413155.1_DPV01/

*Phoenix dactylifera* (PDK30): http://qatar-weill.cornell.edu/research/datepalmGenome/download.html

## Additional files

Additional file 1: Identification and characterization of antiporter genes in the genome of *Cocos nucifera*.

Additional file 2: Identification and characterization of ion channel genes in the genome of *Cocos nucifera*.

## Abbreviations

bp: base pair; CDS: coding sequence; CTAB: Cetyl trimethylammonium bromide; EG: Elaeis guineensis; Gb: giga base; HAT: Hainan Tall; Kb: kilo base; KEGG: Kyoto Encyclopedia of Genes and Genomes; LTRs: long-terminal repeat retrotransposon; Mb: mega base; MCL: Markov Chain Clustering; MP mate-pair; PE: paired-end; SRA: Seqeunce Read Archive; TE: transposable elements; UTRs: untranslated region.

## Competing interests

The authors declare that they have no competing interests.

## Funding

This study was supported by International Science and Technology Cooperation projects of Hainan Province (No. KJHZ2014–24), Hainan Natural Science Foundation (No. 313 058), the major Technology Project of Hainan (No. ZDZX2013023–1), the fundamental Scientific Research Funds for the Chinese Academy of Tropical Agriculture Sciences (CATAS-No. 1 630 032 012 044, 1 630 052 014 002, 1 630 052 015 050, 1 630 152 017 019, and 1 630 152 016 006), and the Central Public-interest Scientific Institution Basal Research Fund for Innovative Research Team Program of CATAS (No.17CXTD-28).

## Author contributions

Y.X., H.F., Y.Y., M.P., Q.L., and A.G. designed the study and contributed to the project coordination. X.Y., P.X., and W.X. wrote the paper. L.Z., J.L., and Y.W. collected the samples and extracted the genomic DNA. Y.X., B.L., B.S., J.X., A.A., E.I., and N.L. conducted the genome analyses.

## Supplementary Material

GIGA-D-17-00038_Original-Submission.pdf

GIGA-D-17-00038_Revision-1.pdf

GIGA-D-17-00038_Revision-2.pdf

Response-to-Reviewer-Comments_Original-Submission.pdf

Response-to-Reviewer-Comments_Revision-1.pdf

Reviewer-1-Report-(Original-Submission).pdf

Reviewer-2-Report-(Original-Submission).pdf

Reviewer-2-Report-(Revision-1).pdf

Reviewer-3-Report-(Original-Submission).pdf

Additional Files
