# Supplementary material for: The genome draft of coconut (Cocos nucifera)
Source: Gigascience. 2017 Oct 5;6(11):1–11. doi: 10.1093/gigascience/gix095 (PMC5714197; doi:10.1093/gigascience/gix095)
Supplement: GIGA-D-17-00038_Original-Submission.pdf [file gix095_giga-d-17-00038_original-submission.pdf]

GigaScience

The genome draft of the Coconut (Cocos nucifera)

--Manuscript Draft--

**Manuscript Number:**	GIGA-D-17-00038
**Full Title:**	The genome draft of the Coconut (Cocos nucifera)
**Article Type:**	Data Note
**Funding Information:**	International Science and Technology Cooperation projects of Hainan Province (No. KJHZ2014-24) — Dr Yaodong Yang<br>Hainan Natural Science Foundation (313058) — Dr Wei Xia<br>the fundamental Scientific Research Funds for Chinese Academy of Tropical Agriculture Sciences (1630032012044) — Dr Yaodong Yang<br>the fundamental Scientific Research Funds for Chinese Academy of Tropical Agriculture Sciences (1630052014002) — Dr Yaodong Yang<br>the fundamental Scientific Research Funds for Chinese Academy of Tropical Agriculture Sciences (1630052015050) — Dr Yong Xiao<br>The major Technology Project of Hainan (ZDZX2013023-1) — Dr Ming Peng
**Abstract:**	Background<br>The Coconut palm (Cocos nucifera, 2n = 32), a member of genus Cocos and of the family Arecaceae (Palmaceae), is an important tropical fruit and oil crop. Currently, this tropical tree crop is cultivated in 93 countries, including Central and South America, East and West Africa, Southeast Asia and the Pacific islands, with a total growth area of more than 11 million hectares. The Coconut palm can generally be classified into two main categories: "Tall"(flowering 8-10 years after planting) and "Dwarf" (flowering 4-6 years after planting), based on morphological characteristics and breeding habits. The long generational time of this tropical species hinders progress in genetic breeding. In spite of initial successes, genetic improvement is very slow.<br>Findings<br>A total of 714.67 gigabases (Gb) of raw data was acquired by the Illumina HiSeq 2000 platform, comprising approximately 285.86×coverage of the Cocos nucifera genome (variety "Hainan Tall"). After filtering the low quality reads, PCR duplication and small insert size, 419.08 gigabases (Gb) of clean data was obtained, these clean reads were assembled with SOAPdenovo2 [29]. A total scaffold length of 2.20 Gb was generated, with a scaffold N50 of 418 Kb, which represents 90.91% of the estimated genome (2.42Gb). BUSCO evaluation demonstrated the completeness of the coconut genome reached 90.8%. The coconut genome was predicted to harbor 28,039 protein-coding genes, which is less than Phoenix dactylifera (DPV01, 4,166) and Elaeis guineensis (34,802). The annotation completeness was also evaluated by BUSCO, reached 74.1%. Genome annotation results revealed that 72.75% of the coconut genome consists of transposable elements, among which long-terminal repeat elements (LTRs) make up the largest proportion (92.23%).<br>Conclusions<br>Despite its agronomic importance, Cocos nucifera is still under-studied. We report a genome draft of Cocos nucifera. This study provides a large amount of genomic information, facilitatingfuture functional genomics and molecular breeding in Cocos nucifera.
**Corresponding Author:**	Yaodong Yang, Ph.D<br>Coconut Research Institute<br>Wenchang City, Hainan CHINA
**Corresponding Author Secondary Information:**	

Powered by Editorial Manager® and ProduXion Manager® from Aries Systems Corporation

**Corresponding Author's Institution:**	Coconut Research Institute
**Corresponding Author's Secondary Institution:**	
**First Author:**	Yong Xiao
**First Author Secondary Information:**	
**Order of Authors:**	Yong Xiao
	Pengwei Xu
	Haikuo Fan
	Luc Baudouin
	Wei Xia
	Stéphanie Bocs
	Junyang Xu
	Qiong Li
	Anping Guo
	Lixia Zhou
	Jing Li
	Yi Wu
	Zilong Ma
	Alix Armero
	Auguste Emmanuel Issali
	Na Liu
	Ming Peng
	Yaodong Yang
**Order of Authors Secondary Information:**	
**Opposed Reviewers:**	
**Additional Information:**	
**Question**	**Response**
Are you submitting this manuscript to a special series or article collection?	No
**Experimental design and statistics**<br><br>Full details of the experimental design and statistical methods used should be given in the Methods section, as detailed in our Minimum Standards Reporting Checklist. Information essential to interpreting the data presented should be made available in the figure legends.<br><br>Have you included all the information requested in your manuscript?	Yes
**Resources**	Yes

A description of all resources used, including antibodies, cell lines, animals and software tools, with enough information to allow them to be uniquely identified, should be included in the Methods section. Authors are strongly encouraged to cite Research Resource Identifiers (RRIDs) for antibodies, model organisms and tools, where possible.<br><br>Have you included the information requested as detailed in our Minimum Standards Reporting Checklist?	
**Availability of data and materials**<br><br>All datasets and code on which the conclusions of the paper rely must be either included in your submission or deposited in publicly available repositories (where available and ethically appropriate), referencing such data using a unique identifier in the references and in the “Availability of Data and Materials” section of your manuscript.<br><br>Have you have met the above requirement as detailed in our Minimum Standards Reporting Checklist?	Yes

Powered by Editorial Manager® and ProduXion Manager® from Aries Systems Corporation

Manuscript Click here to download Manuscript manuscript-submit-cocont genome20170307.docx

The genome draft of Coconut (*Cocos nucifera*)

Yong Xiao[1]*, Pengwei Xu[3]*, Haikuo Fan[1]*, Luc Baudouin[4]*, Wei Xia[1]*, Stéphanie Bocs[4]*, Junyang Xu[3], Qiong Li[2], Anping Guo[2],Lixia Zhou[1],Jing Li[1], Yi Wu[1], Zilong Ma[2], Alix Armero[4], Auguste Emmanuel Issali[5], Na Liu[3&], Ming Peng[2&], Yaodong Yang[1&]

[1]Hainan Key Laboratory of Tropical Oil Crops Biology/Coconut Research Institute,Chinese Academy of Tropical Agricultural Sciences, Wenchang, Hainan 571339, P.R.China

[2]Institute of Tropical Bioscience and Biotechnology, Chinese Academy of Tropical Agricultural Science, Haikou, Hainan 571101, P. R. China

[3] BGI-Shenzhen, Shenzhen 518083, China

[4]French Agriculture Research Centre for International for International Development (CIRAD), UMR AGAP, F-34398, Montpellier France

[5]Station Cocotier Marc Delorme, Centre National De RechercheAgronomique (CNRA)07 B.P. 13, Port Bouet,Côte d'Ivoire

*The authors have equal contribution to the manuscript

[&]Corresponding author

Yong Xiao: xiaoyong1980@catas.cn

Wei PengXu: xupengwei@genomics.cn

Haikuo Fan: vanheco@163.com

Baudouin Luc:luc.baudouin@cirad.fr

Wei Xia: saizjxiawei@hainu.edu.cn

BocsStéphanie:stephanie.sidibe-bocs@cirad.fr

JunyangXu: xujy@genomics.cn

Qiong Li: liqiong4416@126.com

AnpingGuo: gap211@126.com

Lixia Zhou: glzz_2009@163.com

Jing Li: lijing002x@catas.cn

Yi Wu: wuyi-scuta@163.com

Zilong Ma: mzl900@163.com

ArmeroAlix:alix.armero_villanueva@cirad.fr

Auguste Emmanuel Issali: issaliemma@yahoo.com

Na Liu: naliu@genomics.cn

Ming Peng: mmpeng_2000@yahoo.com

Yaodong Yang: yyang@catas.cn

Background

The Coconut palm (*Cocos nucifera*, 2n = 32), a member of genus *Cocos* and of the family Arecaceae (Palmaceae), is an important tropical fruit and oil crop. Currently, this tropical tree crop is cultivated in 93 countries, including Central and South America, East and West Africa, Southeast Asia and the Pacific islands, with a total growth area of more than 11 million hectares. The Coconut palm can generally be classified into two main categories: "Tall"(flowering 8-10 years after planting) and "Dwarf" (flowering 4-6 years after planting), based on morphological characteristics and breeding habits. The long generational time of this tropical species hinders progress in genetic breeding. In spite of initial successes, genetic improvement is very slow.

Findings

A total of 714.67 gigabases (Gb) of raw data was acquired by the Illumina HiSeq 2000 platform, comprising approximately 285.86×coverage of the *Cocos nucifera* genome (variety "Hainan Tall"). After filtering the low quality reads, PCR duplication and small insert size, 419.08 gigabases (Gb) of clean data was obtained, these clean reads were assembled with SOAPdenovo2 [29]. A total scaffold length of 2.20 Gb was generated, with a scaffold N50 of 418 Kb, which represents 90.91% of the estimated genome (2.42Gb). BUSCO evaluation demonstrated the completeness of the coconut genome reached 90.8%. The coconut genome was predicted to harbor 28,039 protein-coding genes, which is less than *Phoenix dactylifera* (DPV01, 4,166) and *Elaeis guineensis* (34,802). The annotation completeness was also evaluated by BUSCO, reached 74.1%. Genome annotation results revealed that 72.75% of the coconut genome consists of transposable elements, among which long-terminal repeat elements (LTRs) make up the largest proportion (92.23%).

Conclusions

Despite its agronomic importance, *Cocos nucifera* is still under-studied. We report a genome draft of *Cocos nucifera*. This study provides a large amount of genomic information, facilitatingfuture

functional genomics and molecular breeding in *Cocos nucifera*.

Keywords:Coconut palm genome, Assembly, Annotation

Data description

Background

Coconut (*Cocos nucifera*, 2n = 32), the only species of genus *Cocos* of family Arecaceae, is a tropical oil crop and is widely cultivated in tropical regions due to its extensive application in agriculture and industry. The tropical species was thought to have originated from the western pacific region (including Malay Peninsula and archipelago, New Guinea, and the Bismarck Archipelago) and the southwest Pacific. Presently, the tropical tree crop had been distributed across 89 tropical countries, including Central and South American, East and West African, Southeast Asia and the pacific islands, and accounts for over 12 million hectares of land.

In China, the coconut palm grows in the Hainan and Yunnan provinces as an economic and ornamental plant. In the province of Hainan, coconut is cultivated over an area of approximately 43,000 hectares, of which approximately 36,000 hectares is made up by the coconut variety “Hainan Tall” (HAT) [1].Hainan Tall coconut are slow to mature (flowering 8-10 years after planting), can grow to a height of about 20-30 meters, and have medium to large nut size. Hainan Tall coconuts can adapt to a wide range of environment and have tolerance to biotic and abiotic stress, especially for high tolerant to high salt density. The morphological characteristics of Hainan Tall coconut were showed in Figure 1. Here we present Hainan Tall coconut genome sequence, making it possible to understand its adaption to high salinity. Moreover, the draft genome sequence of its relative species, *Elaeis guineensis* and *Phoenix dactylifera*, were also reported. The comparative analysis was performed about genome assembly and annotation between coconut and its relative species in the study.

Sample collection and sequencing

Genomic DNA was extracted from the spear leaf of a “Hainan Tall” coconut (*Cocos nucifera* L. Taxonomy ID: 13894; 19033’3” N, 110047’25” E) individual selected from the coconut garden of the Coconut Research Institute (Wenchang, Hainan province, China) using the CTAB extraction method [2]. Subsequently, four pair-end (PE) libraries with insert size 170 bp, 500 bp, 450 bp and 800 bp and

five Mate-pair (MP) libraries with insert size 2 Kb, 5Kb, 10Kb, 20Kb and 40 Kb were constructed using the standard procedure provided by Illumina (San Diego, USA). After library preparation and quality control of DNA samples, template DNA fragments were hybridized to the surface of flow cells on an IlluminaHiSeq2000sequencer, amplified to form clusters, and sequenced following the standard Illumina manual. Finally, we generated 714.67 Gb of raw reads from all constructed libraries, raw sequenced outputs for each library are summarized in Table 1. Before assembly, reads with low quality (base quality less than 7 with percent higher than 25% or N percent higher than 1%), PCR duplication or adapter contamination were removed by using SOAPfilter, a software application in the SOAPdenovo package [3]. After filtering, 419.08 Gb (173.17×) high-quality sequences were obtained for genome assembly.

De novo* assembly of short reads of *Cocos nucifera

We used clean reads of the short-insert libraries (170bp) to estimate the coconut genome size by k-mer frequency distribution analysis [3]. The genome size (G) of *Cocos nucifera* could be estimated by the following formula:

$$G = N \times (L - 17 + 1)/K\_depth$$

where N represents the total of number of reads, L represents the read length and K_depth refers to the main peak in the k-mer distribution curve. In our calculations, N was 2,049,520,223, L was 100 and K_depth was 71, therefore *Cocos nucifera* genome was estimated to be 2.42 gigabases (Gb). K-mer size distribution analysis (Figure 2) indicated that *Cocos nucifera* was a diploid species with low heterozygous and high repetitive sequence.

We then assembled the Cocos nucifera genome using SOAPdenovo2 in three steps: contig construction, scaffold construction and gap filling. In the contig construction step: the SOAPdenovo2 with parameters “pregraph-K 63 -R -d 1” was employed to construct de Bruijn graphs from pair-end libraries with an insert size from 170 to 800 bp. Then the kmers from the de Bruijn graphs were used to form contiguous sequences (contigs) with the parameters “contig -R” by clipping tips, merging bubbles and removing the low coverage links. In the scaffold construction step: the orders of the contigs were

determined using paired-end and mate-pair information with parameters “map -k 43”and“scaff -F -u”. Initially, SOAPdenovo2 map the reads from pair-end and mate pair libraries to contigs based on a hash table (keys are unique k-mers on contigs; values are positions). In this case, two contigs are considered to be linked if the number of read pairs bridging the contigs exceeds the threshold three. Gaps within scaffolds were filled by utilizing KGF [4] (V1.06) and GapCloser software (v1.12-r6) [5] with pair-end libraries with an insert size from 170 to 800 bp in cases where one end could be mapped to one contig and the other end extended into a gap. To achieve optimal assembly result, Rabbit (a Poisson-based K-mer model, ftp://ftp.genomics.org.cn/pub/Plutellaxylostella/) was used to determine repeat sequences, segmental duplications or divergent haplotypes on the assembly. After removal of redundant sequences, a total scaffold length of 2.20 Gb was generated, comprising 90.91% of the predicted genome size (Table 2), which was larger than the other species in palmae. Meanwhile, the obtained contig N50 was 72.64 Kb and the scaffold N50 was 418.06 Kb while the length of scaffolds less than 100 bp were excluded. Comparison of coconut assembly N50s with four previously published palm genomes *Phoenix dactylifera* (PDK30) [6], *Phoenix dactylifera* (DPV01) [7], *Elaeis guineensis* [8] and *Elaeis oleifera* [8] confirmed that our results were better quality (Table 3).

Genome evaluation

The 57,304 unigenes (transcript obtained from three different issues, spear leaves, young leaves and fruit flesh) reported in previous Fan’s research [9] were aligned to the assembled genome of *Cocos nucifera* using BLAT [10] with threshold “E-value = 10e-6, identity = 90%, coverage >90%”. The alignment results predicted that the assembled genome of *Cocos nucifera* covered 96.78% of all expressed unigenes, suggesting a high level of coverage (Table 4).

We also evaluated the completeness of the assembly using BUSCO [11], which quantitatively assess genome completeness using evolutionarily-informed expectations of gene content from near-universal single-copy orthologs selected from OrthoDB v9 (http://busco.ezlab.org/, plant set). BUSCO analysis showed that 90.8 and 3.4% of the 1,440 expected plant genes were identified as complete and fragmented, respectively, while 5.8 % were considered missing in the assembly. The BUSCO results showed that our assembly was more complete than the previously reported data palm and oil palm genome assembly (Table 5).

Repeat annotation

We combined a homology and *de novo* method to identify transposable elements (TEs) and the tandem

repeats in the *Cocos nucifera* genome. In homology step: TEs at DNA and protein levels were identified by searching against Repbase library (version 20.04) [12] with RepeatMasker (version 4.0.5) [13] and RepeatProteinMasker (v4.0.5) [13]. In *de novo* step: *de novo* libraries were constructed based on the genome sequences using the *de novo* prediction program RepeatModeler (http://www.repeatmasker.org/RepeatModeler.html,version1.0.5) and LTR_FINDER [14] by removing contamination and multi-copy genes. Then the novel transposable elements were identified and classified using RepeatMasker. The tandem repeat sequences were identified by TRF (Tandem Repeat Finder) software [15] with the following parameters "Match=2, Mismatch=7, Delta=7, PM=80, PI=10, Minscore=50 and MaxPerid =2000". The total length of the tandem repeat sequences predicted by the software is 151,229,585 bp, comprising 6.86% of the coconut genome. Finally, a total of 1.6 Gb of non-redundant repetitive elements were identified, accounting for 74.48% of the coconut genome, while transposable elements took up 72.75%. The most predominant transposons were long-terminal repeat (LTR), which account for 92.23% of all TEs and 67.1% of the coconut genome (Table 6).

Gene prediction

We combined homology, *de novo* and transcript alignment to predict genes in *Cocos nucifera* genome. For homology prediction: The gene sets of *Arabidopsis thaliana* [16], *Oryza sativa* [17], *Sorghum bicolor* [18] and *Zea mays* [19] were downloaded from the phytozomev9.1 (https://phytozome.jgi.doe.gov/pz/portal.html). The gene sets of *Elaeis guineensis* and *Phoenix dactylifera* (DPV01) were download from the NCBI ftp site. The longest transcript was selected to represent the genes with alternative splicing variants. We aligned these homologous proteins to the coconut genome using TBLASTN [20] with parameter "-e 1e-5 -F -m 8", and connected the BLSAT hit results to candidate gene loci by SOLAR with parameter "-a prot2genome2 -z" (https://sourceforge.net/p/treesoft/code/HEAD/tree/branches/lh3/solar/). Next, we extracted the genomic sequences of candidate gene loci with up and down stream 1k flanking sequences, applying Genewise 2.2.0 [21] to define the intron-exon boundary. The genes with pre-stop codon or frame-shifted were excluded for further analysis.

For *De novo* prediction: We randomly selected 1000 full length genes (GeneWise score equal 100, intact structure: start codon, stop codon, perfect intron-exon boundary) from gene sets predicted by homology method to train the model parameters for AUGUSTUS2.5 [22]. Two software programs, AUGUSTU2.5 and GENSCAN 1.0 [23], were used to do *de novo* prediction on repeat-masked genome

of *Cocos nucifera*. Genes with incomplete structure or protein coding length less than 150bp were filtered out.

Then Genes from homology and *de novo* method were combined to get non-redundant gene sets by using GLEAN [24] with the following parameters: minimum coding sequence length 150 bp and maximum intron length 50 kb. Genes were filtered with the same thresholds used for homology annotation.

For transcriptome-based prediction: RNA-seq data (SRR606452) from previous Fan's study were mapped onto the coconut genome to identify the splice junction using the software Tophat [25]. And then cufflinks [26] was used to assemble transcripts by the aligned reads. The coding potential of these transcripts was identified using fifth-order Hidden Markov Model, which was estimated with the same gene sets used in AUGUSTUS training by trainGlimmerHMM, a application in the GlimmerHMM [27] package. The transcripts with intact open reading frames (ORFs) were exacted and the longest ORF was retrieved while multiple isoforms located in the same locus.

At last, we merged the GLEAN and the transcriptome result to form a comprehensive gene set using an in-house annotation pipeline. If a transcript gene model had not overlapped with the GLEAN result, the transcriptome result would be added to the GLEAN result. If a transcript gene model had overlapped (overlap length >100bp), the transcriptome result would be used to perfect the structure of the GLEAN result (such as adding the un-translated regions (UTRs), completing the exon boundary). A diagrammatic pipeline is shown in Figure 3.

Gene evaluation

After all these steps, we obtained a final gene set contained 28,039 genes (Table 5), which is less than the gene numbers of *Phoenix dactylifera* (DPV01, 41,660) and *Elaeis guineensis* (34,802). Meanwhile the BUSCO evaluation demonstrated 74.1 and 11.2% of 1,440 expected plant genes were identified as complete and fragmented, 14.7% genes were considered missing in the gene sets. The BUSCO results showed that our gene prediction was more completely than *Phoenix dactylifera* (PDK30) and *Elaeis guineensis*, less completely than *Phoenix dactylifera* (DPV01) (Table 7), maybe the higher repetitive elements hinder the gene prediction of coconut genome.

Gene Function

Gene function annotation was identified bysequence similarity and domains conservation. In sequence similarity step: we searched the coconut protein coding against KEGG protein [28], SwissProt and TrEMBL [29] using BLASTP at a cut-off E-value threshold of 10^{-5}. Then we use the best match of alignment to represent the gene function. We obtained 18,445 KEGG, 18,867 Swissprot and 24,882 Tremble annotated genes. In domains conservation step: InterProScan5.11-51.0 software [30] was employed to identify the motif and domain against the public databases Pfam [31], PRINTS [32], ProDom [33], SMART [34], PANTHER [35], TIGRFAM [36] and SUPERFAMILY [37]. This revealed that 21,087 of the coconut proteins had conserved motifs, 1,622 Gene Ontology (GO) terms were assigned to 15,705 coconut proteins from the corresponding InterPro entry [38].. In total, approximately 89.41% of these genes were functionally annotated using above methods,

Conclusion

Cocos nucifera (2n = 32) is an important tropical crop, and is also used as an ornamental plant in the tropics. In the present study, we sequenced and *de novo* assembled the coconut genome. A total scaffold length of 2.2 Gb was generated, with a scaffold N50 of 418 Kb. The data output of the coconut genome will provide a valuable resource and reference information for the development of high density molecular makers, construction of high density linkage maps, detection of QTL (quantitative trait loci), genome-wide association mapping , and molecular breeding.

Availability of supporting data

Supporting data are available in the GigaDB database [39], and the raw data were deposited in the SRA539146 with the project accession PRJNA374600 for *Cocosnucifera* genome. Previously published RNA-seq data used for transcriptome-based prediction is available from the accession number SRR606452.

Competing interests

The authors declare that they have no competing interests.

Funding

This study was supported by International Science and Technology Cooperation projects of Hainan Province (No. KJHZ2014-24), Hainan Natural Science Foundation (No. 313058), The major

Technology Project of Hainan (No. ZDZX2013023-1), the fundamental Scientific Research Funds for Chinese Academy of Tropical Agriculture Sciences (CATAS-No. 1630032012044, 1630052014002, and 1630052015050)，Central Public-interest Scientific Institution Basal Research Fund for Innovative Research Team Program of CATAS (NO. 17CXTD-28).

Author’s contribution

YX, HF, YY, MP, QL, AG designed the study and contribute to the project coordination**;** XY, PX, WX wrote the paper; LZ, JL, YW collected the samples and extracted the genomic DNA; YX, BL, BS, JX, AA, EI, NL conducted the genome analyses.

Acknowledgements

Annaliese S. Mason is gratefully acknowledged for assistance with language editing and manuscript revisions.

References

1. Tang BN, Tang M, Chen CF, Qiu PH, Liu Q, Wang M, Li CE. Characteristics of soil fauna community in the Dongjiao coconut plantation ecosystem in Hainan, China Acta Ecologica Sinica. 2006; 26: 26-32.
2. Murray MG, Thompson WF. Rapid isolation of high molecular weight plant DNA. Nucleic Acids Research. 1980; 8:4321-4325.
3. Luo R, Liu B, Xie Y, Li Z, Huang W, Yuan J, He G, Chen Y, Pan Q, Liu Y.SOAPdenovo2: an empirically improved memory-efficient short-read denovo assembler. Gigascience.2012;1(1):1.
4. Short Oligonucleotide Analysis Package homepage: http://soap.genomics.org.cn/. Accessed 16 June 2016.
5. SOAPdenovo2 GitHub: https://github.com/gigascience/paper-luo2012.Accessed 16 June2016.
6. AI-Dous EK, George B, AI-Mahmoud ME, AI-Jaber MY, Wang H, Salameh YM, et al. De novo genome sequencing and comparative genomics of date palm (*Phoenix dactylifera*). Nat Biotechnol. 2011; 29: 521-527.
7. Al-Mssallem, I. S. et al. Genome sequence of the date palm Phoenix dactylifera L. Nat. Commun. 4:2274 doi: 10.1038/ncomms3274 (2013).
8. Singh R, Ong-Abdullah M, Low E-TL, Manaf MAA, Rosli R, Nookiah R, et al. Oil palm genome sequence reveals divergence of interfertile species in Old and New Worlds. Nature. 2013; 500: 335-341.
9. Fan H et al., RNA-Seq analysis of Cocosnucifera: transcriptome sequencing and de novo assembly

for subsequent functional genomics approaches.PLoS One, 2013 Mar29;8(3):e59997

10. Kent WJ. BLAT-the BLAST-like alignment tool. Genome Res. 2002; 12: 656-664
11. Simão FA, Waterhouse RM, Ioannidis P, Kriventseva EV, Zdobnov EM. BUSCO: assessing genome assembly andannotation completeness with singlecopyorthologs. Bioinformatics. 2015; 31(19): 3210-3212.
12. Jurka J, Kapitonov VV, Pavlicek A, Klonowski P, Kohany O, Walichiewicz J. Repbase Update, a database of eukaryotic repetitive elements. Cytogenet Genome Res. 2005; 110: 462-467.
13. Tarailo-Graovac M, Chen N. Using RepeatMasker to identify repetitive elements in genomic sequences. CurrProtoc Bioinformatics. 2009; Chapter 4: Unit 4. 10.
14. Xu Z, Wang H. LTR_FINDER: an efficient tool for the prediction of full-length LTR retrotransposons. Nucleic Acids Res. 2007; 35: W265-268.
15. Benson G, Tandem repeats finder: a program to analyze DNA sequence. Nucleic Acid Res. 1999; 27: 573-580.
16. Kaul S, Koo HL, Jenkins KJ, Rizzo M, Rooney T, Tallon LJ et al. Analysis of the genome sequence of the flowering plant *Arabidopsis thaliana*. Nature. 2000; 408: 796-815.
17. Goff SA, Ricke D, Lan TH, Presting G, Wang R, Dunn M, et al. A draft sequence of the rice genome (*Oryza sativa* L. ssp. *japonica*). Science. 2002; 296: 92-100.
18. Paterson AH, Bowers JE, Bruggmann R, Dubchak I, Grimwood J, Gundlach H, et al. The *Sorghum bicolor* genome and the diversification of grasses. Nature. 2009; 457: 551-556.
19. Schnable PS, Ware D, Fulton RS, Stein JC, Wei F, Pasternak S, et al. The B73 maize genome: complexity, diversity, and dynamics. Science. 2009; 326: 1112-1115.
20. Altschul SF, Madden TL, Schaffer AA, Zhang JH, Zhang Z, Miller W,Lipman DJ. Gapped BLAST and PSI-BLAST: a new generation of protein database serch programs. Nucleic Acids Research. 1997; 25: 3389-3402.
21. Birney E, Clamp M, Durbin R. GeneWise and Genomewise. Genome Res. 2004; 14: 988-995.
22. Stanke M, Keller O, Gunduz I, Hayes A, Waack S, Morgenstern B. AUGUSTUS: ab initio prediction of alternative transcripts. Nucleic Acid Res. 2006; 34: W435-439.
23. Burge C, Karlin S. Prediction of complete gene structures in human genomic DNA. J Mol Biol. 1997; 268: 78-94.
24. Elsik CG, Mackey AJ, Reese JT, Milshina NV, Roos DS, Weinstock GM. Creating a honey bee

consensus gene set. Genome Biol. 2007; 8: R13.

25. Trapnell C, Pachter L, Salzberg SL. TopHat: discovering splice junctions with RNA-Seq. Bioinformatics. 2009; 25: 1105-1111.

26. Trapnell C, Williams BA, Pertea G, Mortazavi A, Kwan G, van Baren MJ, Salzberg SL, Wold BJ, PachterL.Transcript assembly and quantification by RNA-Seq reveals unannotatedtranscriptsand isoform switching during cell differentiation. Nature Biotechnology. 2010;28:511–5.

27. Majoros WH, Pertea M, Salzberg SL. TigrScan and GlimmerHMM: two opensource ab initio eukaryotic gene-finders. Bioinformatics. 2004;20(16):2878-9.

28. Ogata H, Goto S, Sato K, Fujibuchi W, Bono H, Kanehisa M. KEGG: Kyoto Encyclopedia of Genes and Genomes. Nucleic Acids Res. 1999; 27: 29-34.

29. Bairoch A, Apweiler R. The SWISS-PROT protein sequence database and its supplement TrEMBL in 2000. Nucleic Acid Res. 2000; 28: 45-48.

30. Jones P, Binns D, Chang H-Y, Fraser M, Li W, McAnulla C, McWilliam H, Maslen J, Mitchell A, Nuka G. InterProScan 5: genome-scale protein function classification. Bioinformatics. 2014; 30(9):1236–40.

31. Bateman A, Birney E, Durbin R, Eddy SR, Howe KL, Sconnhammer EL. The Pfam protein families database. Nucleic Acids Res. 2000; 28: 263-266.

32. Attwood TK, Cronig MD, Flower DR, Lewis AP, Madey JE, Scordis P, et al. PRINTS-S: the database formerly known as PRINTS. Nucleic Acids Res. 2000; 28: 225-227.

33. Corpet F, Gouzy J, Kahn D. Recent improvements of the ProDom database of protein domain families. Nucleic Acids Res. 1999: 27: 263-267.

34. Schult J, Copley RR, Doerks T, Ponting CP, Bork P. SMART: a web-based tool for the study of genetically mobile domains. Nucleic Acids Res. 2000; 28: 231-234.

35. PANTHER version 11: expanded annotation data from Gene Ontology and Reactome pathways, and data analysis tool enhancements. Huaiyu Mi, Xiaosong Huang, Anushya Muruganujan, Haiming Tang, Caitlin Mills, Diane Kang, and Paul D. Thomas Nucl. Acids Res. (2016) doi: 10.1093/nar/gkw1138

36. Selengut JD, Haft DH, Davidsen T, Ganapathy A, Gwinn-Giglio M, Nelson WC, Richter AR, White O. TIGRFAMs and Genome Properties: tools for the assignment of molecular function and biological process in prokaryotic genomes. Nucleic Acids Res. 2007 Jan;

37. Wilson, D, Pethica, R, Zhou, Y, Talbot, C, Vogel, C, Madera, M, Chothia, C, Gough, J (2009). SUPERFAMILY--sophisticated comparative genomics, data mining, visualization and phylogeny. Nucleic Acids Research. 37 (Database issue): D380-6. doi:10.1093/nar/gkn762.

38. Burge S, Kelly E, Lonsdale D, Mutowo-Muellenet P, McAnulla C, Mitchell A. Manual GO annotation of predictive protein signatures: the InterPro approach to GO curation. Database. 2012; 2012: 257-264.

39.

Tables

Table 1 Sequencing libraries and data yields for whole genome sequencing

Library tpe	Reads Length(bp)	Insert Size(bp)	Raw data (Gb)	Clean data(Gb)
PE101	100	170	128.75(53.20)	111.32(46)
PE251	250	450	73.86(30.52)	56.42(23.31)
PE101	100	500	64(26.45)	65.11(26.90)
PE101	100	800	78.16(32.30)	64.90(26.82)
PE50	49	2000	128.6(53.14)	60.70(25.08)
PE50	49	5000	71.75(29.65)	18.62(7.69)
PE50	49	10000	74.65(30.85)	18.53(7.66)
PE50	49	20000	70.7(29.21)	19.35(7.99)
PE50	49	40000	24.2(10.08)	4.13(1.71)

Note: The sequencing depth was shown in parentheses, calculated based on a genome size of 2.42G. Clean data were obtained by filtering raw data with low-quality and duplicate reads.

Table 2 Comparison of genome features of four palmae species

Genome features	PDK30	DPV01	EG	EO	Coconut
Assembly size (G)	0.38	0.56	1.54	1.40	2.20
Scaffold N50 (kb)	30.48	334.08	1045.41	333.11	418.07
Contig N50 (kb)	6.44	10.81	9.37	8.45	72.64
Gene Number	2,949	41,660	34,802	-	28,039
TEs percent (%)	23.6	38.87	43.24	-	72.75

Note: Coconut: *Cocos nucifer* (Hai nan Tall); PDK30: *Phoenix dactylifera* (PDK30); DPV01: *Phoenix dactylifera* (DPV01); EG: *Elaeis guineensis* (American oil palm E5 build); EO *Elaeis oleifera* (American oil palm, O8-build); The TEs result was obtained using the same pipeline with the Coconut

Table 3 Summary statistics of five palmae species

Species	Sequencing technology	Sequence coverage	Estimated size(Gb)	Assembly size(Gb)	Contig N50(Kb)	Scaffold N50(Kb)
Phoenix dactylifera (PDK30)	Illumina GAIIx	53.4x	0.66	0.38	6.44	30.48
Phoenix dactylifera (DPV01)	454,SOLiD, ABI3730	139x	0.67	0.56	10.81	334.08
Elaeis guineensis (African oil palm)	454	16X	1.8	1.54	9.37	1045.41
Elaeis oleifera (American oil palm)	454	16x	1.8	1.40	8.45	333.11
Cocos nucifera (Hai nan Tall)	Illumina HiSeq	173X	2.42	2.20	72.64	418.07

Table 4 The genome BUSCO assessment of palmae species

	Coconut		PDK30		DPV01		EG		EO	
BUSCOs	N	P (%)	N	P (%)	N	P (%)	N	P (%)	N	P (%)
Total	1440		1440		1440		1440		1440	
Complete single-copy	1192	82.8	1042	72.4	1160	80.6	1100	76.4	1004	69.7
Complete duplicated	115	8.0	81	5.6	134	9.3	116	8.1	63	4.4
Fragment	49	3.4	98	6.8	42	2.9	60	4.2	84	5.8
Missing	84	5.8	219	15.2	104	7.2	164	11.3	289	20.1

Note: Coconut: *Cocos nucifer* (Hai nan Tall); PDK30: *Phoenix dactylifera* (PDK30); DPV01: *Phoenix dactylifera* (DPV01); EG: *Elaeis guineensis* (American oil palm E5 build); EO *Elaeis oleifera* (American oil palm, O8-build);

Table 5 The gene coverage of *Cocos nucifera* by transcriptome data

Dataset	Number	Total length (bp)	Base coverage by assembly	Sequence coverage by assembly (%)
All	57,304	43,090,665	96.78	99.57
>200bp	57,304	43,090,665	96.78	99.57
>500bp	25,713	33,470,388	96.36	99.85
>1000bp	13,796	25,004,919	95.99	99.94

Table 6 Transposable elements in the coconut genome

	Repabse TEs	Protein TEs	*De novo* TEs	Combined TEs	
	length	length	length	length	percentage
DNA	20,936,158	24,655,089	35,131,002	58,119,982	2.64
LINE	4,251,185	9,631,472	7,610,172	19,197,064	0.87
SINE	85,717	0.00	186,364	270,055	0.012
LTR	361,968,154	512,700,933	1,419,281,798	1,478,182,089	67.10
Other	8,145	0.00	0.00	8,145	0.0004
Unknown	0.00	12,360	139,084,335	139,096,695	6.31
Total	385,037,442	546,965,774	1,552,582,881	1,602,630,396	72.75

Note: Repabse TEs means RepeatMask against Repbase; Protein TEs means RepeatProteinMask result against Repbase protein; *De novo* TEs means RepeatMask against the *de novo* library; Combined TEs means the combine results of three steps.

Table 7 The gene BUSCO assessment of palmae species

	Coconut		PDK30		DPV01		EG	
BUSCOs	N	P (%)	N	P (%)	N	P (%)	N	P (%)
Total	1440		1440		1440		1440	
Complete single-copy	965	74.1	748	51.9	1195	83.0	555	38.5
Complete duplicated	102	7.1	81	5.6	159	11.0	53	3.7
Fragment	162	11.2	255	17.7	44	3.1	270	18.8

Missing	211	14.7	356	24.8	42	2.9	562	39.0

Note: Coconut: *Cocos nucifer* (Hai nan Tall); PDK30: *Phoenix dactylifera* (PDK30); DPV01: *Phoenix dactylifera* (DPV01); EG: *Elaeis guineensis* (American oil palm E5 build); The gene of *Elaeis oleifera* (American oil palm, O8-build) was missing, not attained from the public database;

Figure legends

Figure 1 Morphological characteristics of coconut tree (A) and coconut tree bearing nuts (B).

Figure 2 Kmer analysis of the coconut genome.

Figure 3 The pipeline for integrating GLEAN and Transcriptome data.

Figure 1

Click here to download Figure Fig.1.pdf

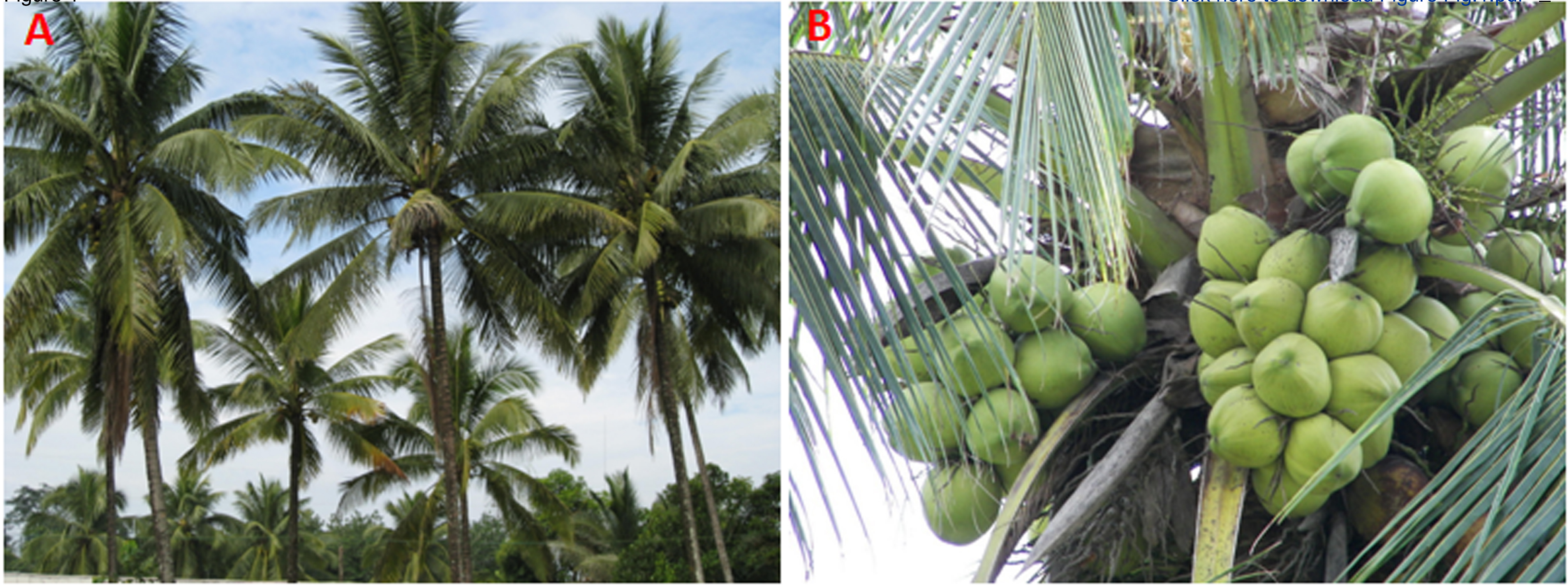

Figure 2

Click here to download Figure Fig.2.pdf

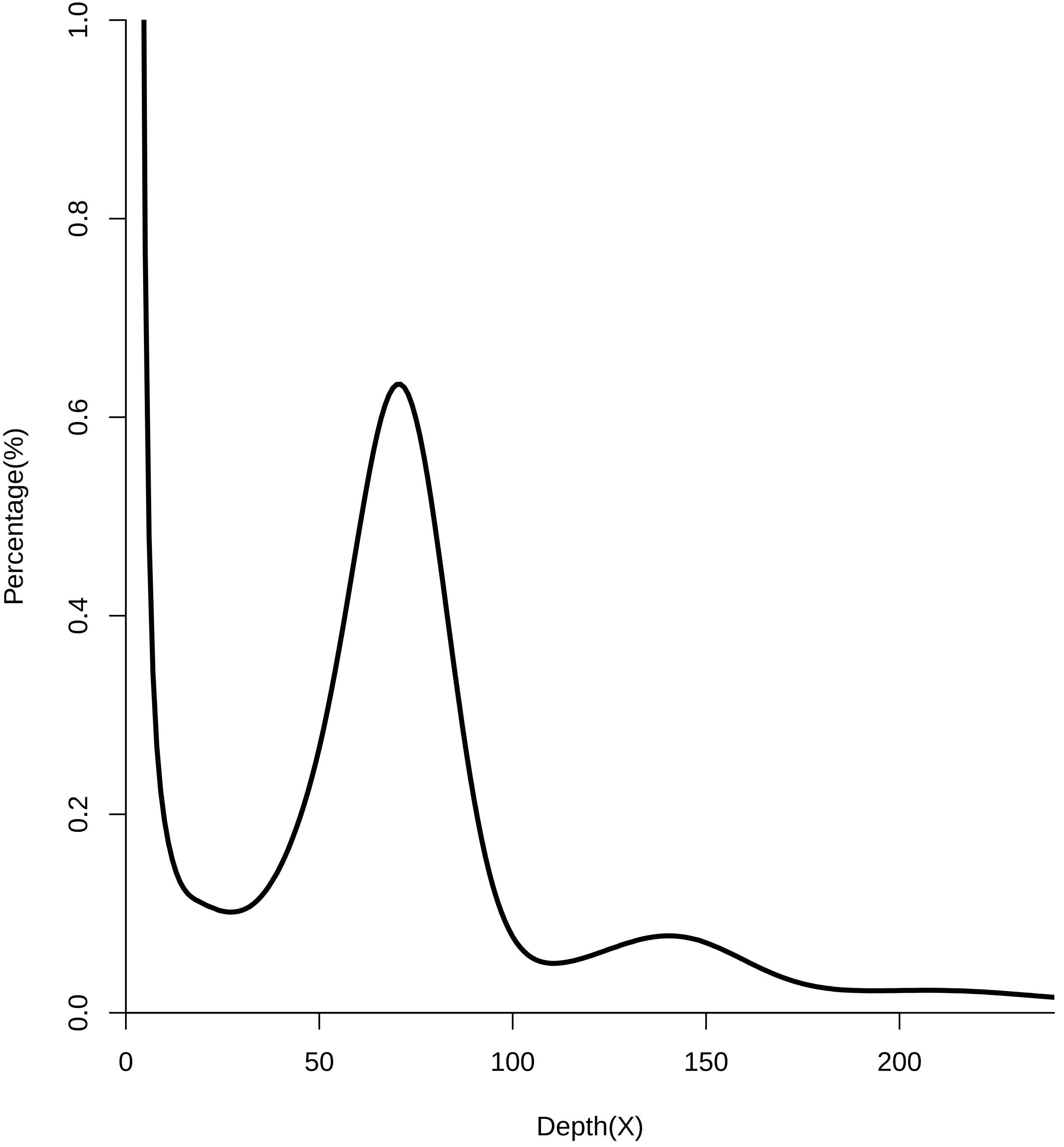

 Click here to download Figure Fig.3.pdf

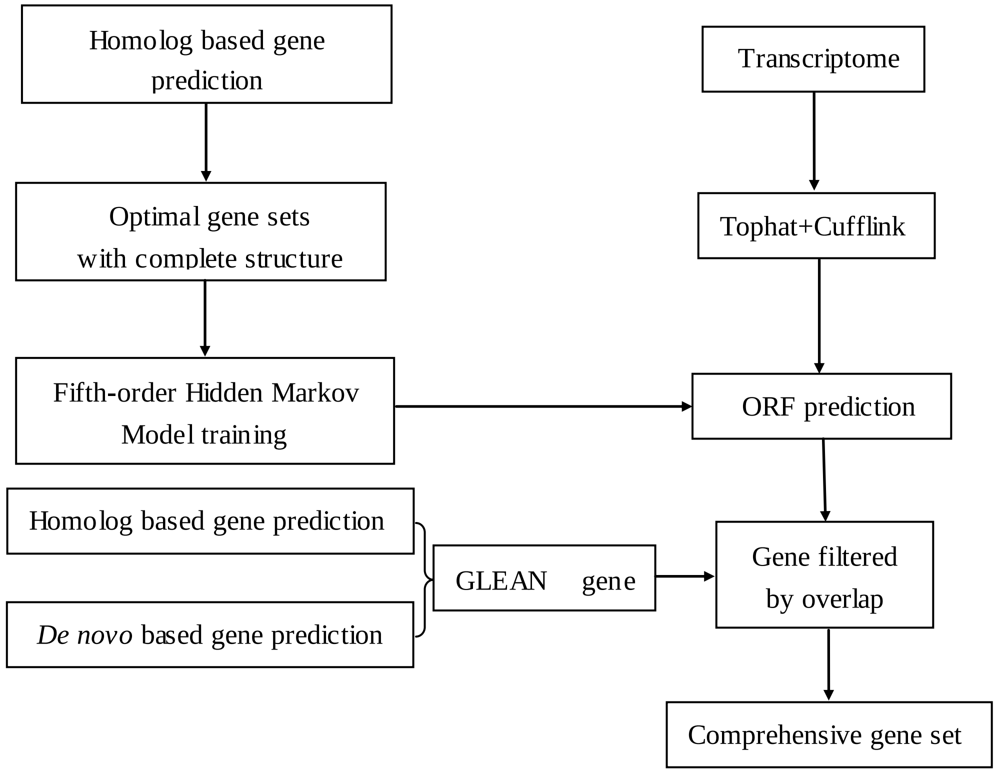

Personal Cover
Click here to download Personal Cover Cover Letter-yang.docx

Cover Letter

Dear editor

We would like to submit our manuscript entitle "The genome draft of coconut (*Cocos nucifera*)" for consideration of publication in Gigascience as a data note.

Coconut palm (*Cocos nucifera*, 2n = 32), belonging to the genus *Cocos* and the family Arecaceae (Palmaceae), is an important tropical fruit and woody oil crop. Meanwhile, the tropical crop is often used as an ornamental tree, which is a symbol for a tropical region. In order to accelerate molecular biology research and genetic breeding in *Cocos nucifera*, the whole genome of the tropical crop was sequenced and a total of 419.08 gigabases (Gb) of clean data was obtained, these clean reads were assembled with SOAPdenovo2. A total scaffold length of 2.2 Gb was generated, with a scaffold N50 of 418 Kb, which represents 91.67% of the estimated genome (2.4G). The coconut genome was predicted to harbor 28,039 protein-coding genes, which is slightly greater than *Phoenix dactylifera*(24,908). Genome annotation resultsrevealed that 72.75% of the coconut genome consists of transposable elements, among which long-terminal repeat elements (LTRs) make up the highest proportion (92.23%). The study provides a large amount of genomic information, facilitating future functional genomics and molecular breeding in *Cocos nucifera*. We believe that our study will be beneficial for the community, particularly who work on the study of genomic and molecular biology research in *Cocos nucifera*.

The authors also declare that the present work hasnot been submitted elsewhere for publication, in whole or in parts.Besides, all the listed authors have agreed and approved the contents ofthe manuscript.

Yaodong Yang
PHONE NUMBER: 0086-898-63330602
FAX NUMBER: 0086-898-63330673
EMAIL: yyang@catas.cn
POSTAL ADDRESS:
Coconuts Research Institute, Chinese Academy of Tropical Agricultural Sciences, Wenchan, Hainan 571339, P.R.China
