# Supplementary material for: The genome draft of coconut (Cocos nucifera)
Source: Gigascience. 2017 Oct 5;6(11):1–11. doi: 10.1093/gigascience/gix095 (PMC5714197; doi:10.1093/gigascience/gix095)
Supplement: GIGA-D-17-00038_Revision-1.pdf [file gix095_giga-d-17-00038_revision-1.pdf]

GigaScience
The genome draft of the Coconut (Cocos nucifera)
--Manuscript Draft--

**Manuscript Number:**	GIGA-D-17-00038R1
**Full Title:**	The genome draft of the Coconut (Cocos nucifera)
**Article Type:**	Data Note

Funding Information:

International Science and Technology Cooperation projects of Hainan Province (No. KJHZ2014-24)	Dr Yaodong Yang
Hainan Natural Science Foundation (313058)	Dr Wei Xia
the fundamental Scientific Research Funds for Chinese Academy of Tropical Agriculture Sciences (1630032012044)	Dr Yaodong Yang
the fundamental Scientific Research Funds for Chinese Academy of Tropical Agriculture Sciences (1630052014002)	Dr Yaodong Yang
the fundamental Scientific Research Funds for Chinese Academy of Tropical Agriculture Sciences (1630052015050)	Dr Yong Xiao
The major Technology Project of Hainan (ZDZX2013023-1)	Dr Ming Peng
the fundamental Scientific Research Funds for Chinese Academy of Tropical Agriculture Sciences (1630152017019)	Dr Yaodong Yang
the fundamental Scientific Research Funds for Chinese Academy of Tropical Agriculture Sciences (1630152016006)	Dr Yong Xiao
Central Public-interest Scientific Institution Basal Research Fund for Innovative Research Team Program of CATAS (17CXTD-28)	Dr Yaodong Yang

Abstract:

Background

Coconut palm (Cocos nucifera, 2n = 32), a member of genus Cocos and family Arecaceae (Palmaceae), is an important tropical fruit and oil crop. Currently, coconut palm is cultivated in 93 countries, including Central and South America, East and West Africa, Southeast Asia and the Pacific island, with a total growth area of more than 12 million hectares (www.fao.org/faostat/en/). Coconut palm is generally classified into two main categories: "Tall" (flowering 8-10 years after planting) and "Dwarf" (flowering 4-6 years after planting), based on morphological characteristics and breeding habits. This Palmae species has a long growth period before reproductive years which hinders conventional breeding progress. In spite of initial successes, improvements made by conventional breeding have been very slow. In the present study, we obtained de novo sequences of Cocos nucifera genome: a major genomic resource which could be used to facilitate molecular breeding in Cocos nucifera and accelerating the breeding process in this important crop.

Findings

A total of 419.67 gigabases (Gb) of raw reads were generated by the IlluminaHiSeq 2000 platform using a series of paired-end and mate-pair libraries, covering the predicted Cocos nucifera genome length (2.42Gb, variety "Hainan Tall") to an estimated 173.32× read depth. A total scaffold length of 2.20 Gb was generated (N50 = 418 Kb), representing 90.91% of the genome. The coconut genome was predicted to harbor 28,039 protein-coding genes, which is less than in Phoenix dactylifera (PDK30 variety: 28,889), Phoenix dactylifera (DPV01 variety: 41,660) and Elaeis guineensis (34,802). BUSCO evaluation demonstrated the obtained scaffold sequences covered 90.8% of the coconut genome, and that the genome annotation was 74.1% complete. Genome annotation results revealed that 72.75% of the coconut

	genome was consisted of transposable elements. of which long-terminal repeat retrotransposons elements (LTRs) accounted for the largest proportion (92.23%).<br>Comparative analysis of the antiporter gene family and ion channel gene families between C. nucifera and Arabidopsis thaliana indicated that significant gene expansion may occurred in coconut involving Na+/H+ antiporter, Carnitine/acylcarnitine translocase, Potassium-dependent sodium-calcium exchanger, and potassium channel genes.<br>Conclusions<br>Despite its agronomic importance, C. nucifera is still under-studied. In this report, we made an attempt to construct a draft genome of C. nucifera and provide an enormous amount of genomic information that will facilitate future functional genomics and molecular assisted breeding in this crop species.
**Corresponding Author:**	Yaodong Yang, Ph.D<br>Coconut Research Institute<br>Wenchang City, Hainan CHINA
**Corresponding Author Secondary Information:**	
**Corresponding Author's Institution:**	Coconut Research Institute
**Corresponding Author's Secondary Institution:**	
**First Author:**	Yong Xiao
**First Author Secondary Information:**	
**Order of Authors:**	Yong Xiao
	Pengwei Xu
	Haikuo Fan
	Luc Baudouin
	Wei Xia
	Stéphanie Bocs
	Junyang Xu
	Qiong Li
	Anping Guo
	Lixia Zhou
	Jing Li
	Yi Wu
	Zilong Ma
	Alix Armero
	Auguste Emmanuel Issali
	Na Liu
	Ming Peng
	Yaodong Yang
**Order of Authors Secondary Information:**	
**Response to Reviewers:**	Response to editor and reviewers<br><br>Dear editor and reviewers<br><br>Thank you very much for your crucial comments for our manuscript entitled “The genome draft of the Coconut (Cocos nucifera)" (GIGA-D-17-00038). We have made a thorough revision to the ms based on all of comments from editor and reviewers. Each

Powered by Editorial Manager® and ProduXion Manager® from Aries Systems Corporation

comments raised by the reviewers had been carefully answered in the response sheet. We hope the revised version can meet the requirement of “GigaScience”

Sincerely yours,

Yaodong Yang

PHONE NUMBER: 0086-898-63330602
FAX NUMBER: 0086-898-63330673
EMAIL: yyang@catas.cn
POSTAL ADDRESS:
Coconuts Research Institute, Chinese Academy of Tropical Agricultural Sciences,
496 Wenqing Av., Wenchang, Hainan 571339, P.R.China

Response to editor and reviewers,

Reviewer 1

1.Line 40: in 93 countries -> the introduction (line 70) say 89 countries

>>>Response: Thank you for your suggestion. We have re-checked the document reported by Batugal et al., 2005. The corresponding revision has been done in the Introduction part of the revised manuscript.

2.11 million ha ->the introduction (line 72) says 12 million ha

>>>Response: Thank you for your suggestion; we have re-checked the plant area of coconut in the website of Food and Agriculture Organization of the United Nations (http://www.fao.org/faostat/en/). The corresponding revision has been done in the Abstract part of the revised manuscript.

3.Hinders progress in genetic breeding. Do you mean ‘marker assisted breeding’ or ‘genomic assisted breeding’?

>>>Response: Thank you for your suggestion; we meant to say ‘conventional breeding’. Revisions have been made in the Abstract part of the revised manuscript to make our opinions clearer.

4.Genetic improvement is slow. Do you mean trait improvement with marker or genetic assisted

>>>Response: We meant to say the improvement made by ‘conventional breeding’ is slow. The corresponding revision has been done in the revised manuscript.

5.Line 48: The coverage does not add up. 714.67 Gb on a 2.42 Gb genome is 295× coverage. In any case, only the coverage of the cleaned reads should be shown (177×)`

>>>Response: Thank you for your suggestion; in revised manuscript, only the cleaned reads were used for the coverage depth analysis and the coverage is173.32× read depth.

6.Line54: Do you mean 41,166 genes

>>>Response: Thank you for your suggestion; we have re-checked the annotated gene number for datepalm based on the document reported by Al-Mssallem et al., 2013 and 41 660 genes were annotated. The corresponding revisions have been made in the Abstract part of revised manuscript.

7.Line60: space missing between facilitating and future

>>>Response: Thank you for your suggestion, a space has been added between facilitating and future.

Powered by Editorial Manager® and ProduXion Manager® from Aries Systems Corporation

8.Line 61: should be ‘molecular assisted breeding’

>>>Response: Thank you for your suggestion, corresponding revisions have been done in the Abstract part of revised version.

9.Line 78: ‘…wide range to environment…’ -> unclear, should be explained. Also ‘environment’

>>>Response: Some sentences have been added to the revised manuscript for explaining ‘…wide range to environment…’ in Line 240– Line 242|Page 3.

10.Line78: ‘…especially for high tolerance to high salt density.’ , please clarify

>>>Response: Coconut palm can disseminate through ocean currents: floating nuts sprout and grow naturally upon washing up on beaches. The ability to adapt to a high salt environment is closely related to this dissemination feature and to these natural growth conditions. Corresponding revision has been done in Line 243– Line 244|Page 3 of revised manuscript.

11.Line 80: ‘…making it possible to understand its adaptation to high salinity.’ You do not investigate this, you should change the statement to something milder such as: ‘This study forms the basis for future research investigating the coconuts tolerance to salt stress’

>>>Response: Thank you for your suggestion, We also present the genome sequence of HAT coconut and added an analysis of the antiporter and ion channel gene families, relevant to salinity tolerance, into the revised version. Corresponding revision had been added into in Line 237– Line 238|Page 3.

12.Line 82: provide references. The way this sentence reads at the moment, make it seem like you are also reporting those genome sequence.

>>>Response: The corresponding references have been added into Line 423|Page 4 of revised manuscript.

13.Line 92: space between ‘Illumina’, ‘Hiseq2000’ and ‘sequencer’

>>>Response: Two spaces had been added into between Illumina, Hiseq2000 and sequencer in Line 436|Page 4 of revised manuscript.

14.Line129: The data shows that you have higher coverage and a longer N50, it does not show that the assembly is of better quality.

>>>Response: Thank you for your suggestion, the sentence has been replace by other sentence: “The comparative results of the BUSCO estimation in coconut and in the four other palm genome sequences indicates that the smallest fraction of missing genes as predicted by BUSCO was found in the coconut genome assmebly”, in Line 724 – Line 726|Page 6 of revised version.

15.Line 131: ‘tissues’, not ‘issues’

>>>Response: Thank you for your suggestion, corresponding revisions has been done in the revised version.

16.Line134: table 4 and 5 are mixed up

>>>Response: We repeatedly checked Table 4 and 5. Corresponding revisions has been done in revised manuscript.

17.Line 165: BLAST not BLSAT

>>>Response: Thank you for your suggestion, ‘BLSAT’ had been modified in revised manuscript.

Powered by Editorial Manager® and ProduXion Manager® from Aries Systems Corporation

18.Line 175 (and others): keep a space between numbers and units, consistently.

>>>Response: we re-checked all numbers and units throughout the manuscript. All needed spaces have been added between numbers and units.

19.Line195: Change start of sentence (e.g. 'After the above described steps…')

>>> Response: Thank you for your suggestion, corresponding revision has been done in Line 970|Page 8 of the revised manuscript.

20.Line 196: should read: 'than the predicted gene markers..'

>>>Response: Thank you for your suggestion, corresponding revision has been done in Line 971 | Page 8 of revised version.

21.Line203: space between 'by' and 'sequence'

>>>Response: Thank you for your suggestion, a space had been added between by and sequence

22.Line211: after ref 38, just one dot

>>>Response: Thank you for your suggestion, the ref 38 and dot has been deleted in revised version.

23.Line 219: remove space between 'mapping' and ','

>>>Response: Thank you for your suggestion, the space has been deleted between 'mapping' and ','.

24.References: need a lot of editing to uniform

>>>Response: All references of the manuscript have been reviewed and edited based on the author guideline of "Gigascience" in the revised manuscript.

25.Tables: Headers are unclear and many abbreviations within tables are not explained

>>>Response: Thank you for your suggestion, revisions have been done for the table headers. Meanwhile, the abbreviations have been explained and replaced with corresponding full name.

26.What is the difference between Table 4 and Table 7? Both show BUSCO assessments of palm species. Clarify both in tables and in the text.

>>>Response: Thank you for your suggestion, Table 7 has been changed into Table 6 in the revised version. Table 4 referred to the comparative analysis of the assembled genome sequences for four palm species using BUSCO software, while Table 6 referred to the comparative analysis of the predicted gene from the four palm species using BUSCO software. Revisions have been done to make Table 4 and Table 6 legends more clearly in "Table" part of revised version.

27.Figure legend: Figure 1 does not contain any morphological characteristics; they are photographs of coconut plants.

>>>Response: Figure 1 had been substantially revised in the revised version.

Reviewer 2

1.My only major concern about the manuscript is that the written style is not ready for publication. There are many type and grammatical mistakes all over the main text, figure captions and table legends. The manuscript needs some extensive copy editing to be published.

>>>Response: Thank you for your suggestion, the manuscript has been reviewed and edited throughout the manuscript by the native experts (Annaliese Mason, Baudouin Luc and Amjad Iqbal).

Reviewer 3

1.Homologous gene families using a larger set of genomes would allow a gain-/loss analysis (check the Zostera (seagrass) genome paper Figure 1a for a recent example), some venn diagrams based on this showing how many gene are shared with close relative (e.g. Elaeis), other monocots (e.g. rice) and dicots (e.g. Arabidopsis) could also be generated based on this (e.g. orchid genome paper figure 1a). Asynteny/collinearity analyisis is usually included, often combined with a Ks analysis (see the orchid genome paper Figure 2, Zostera genome paper Figure 2).

>>>Response: Thank you for your suggestion, we added venn diagrams between different species and analyzed the divergence time between different species into Line 990| Page 8 - Line 1270 | Page 10 of the revised version. Meanwhile, we identified and characterized antiporter and ion channel gene family in Line 1271 | Page 10 – Line 1578 | Page 11 of revised manuscript.

2.No case study is included, I feel there should be at least one (though as the paper is submitted as a data note the journal might not require one). The authors are the first ones to have a glimpse at the genome of this species. I would make sense to check a few relevant gene families (coconut are clearly very different from seeds of other monocots, so seed related gene families would be likely candidates for a more in depth study)

>>>Response: Thank you for your suggestion. It is known that coconut palm can disseminate through ocean currents: floating nuts sprout and grow naturally upon washing up on beaches. The ability to adapt to a high salt environment is closely related to this dissemination feature and to these natural growth conditions. In the revised manuscript, we identified antiporter and ion channel genes in the genome of Cocos nucifera, some of which had been validated to be associated with salt stress in Arabidopsis. In the gene expansions analysis, some gene families showed significant expansion in compared to Arabidospsis, including Na+/H+ antiporter family, Carnitine/acylcarnitine translocase family, Potassium-dependent sodium antiporter, and potassium channel. The expansion of Na+/H+ antiporter family and Potassium-dependent sodium antiporter may be associated with coconut salt tolerance. The expansion of carnitine/acylcarnitine translocase family may be associated with the accumulation of fatty acid in coconut pulp. At last, the expansion of potassium channel may be associated with the accumulation of potassium ion in coconut water. Corresponding revision had been added into Line Line 1271 | Page 10 – Line 1578 | Page 11 of revised manuscript.

3.For non-bioinformaticians a supplemental website which offers a BLAST interface would certainly be welcome.

>>>Response: we have uploaded coconut genome raw data into Sequence Read Archive (SRA) of the National Center for Biotechnology Information. The assembled and annotated data were uploaded into GigaDB database. Meanwhile, the assembled and annotated data have been uploaded into pirate website for blast analysis and genome browse. However, currently, this website is not available for all people. The website will be available after further website improvement and paper publication

4.Line 128 -129: The N50 by itself is not a direct measure for the quality of the assembly. Avoid over-interpretation.

>>>Response: Thank you for your suggestion, the sentence has been replace by other sentence: "The comparative results of the BUSCO estimation in coconut and in the four other palm genome sequences indicates that the smallest fraction of missing genes as predicted by BUSCO was found in the coconut genome assmebly", in Line 724 – Line 726|Page 6 of revised version.

5.Line 54 and abstract: (DVP01, 4166) -> the number of genes for the date palm

Powered by Editorial Manager® and ProduXion Manager® from Aries Systems Corporation

genome is incorrect. In table 2 the authors report 41, 660 !

>>>Response: Thank you for your suggestion; we have re-checked the annotated gene number for datepalm based on the document reported by Al-Mssallem et al., 2013 and 41 660 genes were annotated. The corresponding revisions have been made in the Abstract part of revised manuscript.

6.Line 60: facilitating future: missing space Line 78-79

>>>Response: Thank you for your suggestion, a space has been added into between facilitating and future.

7.Line 78-79: For high tolerant to high salt density: revise grammar

>>>Response: Thank you for your suggestion, revisions have been done in Line 240–Line 242 | Page 3 of revised manuscript

8.Line 80: …present Hainan Tall… -> …present the Hainan Tall

>>>Response: Thank you for your suggestion, the sentence has been rewrite and the usage of the phrase “Hainan Tall” has been carefully checked throughout the revised manuscript.

9.Line 82: …about genome

>>>Response: Revisions have been done in revised manuscript.

10.Line 88 (and other places): …pair end… -> …paired end…

>>>Response: Thank you for your suggestion, all ‘pair end’ has been modified into ‘paired end’ throughout the revised manuscript.

11.Line 96: …removed by using … -> …removed using…

>>>Response: Thank you for your suggestion, corresponding revision had been done in revised manuscript

12.Line 116: …SOAPdenovo2 map… -> …SOAPdenovo2 maps…

>>>Response: Thank you for your suggestion, corresponding revision had been done in Line 572 | Page 5 of revised manuscript.

13.Line 132: ...reported in previous Fan’s research …: incorrect grammar should be revised (as previously reported by Fan et al…).

>>>Response: Thank you for your suggestion, corresponding revision had been done in Line 598 | Page 5 in revised manuscript

14.Line 181: Previous Fan’s research: revise grammar

>>>Response: ‘Previous Fan’s research’ had been modified into ‘as previously reported by Fan et al.’ in Line 874 | Page 7 of revised manuscript

15.Line 192: … a diagrammic pipeline is showed…: revise grammar

>>>Response: Thank you for your suggestion, corresponding revisions had been done in Line 968|Page 8 revised version.

16.Line 199: …completely… -> complete

>>>Response: Thank you for your suggestion, corresponding revision has been done in Line 973 | Page 8 of revised manuscript.

Powered by Editorial Manager® and ProduXion Manager® from Aries Systems Corporation

	17.Line 203 -204: In sequence similarity step: revise<br><br>>>>Response: 'In sequence similarity step' has been modified into 'Firstly' in Line 979|Page 8 of the revised manuscript.<br><br>18.Line 232-233: Font is suddenly somewhat bigger<br><br>>>>Response: Thank you for your suggestion, corresponding revision have been done in "Funding" part of revised manuscript.
**Additional Information:**	
**Question**	**Response**
Are you submitting this manuscript to a special series or article collection?	No
**Experimental design and statistics**<br><br>Full details of the experimental design and statistical methods used should be given in the Methods section, as detailed in our Minimum Standards Reporting Checklist. Information essential to interpreting the data presented should be made available in the figure legends.<br><br>Have you included all the information requested in your manuscript?	Yes
**Resources**<br><br>A description of all resources used, including antibodies, cell lines, animals and software tools, with enough information to allow them to be uniquely identified, should be included in the Methods section. Authors are strongly encouraged to cite Research Resource Identifiers (RRIDs) for antibodies, model organisms and tools, where possible.<br><br>Have you included the information requested as detailed in our Minimum Standards Reporting Checklist?	Yes
**Availability of data and materials**<br><br>All datasets and code on which the conclusions of the paper rely must be either included in your submission or deposited in publicly available repositories (where available and ethically appropriate), referencing such data using a unique identifier in the references and in the "Availability of Data and Materials" section of your manuscript.	Yes

Have you have met the above requirement as detailed in our Minimum Standards Reporting Checklist?	

Powered by Editorial Manager® and ProduXion Manager® from Aries Systems Corporation

Manuscript
Click here to download Manuscript manuscript revision20170720-with revisionsmode.doc

The genome draft of Coconut (*Cocos nucifera*)

Yong Xiao[1]*, Pengwei Xu[3]*, Haikuo Fan[1]*, Luc Baudouin[4]*, Wei Xia[1]*, Stéphanie Bocs[4]*, Junyang Xu[3], Qiong Li[2], Anping Guo[2], Lixia Zhou[1], Jing Li[1], Yi Wu[1], Zilong Ma[2], Alix Armero[4], Auguste Emmanuel Issali[5], Na Liu[3&], Ming Peng[2&], Yaodong Yang[1&]

[1]Hainan Key Laboratory of Tropical Oil Crops Biology/Coconut Research Institute, Chinese Academy of Tropical Agricultural Sciences, Wenchang, Hainan 571339, P.R.China

[2]Institute of Tropical Bioscience and Biotechnology, Chinese Academy of Tropical Agricultural Science, Haikou, Hainan 571101, P. R. China

[3]BGI-Shenzhen, Shenzhen 518083, China

[4]French Agriculture Research Centre for International for International Development (CIRAD), UMR AGAP, F-34398, Montpellier France

[5]Station Cocotier Marc Delorme, Centre National De RechercheAgronomique (CNRA) 07 B.P. 13, Port Bouet,Côte d'Ivoire

*The authors have equal contribution to the manuscript

[&]Corresponding author

Yong Xiao: xiaoyong1980@catas.cn

~~Wei~~ Pengwei Xu: xupengwei@genomics.cn

Haikuo Fan: vanheco@163.com

Luc Baudouin ~~Luc~~: luc.baudouin@cirad.fr

Wei Xia: saizjxiawei@hainu.edu.cn

~~Bocs~~Stéphanie Bocs: stephanie.sidibe-bocs@cirad.fr

Junyang Xu: xujy@genomics.cn

Qiong Li: liqiong4416@126.com

Anping Guo: gap211@126.com

Lixia Zhou: glzz_2009@163.com

Jing Li: lijing002x@catas.cn

Yi Wu: wuyi-scuta@163.com

Zilong Ma: mzl900@163.com

Formatted: Numbering: Continuous

Field Code Changed

Field Code Changed

Formatted: Font: Times New Roman, 9 pt

Formatted: Font: Times New Roman

ArmeroAlix Armero: alix.armero_villanueva@cirad.fr

Auguste Emmanuel Issali: issaliemma@yahoo.com

Na Liu: naliu@genomics.cn

Ming Peng: mmpeng_2000@yahoo.com

Yaodong Yang: yyang@catas.cn

Background

Coconut palm (*Cocos*_*nucifera*, 2n = 32), a member of genus *Cocos*_and of the family Arecaceae (Palmaceae), is an important tropical fruit and oil crop. Currently, coconut palm is cultivated in 93 countries, including Central and South America, East and West Africa, Southeast Asia and the Pacific island, with a total growth area of more than 1212 million hectares (www.fao.org/faostat/en/). Coconut palm is generally classified into two main categories: "Tall" (flowering 8-10 years after planting) and "Dwarf" (flowering 4-6 years after planting), based on the morphological characteristics and breeding habits. This palmae Palmae species needs has a long growth time period before entre into reproductive years which hinders the progress for conventional breeding progress. In spite of initial successes, the improvements made by conventional improvement breeding have beenis very slow. In the present study, we obtained *de novo* sequences of *Cocos nucifera* genome:, which will a majorwith its provide enormousa large amount of genomic informationresource which could be used forto facilitate the further molecular assisted breeding and accelerate the breeding process ofin *Cocos nucifera* ,and accelerating the breeding process in this important crop.genetic improvement is very slow.In Tthe present study, we obtained thewas performed to de novo sequence the genome of *Cocos nucifera*genome, which will provide a large amount of genomic information for molecular assisted breeding and accelerate the breeding process of *Cocos nucifera*.

Findings

A total of 419.67419.67 gigabases (Gb) of raw readsreads was were generated by the Illumina HiSeq 2000 platform using differenta series of combinations of paired- end and mate-pair librariesusing different combinations of paired end and mate pair libraries, comprising which covering approximately 173.32× depth of the173.32× depthofthe predictedestimated *Cocos nucifera* genome length*Cocos nucifera* genome (2.42Gb, variety "the Hainan Tall") to an estimated 173.32× read depth. A total scaffold length of 2.20 Gb was generated , with a (scaffold N50 of = 418 Kb), , whichand

Formatted: Font: No underline, Font color: Auto, (Intl) SimSun

Formatted: Font: Times New Roman, 9 pt

Formatted: Font: Times New Roman

representings 90.91% of the estimated genome (2.42Gb),., while T the BUSCO evaluation demonstrated the obtained scaffold sequences reached 90.8% completeness of coconut genome reached (90.8%). The coconut genome was predicted to harbor 28,039 protein-coding genes, which is less than in *Phoenix dactylifera* (PDK30 variety:, 28,889), *Phoenix dactylifera* (DPV01 variety:, 41,660) and *Elaeis guineensis* (34,802). BUSCO evaluation demonstrated the obtained scaffold sequences covered 90.8% of the coconut genome, and that The completeness level forthe annotation completeness genome annotation was also evaluatedestimated by BUSCO which showed a level of that was, reached to 74.1% completeness. Genome annotation results revealed that 72.75% of the coconut genome was consisteds of transposable elements., among inofOf which the class of long-terminal repeat retrotransposons elements (LTRs) make upaccountsed for the largest proportion (92.23%). Comparative analysis of the antiporter gene family and ion channel gene familyies between *C. nucifera* and *Arabidopsis thaliana* indicated thatasuggested significant gene expansion may happenedoccurred in the former genomecoconut, involving in Na+/H+ antiporter, Carnitine/acylcarnitine translocase, Potassium-dependent sodium-calcium exchanger, and potassium channel genes.

Conclusions

Despite its agronomic importance, *Cocos C. nucifera* is still under-studied. In the currentthis report, we made an attempt to construct a draft the genome of *Cocos. nucifera*, whichand provideds an enormousa large amount of genomic information that will facilitate future functional genomics and molecular assisted breeding in Cocos nuciferathis crop species.

In the current report, we made an attempt to draft the We report a genome draft of *Cocosnucifera*, which.Thisstudy providesa large amount of genomic information that will, facilitateingfuture functional genomics and molecular assisted breeding in*Cocosnucifera*.

Keywords: Coconut, .Ppalm, genome, Assembly, Annotation

Data description

Background

Coconut palm (*Cocos nucifera*, 2n = 32), the only species of fromin genus *Cocos* of andand belongs toin the family Arecaceae, is a tropical oil crop and is widely cultivated in tropical regions due to its extensive application in agriculture and industry. The Coconut palmtropical species was is thought to

Formatted: No underline, Font color: Auto

Formatted: Font: Not Italic, No underline, Font color: Auto

Formatted: Font: Not Italic, No underline, Font color: Auto

Formatted: Font: Times New Roman, 9 pt

Formatted: Font: Times New Roman

be originated from the sSouthewest and western Western pacific Pacific region (including the Malay Peninsula and archipelagoArchipelago, New Guinea, and the Bismarck Archipelago) and the southwest Pacific. At Ppresently, the this tropical tree crop had hasis been distributed across 93 tropical countries [1], including Central and South American, East and West African, Southeast Asia and the pacific Pacific islandsIslands, and is grownaccounts for over 12 million hectares of land (www.fao.org/faostat/en/).

In China, the coconut palm grows in the subtropical regions - Hainan and Yunnan provinces - as an economical and ornamental plant. In the province of Hainan, cCoconut palm is cultivated over forover an area of approximately 43,000 hectares in Hainan, withand, out of which the "Hainan Tall" (HAT) variety covereding approximately 36,000 hectares – is made upcovered by the coconut variety,the "theHainan Tall" (HAT) [2]. The Hainan TallHAT coconut are needs eight to ten years to entrerites reproductive stage andslow to mature (flowering 8-10 years after planting), has can grow to a height of about 20-30 meters, and have with a medium to large sized nut size. The Hainan Tall coconut Though thise HAT cultivar of coconut is highly tolerant to salt and drought stress, whilebut yet sensitive to temperatures below 10 °C. It is known that under natural conditions, cCoconut palm can can be disseminated through ocean currents: floating on the sea and the nuts that sprouts and grows naturally upon washing up onwhen reach the beach in natural conditiones. The ability ofto adapting to a high salt environment is closely related withto this dissemination feature and to these natural growth environmentHence, this tropical species gradually adapted to high salt environment during a long evolutionconditionsary process. The morphological characteristics of the Hainan TallHAT cultivar are givenshowedn –in Figure 1. Here, Besides, wWwe also present the genome sequence of Hainan TallHATthe Hainan Tall coconut and thean analysis forof the antiporter and ion channel gene familyfamilies, relevant to salinity tolerance, which will forms the basis for future research investigating the coconuts tolerance to salt stress. Moreover, Since theAs draft genome sequences of its coconut relative species,s (e.g.such as *Elaeis guineensis*[3] [3] and *Phoenix dactylifera* [4, 5], [4, 5]) have previously beenwere also reported. , we also performed TheAa comparative analysis has beenwas performed between coconut and itsthese relative species for the characters of the genome assembly and annotation characteristics results of coconut and its relative species in the study.

Formatted: Font: Times New Roman, 9 pt

Formatted: Font: Times New Roman

Data description

Sample collection and sequence sequencing strategy

The Ggenomic DNA was extracted from the spear leaf of the variety a“the the Hainan Tall” coconut (*Cocos nucifera* L. Taxonomy ID: 13894; 19033’3’’ 19°33’3”N, 110°047’25”’’ E) individual selected from the coconut garden of the the Coconut Research Institute (Wenchang, Hainan province, China) by using the CTAB extraction method [6]. Subsequently, four pairedpaired-end (PE) libraries with insert sizes asof 170 bp, 500 bp, 450 bp and 800 bp and five Matemate-pair (MP) libraries with insert sizes asof 2 Kb, 5 Kb, 10 Kb, 20 Kb and 40 Kb were constructed using the standard procedure provided by Illumina (San Diego, USA). After library preparation and quality control of the DNA samples, template DNA fragments were hybridized to the surface of the flow cells on an Illumina HiSeq2000 sequencer and, amplified to form clusters, and then sequenced by following the standard Illumina manual. Finally, we generated 714.67 Gb of raw reads from all constructed libraries., The raw outputs for sequenced outputs for each sequenced library are summarized in Table 1. Before assembly, We filtered the raw reads were pretreated withbyusing the following stringent filtering processes throughvia the SOAPfilter (v2.2) [7] software: (1) removedFiltered reads with 25% low-quality bases (quality scores ⩽ 7).; (2) Rremoved reads with N bases more than 1%.; (3) Ddiscarded reads with adapter contamination and/or PCR duplicates.; (4) removedFiltered reads with undersized insert sizes. Finally, 419.08 Gb (estimated 173.17× read depth) of high-quality sequences were obtained for genome assembly.

reads with low quality (base quality less than 7 with percent higher than 25% or N percent higher than 1%), small insert size,PCR duplication or adapter contamination were removed using SOAPfilter, a software applicationin the SOAPdenovo package[7]. After filtering, 419.08 Gb (173.17× depth) high-quality sequences were obtained forgenome assembly.

De novo assembly of short reads of *Cocos nucifera*

We used 209.38 Gb209.38Gb clean reads of the short-insert libraries (excludeing theinsert size 450bp library), excludinge the insert size of 450bp libraryin order to estimate the coconut genome size by k-mer frequency distribution analysis [7]. The genome size (G) of *Cocos nucifera* could be estimated by the following formula:

$$G = N \times (L - K + 1) / K\_depth$$

Formatted: No underline, Font color: Auto, Highlight

Formatted: Font: 10 pt

Formatted: Font: Times New Roman, 9 pt

Formatted: Font: Times New Roman

~~G = N × (L − K + 1)/K_depth~~

where N represents the total of number of reads, L represents the read length, K represents the k-mer value used in the analysis and K_depth refers to the main peak in the k-mer distribution curve. In our calculations, N was 2,049,520,223, L was 100 and K_depth was 71 for K=17. As a result, ~~therefore,~~ *Cocos nucifera* genome was estimated to be 2.42 gigabases (Gb). K-mer size distribution analysis (Figure 2) indicated that *Cocos nucifera* was a diploid species with low ~~heterozygous~~ heterozygosity and a high proportion of repetitive sequences.

We then assembled the *Cocos nucifera* genome ~~by~~ using the software SOAPdenovo2 in three steps: contig construction, scaffold construction and gap filling. In the contig construction step: the SOAPdenovo2 ~~set~~was run with the parameters 'pregraph -K 63 -R -d 1' ~~was employed~~ to construct de Bruijn graphs from paired-end libraries with ~~an~~ insert sizes ~~range~~ranging from 170 to 800 bp~~.~~ ; ~~Then t~~The k-mers from the de Bruijn graphs were then used to form contiguous sequences (contigs) with the parameters 'contig ~~–~~R' by clipping tips, merging bubbles and removing ~~the~~ low coverage links. In the scaffold construction step: the orders of the contigs were determined by using paired-end and mate-pair information with parameters 'map -k 43' and 'scaff -F -u'. In more detail~~more detail~~, SOAPdenovo2~~,~~ ~~maps~~ maps the reads from ~~paired~~paired-end and mate pair libraries to contigs based on a hash table (keys are unique k-mers on contigs; values are positions). In ~~this~~ such cases, two contigs are considered to be linked if the bridging of the contigs are supported by five paired-end read pairs or three mate-pair read pairs. In the gap filling step: ~~the bridging of the contigs are supported byfive paired-end read pairs or three mate-pair read pairs. In the gap filling step,~~ ~~:~~gaps within scaffolds were filled by utilizing KGF [7] (V1.06) and GapCloser software (v1.12-r6) [7] with paired-end libraries ~~with~~ (having an insert size from 170 to 800 bp in cases, where one end could be mapped to one contig and the other end extended into a gap). To ~~achieve~~ optimize~~al~~ the ~~–~~assembled sequence~~y result~~, Rabbit (a Poisson-based ~~K~~k-mer model software, see the URL in the "availability of supporting data" section~~path: availability of supporting data~~~~software, path: availability of supporting data~~) was used to ~~determine repeat sequences, segmental duplications or divergent haplotypes on the assembly. After~~ remov~~ale~~ ~~of~~ the redundant sequences~~,~~ ~~a~~A final ~~total~~ ~~scaffold~~ length of 2.20 Gb for the scaffolds was obtained and used for ~~continued~~further ~~generated~~analysis~~,~~ ~~which~~ accountings

Formatted: Font: Times New Roman, 9 pt

Formatted: Font: Times New Roman

forcomprising 90.91% of the predicted genome size (Table 2), which wasand larger than the African oil palm and dateother palm genomes (Table 2)species in palmacae. Meanwhile, the N50 of the the obtained contigs N50 was 72.64 Kb and the scaffold N50 was 418.06 Kb for the scaffolds, which have excludeding while the length of scaffolds less than 100 bp were excluded. The Ccomparison of N50 values for the s assembled y N50sof coconut genome and for with four previously published palm genomes *Elaeis guineensis* [3], *Elaeis oleifera* [3], *Phoenix dactylifera* (PDK30) [4] and *Phoenix dactylifera*(DPV01) [5] wereis listed inwere listed in Table 2.

Genome evaluation

The 57,304 unigenes (transcript obtained from three different tissues, spear leaves, young leaves and fruit flesh) as previously reported by Fan et al.reported by Fan et al. [8] were aligned to the assembled genome of *Cocos nucifera* using BLAT [9] with default parameters. The alignment results predicted indicated that the assembled genome of *Cocos nucifera* covered 96.78% of all the expressed unigenes, suggesting a high level of coverage haves been reached for the assembled genome (Table 3).

We also evaluated the level of genome completeness offor the assembled sequences byy using BUSCOv2.0 [10], which quantitatively assesses genome completeness by using evolutionarily-informed expectations of gene content from near-universal single-copy orthologs selected from OrthoDBv9 (http://busco.ezlab.org/, plant set). BUSCO analysis showed that there are separate 90.8% and 3.4% of the 1,440 expected plant genes were identified as complete and fragmented genes respectively, respectively, while 5.8 % of genes were considered asto be missing in from the assembled coconut genome sequencey. The BUSCO results showed that our assembly was more complete than assembled data reported from three palm species. The Comparison Ccomparingative results of the BUSCO resultsestimation, with in coconut and in the the other four other palm genome sequenceses indicateds that the smallest fraction of missing genes as predicted by BUSCO genes happenedwas found in the coconut genome assmeblyassembly (Table 4).the BUSCO results with the other four palm genomes indicated the smallest missing of smallest BUSCO genesin coconut genome (Table 4).

Repeat annotation

We combined a homology - based annotation and *de novo* method to identify transposable elements (TEs) and the tandem repeats in the *Cocos nucifera* genome. In homology - based annotation step: TEs at DNA and protein levels were identified by searching against the Repbase library (version

Formatted: No underline, Font color: Auto, Not Highlight
Formatted: No underline, Font color: Auto, Not Highlight
Formatted: No underline, Font color: Auto, Not Highlight
Formatted: No underline, Font color: Auto, Not Highlight
Formatted: No underline, Font color: Auto, Not Highlight
Formatted: No underline, Font color: Auto, Not Highlight
Formatted: No underline, Font color: Auto, Not Highlight
Formatted: No underline, Font color: Auto, Not Highlight
Formatted: No underline, Font color: Auto, Not Highlight
Formatted: No underline, Font color: Auto, Not Highlight
Formatted: No underline, Font color: Auto, Not Highlight
Formatted: No underline, Font color: Auto, Not Highlight
Formatted: No underline, Font color: Auto, Not Highlight
Formatted: No underline, Font color: Auto, Not Highlight
Formatted: Font: Times New Roman, 9 pt
Formatted: Font: Times New Roman

20.04) [11] with RepeatMasker (v4.0.5) [12] and RepeatProteinMasker (v4.0.5) [12]. In the *de novo* step: *de novo* libraries were constructed based on the genome sequences using the *de novo* prediction program RepeatModeler (~~path:S~~see the URL in the “~~availability of supporting data~~availability of supporting data” section) and LTR_FINDER [13] by removing ~~contamination~~ contaminant and multi-copy genes. Subsequently, ~~Then the~~ novel transposable elements were identified and classified using RepeatMasker. T~~he t~~andem repeat sequences were identified by TRF (Tandem Repeat Finder) software [14] with the following parameters ‘Match = 2, Mismatch = 7, Delta = 7, PM = 80, PI = 10, Minscore = 50 and MaxPerid = 2000’. The total length of the tandem repeat sequences predicted by the software ~~is~~ was 151,229,585 bp, comprising 6.86% of the coconut genome. Finally, ~~a total of~~ 1.6 Gb of non-redundant repetitive elements were identified, accounting for 74.48% of the coconut genome~~, while~~. T~~t~~ransposable elements took up 72.75% ~~for~~of the total 1.6Gb of repetitive elements ~~and~~with the~~. The most predominant transposons were~~ long-terminal repeat retrotransposon (LTR) class ~~, which~~ account~~s~~ing for 92.23% of all TEs and 67.1% of the coconut genome (Table 5).

Gene prediction

~~We combined homology, *de novo*and transcript alignment to predict genes in *Cocosnucifera* genome.~~

We combined three strategies to predict genes in *Cocos nucifera* genome: homology - based, *de novo* and transcript alignment~~ to predict genes in *Cocos nucifera* genome~~. ~~For homology prediction:~~ For homology ~~prediction~~ based annotation: the protein sequences~~the proteinsequences of *Arabidopsis thaliana*[15],*Oryza sativa*[16],*Sorghum bicolor*[17]and *Zeamays*[18]~~ of *Arabidopsis thaliana* [15], *Oryza sativa*[16], *Sorghum bicolor* [17], *Zea mays* [18], *Elaeis guineensis*, and *Phoenix dactylifera* (DPV01)~~and *Elaeis guineensis* and *Phoenix dactylifera* (DPV01)~~ were downloaded from each corresponding sources (see “Availability of data sources”)~~from each corresponding sources (See “Availabilityof data sources”)~~. ~~The longest transcript was selected to represent the genes with among differnt alternative splicing variants. We aligned these homologous proteins to t~~The coconut genome was ~~blast~~aligned against these downloaded databases using TBLASTN[19] with parameter ‘-e 1e-5 -F -m 8’~~,~~ and ~~connected the~~ BLAAST ~~hit~~ results were processed~~to candidate gene loci~~ by solar (v0.9) with parameter ‘-aprot_2_genome2 -z’ to determine the candidate gene loci. Next, we extracted the genomic sequences of candidate gene loci along with ~~up and down stream~~ 1kb flanking sequences,~~,~~ and appl~~i~~ied~~ied~~ Genewise 2.2.0 [20] to define the intron - exon ~~boundary~~boundaries. The genes with pre-stop codon or frame-~~shifted~~ shifts were excluded ~~for~~ from further analysis.

Formatted: Font: Times New Roman, 9 pt

Formatted: Font: Times New Roman

For ~~De~~ de *novo* prediction: ~~We~~ we randomly selected 1000 ~~full~~ full-length genes (GeneWise score equal 100, intact structure: start codon, stop codon, perfect intron-exon boundary) from gene ~~sets~~ models predicted by ~~homology~~ homology-based methods to train the model parameters for AUGUSTUS2.5[21]. Two software programs, AUGUSTU2.5 and GENSCAN 1.0 [22], were used to do *de novo* prediction on the repeat-masked genome of *Cocos nucifera*. Genes with incomplete structure or protein coding length less than 150bp were filtered out.

Subsequently, ~~Then G~~genes from both homology-based and *de novo* methods were combined to ~~get~~ obtain non-redundant gene sets by using GLEAN [23] with the following parameters: minimum coding sequence length 150 bp and maximum intron length 50 kb. Genes were filtered with the same thresholds as were used for homology-based annotation.

For transcriptome-based prediction: RNA-seq data (SRR606452) as ~~as~~ previously reported by Fan et al. ~~as prvioiuslypreviously reported by Fan et al.~~[8] ~~were~~ was mapped onto the coconut genome to identify the splice junctions using the software TopHat (v2.1.1) [24]. ~~And then~~The software Cufflinks (v2.2.1) [25] was then used to assemble transcripts with the aligned reads. The coding potential of these transcripts was identified using a fifth-order Hidden Markov Model, which was estimated with the same gene sets used in AUGUSTUS training by train_GlimmerHMM, an application in the GlimmerHMM [26] package. The transcripts with intact open reading frames (ORFs) were ~~exacted~~ extracted and the longest ~~ORF~~ transcript was retrieved as ~~while~~a representative of a gene whiles multiple ~~isisoforms~~transcripts from~~located~~ ~~in~~ on ~~the~~a same locus.

~~At last~~Finally, we merged the GLEAN and the transcriptome result to form a comprehensive gene set using an in-house annotation pipeline with the ~~in~~ following steps: firstly, all-to-all BLASTP analysis of protein sequences w~~ere~~as performed between GLEAN results and transcript assemblies with an E-value cutoff of 1e-10. These transcript assemblies were added to the GLEAN result to form (untranslated region) UTRs or alternative spliceing products, depending on whether the coverage and identity of the alignment results reached 0.9 or not. If the transcript assemblies had no ~~blast~~BLAST hit with the GLEAN results, these transcript assemblies ~~would be~~were added to the final gene set as novel gene.~~in~~the~~following steps: firstly, all-to-all BLASTP analysis of protein sequences were performed between GLEAN result and transcript assemblies with an E-value cutoff 1e-10. These transcript assemblies were added to the GLEAN result to form (untranslated region) UTRs or alternative splice on whether coverage and identity of the~~

Formatted: Font: Times New Roman, 9 pt

Formatted: Font: Times New Roman

alignment results reached 0.9 or not. If the transcript assemblies had no blast hit with the GLEAN result, these transcript assemblies would be added to the final gene setas novel gene. The protocol for integrating GLEAN and Transcriptome transcriptome data is shown in Figure 3.

Gene evaluation

After the above described steps, we obtained a final gene set containedThe annotation results showedprocesses have identified a total of 28,039 protein-coding protein-codinggenes were obtained (Table 2), which is less than the predicted predicted gene numbers of *Phoenix dactylifera* (PDK30,28,889), *Phoenix dactylifera* (DPV01, 41,660) and *Elaeis guineensis* (34,802). Meanwhile,, thethrough the BUSCO evaluation showed the a separate ofthat ,.demonstrated74.1% and 11.2% of 1,440 expected plant genes were identified as complete and fragmented, and, with 14.7% of genes were considered missing in the gene sets. The BUSCO results showed that our gene prediction was more complete than that of *Phoenix dactylifera* (PDK30) and *Elaeis guineensis*, but less completely than that of *Phoenix dactylifera* (DPV01) (Table 6). ,This maybe due to the higher repetitive elements hinderence of the gene prediction of coconut genome by higher repetitive elements.

Gene Function

Gene function annotation was identified done by based on sequence similarity and domains conservation. In Firstly,the step of sequence alignment: we searched aligned the coconut protein coding genes were blastaligned againstagainst with the KEGG protein databases [27], SwissProt and TrEMBL [28] using BLASTP at a cut-off E-value threshold of 10^{-5}. Subsequently, the Then we use the best match of from the alignment was used to represent the gene function. We obtained 18,445 KEGG, 18,867 Swissprot and 24,882 Tremble annotated genes. In domains conservation step:Secondly, InterProScan 5.11-51.0 software [29] was employed to identify the motif and domain based onagainst the public databases Pfam [30], PRINTS [31], ProDom [32], SMART [33], PANTHER [34], TIGRFAM [35] and SUPERFAMILY [36]. This The gene function annotationrevealed demonstrateddemonstrated that 21,087 of the coconut proteins had conserved motifs and, 1,622 Gene Ontology (GO) terms were assigned to 15,705 coconut proteins from the corresponding InterPro entry [37]. In total, approximately 89.41% of these genes were functionally annotated using the above methods.

Gene Family Construction

Formatted: Font: Times New Roman, 9 pt

Formatted: Font: Times New Roman

Protein sequences of thirteen angiosperms, including *Elaeis guineensis*, *Phoenix dactylifera* (DPV01), *Sorghum bicolor*, *Prunus persica*, *Solanum tuberosum*, *Glycine max*, *Arabidopsis thaliana*, *Theobroma cacao*, *Vitis vinifera*, *Musa acuminata*, *Carica papaya*, *Populus trichocarpa* and, *Amborella trichopoda*, were download from each corresponding ftp siteProtein sequences of thirteen angiosperms, including *Elaeis guineensis*, *Phoenix dactylifera* (DPV01), *Sorghum bicolor*, *Prunus persica*, *Solanum tuberosum*, *Glycine max*, *Arabidopsis thaliana*, *Theobroma cacao*, *Vitis vinifera*, *Musa acuminata*, *Carica papaya*, *Populus trichocarpa*, *Amborella trichopoda*, were download from each corresponding ftp site (see "Availability of data sources"). For genes with alternative splicing variants, the longest transcripts wasere selected to represent the gene. The gene numbers of *Elaeis guineensis* and *Phoenix dactylifera* (DPV01) were greatly different from the research paper published in 2013[3, 5] (reference), because genes of these two species were re-predicted using the NCBI Prokaryotic Genome Annotation Pipeline which seemed to be more reasonable. Similarities between paired sequences were calculated using BLASTP with an E-value threshold of 1e-5. OrthoMCL [38] was used to identify gene family based on the similarities of the genes and a Markov Chain Clustering (MCL) with default parametersFor genes with alternative splicing variants, the longest transcript was selected to represent the gene. The gene numbers of *Elaeis guineensis* and *Phoenix dactylifera* (DPV01) were greatly different from the research paper published in 2013, because genes of these two species were re-predicted using NCBI Prokaryotic Genome Annotation Pipeline, which seemed to be more reasonable. Similarities between pair sequence were calculated using BLASTP with E-value threshold of 1e-5. OrthoMCL [38] was used to identify gene family based on the similarities of the genes and a Markov Chain Clustering (MCL) with default parameters. About 79.80-% of *Cocos nucifera* genes were assigned into 14,411 families, of which 282 families were only existinged in *Cocos nucifera* (coconut specific families) (Table 7). Figure 4 showseds the shared gene families offor orthologous genes. There are 544 orthologous families shared by five monocot species and 7706 orthologous families shared by all monocot and dicot species, suggesting 544 monocot unique functions shared by five monocot species and 7,706 ancestral functions in the most recent common ancestor of the angiosperms.of *Cocos nucifera* genes were assigned in 14,411 families, 282 families were only existing in *Cocos nucifera*(coconut specific families) (Table 7). Figure 4 shows shared gene families of orthologous genes. There are544orthologous families shared by five monocot species and 7706 orthologous families shared by all monocot and dicot species,suggesting544 monocot unique

Formatted: No underline, Font color: Auto, Highlight

Formatted: Font: Times New Roman, 9 pt

Formatted: Font: Times New Roman

~~functions shared by five monocot species and 7,706 ancestral functions in most recent common ancestor of angiosperms.~~

Phylogenetic analysis

We extracted 247 single copy orthologous genes derived from the gene family analysis step, and then aligned the protein sequences of each family with MUSCLE (v3.8.31) [39]. Next, the protein alignments were converted to corresponding coding sequences (CDS) using an in-house Perl script. These coding sequences of each single copy gene family were concatenated to form one super gene for each species. The nucleotides at position 2 (phase one site) and 3 (four degenerate site) of codon were extracted separately to construct the phylogenetic tree by PhyML3.0 [40] ~~with~~using a HKY85 substitution model and a gamma distribution across sites. The tree constructed by phase one sites was consistent with the tree constructed by four degenerate sites.

~~We extracted 247single copy orthologous genes from the gene family step, and then aligned the protein sequences of each family with MUSCLE (v3.8.31) [39]. Next, the protein alignments were converted to corresponding coding sequences (CDS) using an in-house Perl script. These coding sequences of each single copy family were concatenated to form one super gene for each species. The nucleotides at position 2 (phase one site) and 3 (four degenerate site) of codon were extracted separately to construct the phylogenetic tree by PhyML3.0 [40] with HKY85 substitution model and a gamma distribution across sites. The tree constructed by phase one sites was consistent with tree constructed by four degenerate sites.~~

Divergence time

The Bayesian relaxed molecular clock approach was used to estimate species divergence time using MCMCTREE in PAML~~The Bayesian relaxed molecular clock approach was used to estimate species divergence time using MCMCTREE in PAML~~ [41], based on the four-degenerate sites~~based on the four-degenerate sites~~ and the data set used in phylogenetic analysis, with previously published calibration times [42] (~~D~~divergence ~~between*Arabidopsis*~~between *Arabidopsis thaliana* and *Carica papaya* was 54-90 Mya, divergence between *Arabidopsis thaliana* and *Populus trichocarpa* was 100-120 Mya). The divergence time between coconut and oil palm is about 46.0 (25.4-83.3) million years ago (Figure 5), which is less than the divergence time between coconut and date palm.~~data set used in phylogenetic analysis, with previously published calibration times [42] (Divergence~~

Formatted: Font: Times New Roman, 9 pt

Formatted: Font: Times New Roman

between*Arabidopsis thaliana* and *Carica papaya* was54-90 Mya, divergence between *Arabidopsis thaliana* and*Populus trichocarpa* was 100-120 Mya).The divergence time between coconut and oil palm is about 46.0(25.4-83.3) million years ago (Figure 5), which is less than the divergence time between coconut and date palm.

Identification of antiporter genes in coconut genome

Antiporters isare a transmembrane proteins involveding in the exchange of two substances within and outsideopposite directions through the membrane. In Arabidopsis, the functions of Arabidopsis antiporter genes have been well characterized experimentally,, and this gene family werewas subdivided into thirteen different functional groups. Among them, three functional clusters involved in Na+/H+ antiporters, some of which were documented to be associated with salt tolerance [43, 44].

Antiporter is a transmembrane protein involving in exchange of two substances in opposite directionsthrough the membrane. In Arabidopsis, the functionsof Arabidopsis antiporter genes have been well characterized experimentally, and were subdivided into thirteendifferent functional groups. Among them, three functional clusters involved in Na+/H+ antiporter, some of which were documented to be associated with salt tolerance [43, 44].

The amino acid sequences of 70 *antiporter* genes of Arabidopsis were downloaded from the Arabidopsis Information Resource (TAIR) website (http://www.arabidopsis.org) wereand used as queries tofor BLASTP against the predicated protein databases ofin the *Cocos nucifera* genome atwith a cut-off e-valaue of 1e-10. A total of 126 *antiporter* genes were identified in coconut genome.The amino acid sequences of 70 *antiporter* genes of Arabidopsis downloaded from the Arabidopsis Information Resource (TATR) website (http://www.arabidopsis.org) were used as queries to BLASTP against the protein database of *Cocos nucifera* at a cut-off e-vlaue of 1e-10. A total of 126 *antiporter* genes were identified in coconut genome. With the help of theUsing local Hidden Markov Model-based HMMER (v3.0) searches and the Pfam database, seven antiporter genes were excluded forfrom further analysis because of the lack of conserved domain. The detailed information of the 119 antiporter genes wereis listed in Additional file 1.

local Hidden Markov Model-based HMMER (v3.0) searches and Pfam database,seven antiporter genes were excluded for further analysis because of lack of conserved domain. The detailed information of the 119 antiporter genes werelisted in Supplementary Table1.

In order to elucidate the evolutionary relationship and potential functions of the antiporters

Formatted: No underline, Font color: Auto, Not Highlight

Formatted: Font: Times New Roman, 9 pt

Formatted: Font: Times New Roman

identified in the study, we applied a combined phylogenetic analysis of *Arabidopsis* and *C. nucifera* antiporter proteins usingbyusing the neighbor joining method (Figure 6). Phylogenetic analysis showed that the 119 antiporter genes from *C. nucifera* can be subdivided into twelve groups. Meanwhile, and that almost all antiporter genes from *C. nucifera* can bewere clustered intotogether with the functional groups found in *Arabidopsis thaliana*.

In order to elucidate the evolutionary relationship and potential functions of the *EgMYBs* identified in the study, we applied a combined phylogenetic analysis of *Arabidopsis* and *C. nucifera*antiporter proteins using the neighbor joining method (Figure 6). Phylogenetic analysis showed that the 119 antiporter genes from *C. nucifera*can be subdivided into twelve groups. Meanwhile, almost all antiporter genes from *C. nucifera* can be clustered into the function groups found in *Arabidopsis thaliana*.

Phylogenetic analysis showed that the number of antiporter genes wereas equal between *Arabidopsis thaliana* and *C. nucifera**Elaeis guineensis* amongfor allmost groups except for G1 (one of three Na+/H+ antiporter family), G3 (Ccarnitine/acylcarnitine translocase family) and G12 (Ppotassium-dependent sodium-calcium exchanger). The In the three groups, the genes from *C. nucifera* are far more than these from *Arabidopsis thaliana*, for example, G1 group (one of three Na+/H+ antiporter familyies) only contained only one Arabidopsis antiporter gene and but 14 *C. nucifera* antiporters (1-At/14-Cn),. Phylogenetic analysis showed that the number of antiporter genes were equal between *Arabidopsis thaliana* and *Elaeis guineensis* among all groups except for G1 (one of three Na+/H+ antiporter family), G3 (Carnitine/acylcarnitine translocase family) and G12 (Potassium-dependent sodium-calcium exchanger). In the three groups, the genes from *C.nucifera*are far more than these from *Arabidopsis thaliana*, for example, G1 group (one of three Na+/H+ antiporter family) only contained one Arabidopsis antiporter gene, and but 14 *C. nucifera* antiporters (1/14), whereas G3 (Ccarnitine/acylcarnitine translocase family)G3 (Carnitine/acylcarnitine translocase family) contained (1-At/29-Cn), and G13 (PatassiumPotassium-dependent sodium-calcium exchanger)G3 (PotassiumPatassium-dependent sodium-calcium exchanger) contained (3-At/11-Cn). The These results indicated gene family expansion involving in the three functional groups. Na+/H+ antiporter family had been reported to be associated with salt stress. Hence, tThe expansion of the Na+/H+ antiporter gene family in coconut palm maybe associated with the high salt tolerance of coconut. Meanwhile, carnitine/acylcarnitine translocase is involved in fatty

Formatted: Font: Times New Roman, 9 pt

Formatted: Font: Times New Roman

acid transport cross the mitochondrial membranes. Hence, tThise gene family expansion of the gene family maybe associated with accumulation of fatty acid in coconut pulp. Moreover, coconut water containeds a high density of potassium ion, approximately 312 mg potassium ion per 100 g coconut water [45]. In theis study, the gene number of potassiumpatassium-dependent sodium-calcium exchangerswere also detected to be significant expansion. potassium-dependent sodium-calcium exchangers were also detected to be significantly increased comparinged withto Arabidopsisexpansion.

Identification of ion channel genes in coconut genome

A total of 67 ion channel genes were identified in the coconut genome (Additional file 2). The amino acid sequences of 67 *C. nucifera* and 60 *Arabidopsis* ion channel genes were used to demonstrateanalyze their evolutionary relationship (Figure 7). Almost all ion channel genes from *C. nucifera* can be clustered into the function groups found in *Arabidopsis thaliana*. The number of ion channel genes was equal between *Arabidopsis thaliana* and *Cocos nucifera* among allin most groups except for G5 (potassium channel). Many moreThe genes (21) from *C. nucifera* are far more than these (9) from *Arabidopsis thaliana* (9 genes) were present in group 5 (potassium channels), whichindicating gene family expansion are involveding in potassium channel. The gene family expansion maybe associated with accumulation of potassium ions in coconut water.

Conclusion

Cocos nucifera (2n = 32) is an important tropical crop, and is also used as an ornamental plant in the tropics. In the present study, we sequenced and *de novo* assembled the coconut genome. A total scaffold length of 2.2 Gb was generated, with a scaffold N50 of 418 Kb. The divergence time of *Cocos nucifera* and *Elaeis guineensis* is lessmore recent than that of *Cocos nucifera* and *Phoenix dactylifera*, suggesting thea closer relationship ofbetween *C. nucifera* and *E. guineensis* is more closerly. Comparative analysis of antiporter and ion channels between *C. nucifera* and *Arabidopsis thaliana* showed significant gene family expansions maybe involving Na+/H+ antiporters, carnitine/acylcarnitine translocases, potassium-dependent sodium-calcium exchangers, and potassium channels. The expansion of these gene families may be associated with adaptation to salt stress, accumulation of fatty acid in coconut pulp and potassium ions in coconut water.The function of expanded gene families in species evolution is always tend toalways related towith the environmental

Formatted: Font: Times New Roman, 9 pt

Formatted: Font: Times New Roman

adaption species adapt and the identified, the expand gene families ofexpansion happened in coconut palm may be associated with its' salty adaption and unique taste of coconut water.The divergence time of *Cocos nucifera* and *Elaeis guineensis* is less than *Cocos nucifera* and *Phoenix dactylifera*, suggesting the a closerelationship of between *C. nucifera* and *E. guineensis* is more closely.The function of expanded gene families is always related to the environment that a species adapt, therefore, expand gene families of coconut may associate with its' salty adaption and unique taste of coconut water. The data output of the coconut genome will provide a valuable resource and reference information for the development of high density molecular makers, construction of high density linkage maps, detection of QTL (quantitative trait loci), genome-wide association mapping, and molecular breeding. Comparative analysis of antiporter and ion channel between *C. nucifera* and *Arabidopsis thaliana* suggestedshowed significant gene expansion involving in Na+/H+ antiporter, Carnitine/acylcarnitine translocase, Patassium-dependent sodium-calcium exchanger, and potassium channel. The expansion of these gene families may be associated with coconut salt stress, accumulation of fatty acid in coconut pulp and potassium ion in coconut water.

Availability of supporting data

Supporting data are available in the GigaDB database, and the raw data were deposited in the SRA539146 with the project accession code PRJNA374600 for the *Cocos nucifera* genome. Previously published RNA-seq data used for transcriptome-based prediction is available from the under accession number SRR606452.

Availability of software

Rabbit:ftp://ftp.genomics.org.cn/pub/Plutellaxylostella/Rabbit_linux-2.6.18-194.blc.tar.gz

RepeatModeler: http://www.repeatmasker.org/RepeatModeler.html,version1.0.5

Solar: https://sourceforge.net/p/treesoft/code/HEAD/tree/branches/lh3/solar/

HMMER:http://www.ebi.ac.uk/Tools/hmmer

Availability of software

Rabbit:ftp://ftp.genomics.org.cn/pub/Plutellaxylostella/Rabbit_linux-2.6.18-194.blc.tar.gz

RepeatModeler: http://www.repeatmasker.org/RepeatModeler.html,version1.0.5

Solar: https://sourceforge.net/p/treesoft/code/HEAD/tree/branches/lh3/solar/

HMMER:http://www.ebi.ac.uk/Tools/hmmer

Formatted: Font: Italic, No underline, Font color: Auto

Formatted: Font: Italic, No underline, Font color: Auto

Formatted: Line spacing: 1.5 lines

Formatted: Font: Times New Roman, 9 pt

Formatted: Font: Times New Roman

Availability of other angiosperms data sources

Arabidopsis thaliana, *Oryza sativa*, *Sorghum bicolor*, *Zea mays*, *Sorghum bicolor*, *Solanum tuberosum*, *Prunus persica*, *Theobroma cacao*, *Vitis vinifera*, *Musa acuminata*, *Carica papaya*, *Populus trichocarpa*, *Amborella trichopoda*: https://phytozome.jgi.doe.gov/pz/portal.html (phytozomev9.1)

Elaeis guineensis: ftp://ftp.ncbi.nlm.nih.gov/genomes/all/GCF/000/442/705/GCF_000442705.1_EG5/

Phoenix dactylifera (DPV01):

ftp://ftp.ncbi.nlm.nih.gov/genomes/all/GCF/000/413/155/GCF_000413155.1_DPV01/

Phoenix dactylifera (PDK30):

http://qatar-weill.cornell.edu/research/datepalmGenome/download.html

~~**Availability of other angiosperms data sources**~~

~~*Arabidopsis thaliana*, *Oryza sativa*, *Sorghum bicolor*, *Zea mays*,*Sorghum bicolor*,*Solanum tuberosum*,*Prunus persica*,*Theobroma cacao*, *Vitis vinifera*, *Musa acuminata*, *Carica papaya*, *Populus trichocarpa*, *Amborella trichopoda*:https://phytozome.jgi.doe.gov/pz/portal.html (phytozomev9.1)~~

~~*Elaeis guineensis*: ftp://ftp.ncbi.nlm.nih.gov/genomes/all/GCF/000/442/705/GCF_000442705.1_EG5/~~

~~*Phoenix dactylifera* (DPV01):~~

~~ftp://ftp.ncbi.nlm.nih.gov/genomes/all/GCF/000/413/155/GCF_000413155.1_DPV01/~~

~~*Phoenix dactylifera* (PDK30):~~

~~http://qatar-weill.cornell.edu/research/datepalmGenome/download.html~~

Competing interests

The authors declare that they have no competing interests.

Funding

This study was supported by International Science and Technology Cooperation projects of Hainan Province (No. KJHZ2014-24), Hainan Natural Science Foundation (No. 313058), the major Technology Project of Hainan (No. ZDZX2013023-1), the fundamental Scientific Research Funds for Chinese Academy of Tropical Agriculture Sciences (CATAS-No. 1630032012044, 1630052014002, 1630052015050, 1630152017019, and 1630152016006), Central Public-interest Scientific Institution Basal Research Fund for Innovative Research Team Program of CATAS (NoO.17CXTD-28).

Formatted: Line spacing: 1.5 lines

Formatted: Left, Line spacing: 1.5 lines

Formatted: Font: Times New Roman, 9 pt

Formatted: Font: Times New Roman

Author's contribution

YX, HF, YY, MP, QL, AG designed the study and contribute to the project coordination; XY, PX, WX wrote the paper; LZ, JL, YW collected the samples and extracted the genomic DNA; YX, BL, BS, JX, AA, EI, NL conducted the genome analyses.

Acknowledgements

Annaliese S. Mason is gratefully acknowledged for assistance with language editing and manuscript revisions.

References

1. Batugal P, V Ramanatha Rao and J Oliver, editors. Coconut Genetic Resources. International Plant Genetic Resources Institute – Regional Office for Asia, the Pacific and Oceania (IPGRI-APO) Serdang, Selangor DE, Malaysia; 2005.
2. Tang B, Tang M, Chen C, Qiu P, Liu Q, Wang M, et al. Characteristics of soil fauna community in the Dongjiao coconut plantation ecosystem in Hainan, China. Acta Ecologica Sinica. 2006;26(1):26-32. doi:http://dx.doi.org/10.1016/S1872-2032(06)60003-6.
3. Singh R, Ong-Abdullah M, Low ET, Manaf MA, Rosli R, Nookiah R, et al. Oil palm genome sequence reveals divergence of interfertile species in Old and New worlds. Nature. 2013;500(7462):335-9. doi:10.1038/nature12309.
4. Al-Dous EK, George B, Al-Mahmoud ME, Al-Jaber MY, Wang H, Salameh YM, et al. De novo genome sequencing and comparative genomics of date palm (Phoenix dactylifera). Nature biotechnology. 2011;29(6):521-7. doi:10.1038/nbt.1860.
5. Al-Mssallem IS, Hu S, Zhang X, Lin Q, Liu W, Tan J, et al. Genome sequence of the date palm Phoenix dactylifera L. Nature communications. 2013;4:2274. doi:10.1038/ncomms3274.
6. Murray MG and Thompson WF. Rapid isolation of high molecular weight plant DNA. Nucleic acids research. 1980;8(19):4321-5.
7. Luo R, Liu B, Xie Y, Li Z, Huang W, Yuan J, et al. SOAPdenovo2: an empirically improved memory-efficient short-read de novo assembler. Gigascience. 2012;1(1):18. doi:10.1186/2047-217X-1-18.
8. Fan H, Xiao Y, Yang Y, Xia W, Mason AS, Xia Z, et al. RNA-Seq analysis of Cocos nucifera: transcriptome sequencing and de novo assembly for subsequent functional genomics approaches. PloS one. 2013;8(3):e59997. doi:10.1371/journal.pone.0059997.
9. Kent WJ. BLAT--the BLAST-like alignment tool. Genome Research. 2002;12(4):656-64. doi:10.1101/gr.229202. .
10. Simao FA, Waterhouse RM, Ioannidis P, Kriventseva EV and Zdobnov EM. BUSCO: assessing genome assembly and annotation completeness with single-copy orthologs. Bioinformatics (Oxford, England). 2015;31(19):3210-2. doi:10.1093/bioinformatics/btv351.
11. Jurka J, Kapitonov VV, Pavlicek A, Klonowski P, Kohany O and Walichiewicz J. Repbase Update, a database of eukaryotic repetitive elements. Cytogenetic and genome research. 2005;110(1-4):462-7. doi:10.1159/000084979.
12. Tarailo-Graovac M and Chen N. Using RepeatMasker to identify repetitive elements in genomic sequences. Current protocols in bioinformatics. 2009;Chapter 4:Unit 4.10.

Formatted: Font: Times New Roman, 9 pt

Formatted: Font: Times New Roman

doi:10.1002/0471250953.bi0410s25.

13. Xu Z and Wang H. LTR_FINDER: an efficient tool for the prediction of full-length LTR retrotransposons. Nucleic acids research. 2007;35(Web Server issue):W265-8. doi:10.1093/nar/gkm286.

14. Benson G. Tandem repeats finder: a program to analyze DNA sequences. Nucleic acids research. 1999;27(2):573-80.

15. The Arabidopsis Genome Initiative, Analysis of the genome sequence of the flowering plant Arabidopsis thaliana. Nature. 2000;408(6814):796-815. doi:http://www.nature.com/nature/journal/v408/n6814/suppinfo/408796a0_S1.html.

16. Goff SA, Ricke D, Lan TH, Presting G, Wang R, Dunn M, et al. A draft sequence of the rice genome (Oryza sativa L. ssp. japonica). Science (New York, NY). 2002;296(5565):92-100. doi:10.1126/science.1068275.

17. Paterson AH, Bowers JE, Bruggmann R, Dubchak I, Grimwood J, Gundlach H, et al. The Sorghum bicolor genome and the diversification of grasses. Nature. 2009;457(7229):551-6. doi:10.1038/nature07723.

18. Schnable PS, Ware D, Fulton RS, Stein JC, Wei F, Pasternak S, et al. The B73 maize genome: complexity, diversity, and dynamics. Science (New York, NY). 2009;326(5956):1112-5. doi:10.1126/science.1178534.

19. Altschul SF, Madden TL, Schaffer AA, Zhang J, Zhang Z, Miller W, et al. Gapped BLAST and PSI-BLAST: a new generation of protein database search programs. Nucleic acids research. 1997;25(17):3389-402.

20. Birney E, Clamp M and Durbin R. GeneWise and Genomewise. Genome Research. 2004;14(5):988-95. doi:10.1101/gr.1865504.

21. Stanke M, Keller O, Gunduz I, Hayes A, Waack S and Morgenstern B. AUGUSTUS: ab initio prediction of alternative transcripts. Nucleic acids research. 2006;34(Web Server issue):W435-9. doi:10.1093/nar/gkl200.

22. Burge C and Karlin S. Prediction of complete gene structures in human genomic DNA. Journal of molecular biology. 1997;268(1):78-94. doi:10.1006/jmbi.1997.0951.

23. Elsik CG, Mackey AJ, Reese JT, Milshina NV, Roos DS and Weinstock GM. Creating a honey bee consensus gene set. Genome biology. 2007;8(1):R13. doi:10.1186/gb-2007-8-1-r13.

24. Trapnell C, Pachter L and Salzberg SL. TopHat: discovering splice junctions with RNA-Seq. Bioinformatics (Oxford, England). 2009;25(9):1105-11. doi:10.1093/bioinformatics/btp120.

25. Trapnell C, Williams BA, Pertea G, Mortazavi A, Kwan G, van Baren MJ, et al. Transcript assembly and quantification by RNA-Seq reveals unannotated transcripts and isoform switching during cell differentiation. Nature biotechnology. 2010;28(5):511-5. doi:10.1038/nbt.1621.

26. Majoros WH, Pertea M and Salzberg SL. TigrScan and GlimmerHMM: two open source ab initio eukaryotic gene-finders. Bioinformatics (Oxford, England). 2004;20(16):2878-9. doi:10.1093/bioinformatics/bth315.

27. Ogata H, Goto S, Sato K, Fujibuchi W, Bono H and Kanehisa M. KEGG: Kyoto Encyclopedia of Genes and Genomes. Nucleic acids research. 1999;27(1):29-34.

28. Bairoch A and Apweiler R. The SWISS-PROT protein sequence database and its supplement TrEMBL in 2000. Nucleic acids research. 2000;28(1):45-8.

29. Jones P, Binns D, Chang HY, Fraser M, Li W, McAnulla C, et al. InterProScan 5: genome-scale

Formatted: Font: Times New Roman, 9 pt

Formatted: Font: Times New Roman

protein function classification. Bioinformatics (Oxford, England). 2014;30(9):1236-40. doi:10.1093/bioinformatics/btu031.

30. Bateman A, Birney E, Durbin R, Eddy SR, Howe KL and Sonnhammer EL. The Pfam protein families database. Nucleic acids research. 2000;28(1):263-6.

31. Attwood TK, Croning MDR, Flower DR, Lewis AP, Mabey JE, Scordis P, et al. PRINTS-S: the database formerly known as PRINTS. Nucleic acids research. 2000;28(1):225-7.

32. Corpet F, Gouzy J and Kahn D. Recent improvements of the ProDom database of protein domain families. Nucleic acids research. 1999;27(1):263-7.

33. Schultz J, Copley RR, Doerks T, Ponting CP and Bork P. SMART: a web-based tool for the study of genetically mobile domains. Nucleic acids research. 2000;28(1):231-4.

34. Mi H, Huang X, Muruganujan A, Tang H, Mills C, Kang D, et al. PANTHER version 11: expanded annotation data from Gene Ontology and Reactome pathways, and data analysis tool enhancements. Nucleic acids research. 2017;45(Database issue):D183-D9. doi:10.1093/nar/gkw1138.

35. Selengut JD, Haft DH, Davidsen T, Ganapathy A, Gwinn-Giglio M, Nelson WC, et al. TIGRFAMs and Genome Properties: tools for the assignment of molecular function and biological process in prokaryotic genomes. Nucleic acids research. 2007;35(Database issue):D260-D4. doi:10.1093/nar/gkl1043.

36. Wilson D, Pethica R, Zhou Y, Talbot C, Vogel C, Madera M, et al. SUPERFAMILY--sophisticated comparative genomics, data mining, visualization and phylogeny. Nucleic acids research. 2009;37(Database issue):D380-6. doi:10.1093/nar/gkn762.

37. Burge S, Kelly E, Lonsdale D, Mutowo-Muellenet P, McAnulla C, Mitchell A, et al. Manual GO annotation of predictive protein signatures: the InterPro approach to GO curation. Database : the journal of biological databases and curation. 2012;2012:bar068. doi:10.1093/database/bar068.

38. Li L, Stoeckert CJ, Jr. and Roos DS. OrthoMCL: identification of ortholog groups for eukaryotic genomes. Genome Research. 2003;13(9):2178-89. doi:10.1101/gr.1224503.

39. Edgar RC. MUSCLE: multiple sequence alignment with high accuracy and high throughput. Nucleic acids research. 2004;32(5):1792-7. doi:10.1093/nar/gkh340.

40. Guindon S, Dufayard JF, Lefort V, Anisimova M, Hordijk W and Gascuel O. New algorithms and methods to estimate maximum-likelihood phylogenies: assessing the performance of PhyML 3.0. Systematic biology. 2010;59(3):307-21. doi:10.1093/sysbio/syq010.

41. Yang Z. PAML 4: phylogenetic analysis by maximum likelihood. Molecular biology and evolution. 2007;24(8):1586-91. doi:10.1093/molbev/msm088.

42. Tuskan GA, Difazio S, Jansson S, Bohlmann J, Grigoriev I, Hellsten U, et al. The genome of black cottonwood, Populus trichocarpa (Torr. & Gray). Science (New York, NY). 2006;313(5793):1596-604. doi:10.1126/science.1128691.

43. Shi H, Lee BH, Wu SJ and Zhu JK. Overexpression of a plasma membrane Na+/H+ antiporter gene improves salt tolerance in Arabidopsis thaliana. Nature biotechnology. 2003;21(1):81-5. doi:10.1038/nbt766.

44. Brini F, Hanin M, Mezghani I, Berkowitz GA and Masmoudi K. Overexpression of wheat Na+/H+ antiporter TNHX1 and H+-pyrophosphatase TVP1 improve salt- and drought-stress tolerance in Arabidopsis thaliana plants. Journal of experimental botany. 2007;58(2):301-8. doi:10.1093/jxb/erl251.

Formatted: Font: Times New Roman, 9 pt

Formatted: Font: Times New Roman

45. Yong JW, Ge L, Ng YF and Tan SN. The chemical composition and biological properties of coconut (Cocos nucifera L.) water. Molecules (Basel, Switzerland). 2009;14(12):5144-64. doi:10.3390/molecules14125144.

Tables

Table 1 Data outputs produced by sequencing different insert size libraries

Library type	Lane	Reads Length(bp)	Insert Size(bp)	Raw data (Gb)	Clean data(Gb)
PE101	3	100	170	128.75(53.20)	111.32(46)
PE251	2	250	450	73.86(30.52)	56.42(23.31)
PE101	2	100	500	64(26.45)	65.11(26.90)
PE101	2	100	800	78.16(32.30)	64.90(26.82)
MP50	3	49	2000	128.6(53.14)	60.70(25.08)
MP50	2	49	5000	71.75(29.65)	18.62(7.69)
MP50	2	49	10000	74.65(30.85)	18.53(7.66)
MP50	2	49	20000	70.7(29.21)	19.35(7.99)
MP50	1	49	40000	24.2(10.08)	4.13(1.71)
Total	19			714.67(295.32)	419.08(173.17)

Note: The sequencing depth was shown in parentheses, calculated based on a genome size of 2.42G. Clean data were obtained by filtering raw data with low-quality and duplicate reads. PE: paired-end, MP: mate pair.

Table 2 Comparison analysis of genome sizes, assembly and annotation of four palmae species, including coconut, *Phoenix dacylifera* (PDK30 and DPV01, two different versions), *Elaeis guineensis* (EG), and *Elaeis oleifera* (EO)

Species	Sequencing technology	Sequence coverage	Estimated size(Gb)	Assembly size(Gb)	Contig N50(Kb)	Scaffold N50(Kb)	Gene Number	TEs percent (%)
Phoenix dactylifera (PDK30)	Illumina GAIIx	53.4x	0.66	0.38	6.44	30.48	28,889	23.6
Phoenix dactylifera (DPV01)	454,SOLiD, ABI3730	139x	0.67	0.56	10.81	334.08	41,660	38.87
Elaeis guineensis (African oil palm)	454	16X	1.8	1.54	9.37	1045.41	34,802	43.24
Elaeis oleifera (American oil palm)	454	16x	1.8	1.40	8.45	333.11	--	--
Cocos nucifera (Hai nan Tall)	Illumina HiSeq	173X	2.42	2.20	72.64	418.07	28,039	72.75

Note: Coconut: *Cocos nucifera* (Hai–nan Tall); PDK30: *Phoenix dactylifera* (PDK30); DPV01: *Phoenix dactylifera* (DPV01); EG: *Elaeis guineensis* (~~American~~ Africanoil palm E5 build); EO *Elaeis oleifera* (American oil palm, O8-build); ~~The~~ TE~~s~~ results w~~as~~ereas obtained using the same pipeline as for the~~with the~~ ~~C~~coconut

Formatted: Left

Formatted: Font: Times New Roman, 9 pt

Formatted: Font: Times New Roman

genome

Table 3 The gene coverage of *Cocos nucifera* ~~by~~ based on transcriptome data

Dataset	Number	Total length (bp)	Base coverage by assembly	Sequencecoverage by assembly (%)
All	57,304	43,090,665	96.78	99.57
>200bp	57,304	43,090,665	96.78	99.57
>500bp	25,713	33,470,388	96.36	99.85
>1000bp	13,796	25,004,919	95.99	99.94

Table 4 The comparative analysis of assembly results of five palm species with BUSCO software, including coconut, *Phoenix dacylifera* (PDK30 and DPV01, two varieties), *Elaeis guineensis* (EG), and *Elaeis oleifera* (EO)

	Coconut		PDK30		DPV01		EG		EO	
BUSCOs	N	P (%)	N	P (%)	N	P (%)	N	P(%)	N	P(%)
Total	1440		1440		1440		1440		1440	
Complete single-copy	1192	82.8	1042	72.4	1160	80.6	1100	76.4	1004	69.7
Complete duplicated	115	8.0	81	5.6	134	9.3	116	8.1	63	4.4
Fragment	49	3.4	98	6.8	42	2.9	60	4.2	84	5.8
Missing	84	5.8	219	15.2	104	7.2	164	11.3	289	20.1

Note: Coconut: *Cocos nucifera* (the Hainan Tall); PDK30: *Phoenix dactylifera* (PDK30); DPV01:*Phoenix dactylifera* (DPV01); EG: *Elaeis guineensis*(African~~merican~~ oil palm E5 build); EO *Elaeis oleifera* (American oil palm, O8-build);

Table 5 Classification of predicted transposable elements in the coconut genome

	Repabse TEs length	Protein TEs length	*De novo* TEs length	Combined TEs length	percentage
DNA	20,936,158	24,655,089	35,131,002	58,119,982	2.64
LINE	4,251,185	9,631,472	7,610,172	19,197,064	0.87
SINE	85,717	0.00	186,364	270,055	0.012
LTR	361,968,154	512,700,933	1,419,281,798	1,478,182,089	67.10
Other	8,145	0.00	0.00	8,145	0.0004
Unknown	0.00	12,360	139,084,335	139,096,695	6.31
Total	385,037,442	546,965,774	1,552,582,881	1,602,630,396	72.75

Formatted: Left

Formatted: Font: Times New Roman, 9 pt

Formatted: Font: Times New Roman

Note: Repabse TEs means RepeatMask against Repbase; Protein TEs means RepeatProteinMask result against Repbase protein; *De novo* TEs means RepeatMask against the *de novo* library; Combined TEs: means the combined results of these three steps.

Table 6 The comparative analysis of gene prediction results of four palm species with BUSCO software

	Coconut		PDK30		DPV01		EG	
BUSCOs	N	P (%)	N	P (%)	N	P (%)	N	P (%)
Total	1440		1440		1440		1440	
Complete single-copy	965	74.1	748	51.9	1195	83.0	555	38.5
Complete duplicated	102	7.1	81	5.6	159	11.0	53	3.7
Fragment	162	11.2	255	17.7	44	3.1	270	18.8
Missing	211	14.7	356	24.8	42	2.9	562	39.0

Note: Coconut: *Cocos nucifera* (the Hai-nan Tall); PDK30: *Phoenix dactylifera* (PDK30); DPV01: *Phoenix dactylifera* (DPV01); EG: *Elaeis guineensis* (AfricanAmerican oil palm E5 build); The gene of *Elaeis oleifera* (American oil palm, O8-build) was missing, not attained from the public database;

Table 7 Statistical analysis of gene families of different species

Species	Genes number	Genes in families	Unclustered genes	Family number	Unique families	Average genes per family
C. nucifera	28,039	22,376	5,663	14,411	282	1.55
E. guineensis	30,430	22,021	8,409	13,415	262	1.64
P. dactylifera	24,908	22,193	2,715	14,074	112	1.58
S. bicolor	27,159	22,016	5,143	12,992	916	1.69
P. persica	27,792	24,276	3,516	14,443	497	1.68
S. tuberosum	34,879	28,288	6,591	13,206	1,119	2.14
G. max	42,859	38,104	4,755	14,589	1,145	2.61
A. thaliana	26,637	22,990	3,647	13,292	674	1.73
T. cacao	28,624	23,776	4,848	14,928	625	1.59
V. vinifera	25,329	19,122	6,207	13,309	599	1.44
M. acuminata	36,538	24,354	12,184	13,089	620	1.86

Formatted: Left

Formatted: Font: Times New Roman, 9 pt

Formatted: Font: Times New Roman

Figure legends

Figure 1 Morphological characteristic of coconut tree (A), spica (B), female flower (C), Male flower (D), –coconut nut (E), coconut nut without skin (F), and –vertical section of coconut nut (G).

Figure 2 Kmer analysis of the coconut genome.

Figure 3 The protocol for integrating GLEAN and Transcriptome transcriptome data.

Figure 4 Groups of orthologues shared among the angiosperms *Cocos nucifera* (Coconut), *Elaeis guineensis* (Oil palm), *Phoenix dactylifera* (Date palm), *Sorghum bicolor* (Sorghum), *Musa acuminate* (Banana) and *Arabidopsis thaliana* (Arabidopsis). Veenn diagram generated by http://www.interactivenn.net/.

Figure 5. Estimation of divergence time. The blue numbers on the nodes are the divergence time from present (million years ago, Mya), the red nodes indicated the previously published calibration times.

Figure 6. Phylogenetic tree of antiporter genes from *C. nucifera* and *Arabidopsis thaliana*. Every cluster wais indicated with a different colored arc line arc. The potential function of every cluster wais indicated with the function groups found in *Arabidopsis thaliana*. Colored stars indicate antiporter genes of *C. nucifera.*

Figure 7. Phylogenetic tree of ion channel genes from *C. nucifera* and *Arabidopsis thaliana*. Every cluster was indicated with different colored arc line arc. The potential function of every cluster was indicated with the function groups found in *Arabidopsis thaliana*. Colored stars indicate ion channel genes of *C. nucifera.*

Formatted: Font: Times New Roman, 9 pt

Formatted: Font: Times New Roman

~~Figure 4Groups of orthologues shared among the angiosperms *Cocos nucifera*(Coconut), *Elaeis guineensis*(Oil palm), *Phoenix dactylifera*(Date palm), *Sorghum bicolor*(Sorghum), *Musa acuminate*(Banana) and *Arabidopsis thaliana*(Arabidopsis). Veen diagram generated by http://www.interactivenn.net/.~~

~~Figure 5. Estimation of divergence time. The blue numbers on the nodes are the divergence time from present (million years ago, Mya), the red node indicated the previously published calibration times.~~

~~Figure 6. Phylogenetic tree of atiporter genes from *C. nucifera* and *Arabidopsis thaliana*. Every cluster was indicated with different colored arc line. The potential function of every cluster was indicated with the function groups found in *Arabidopsis thaliana*. Colored stars indicate antiporter genes of *C. nucifera*.~~

~~Figure 7. Phylogenetic tree of ion channel genes from *C. nucifera* and *Arabidopsis thaliana*. Every cluster was indicated with different colored arc line. The potential function of every cluster was indicated with the function groups found in *Arabidopsis thaliana*. Colored stars indicate ion channel genes of *C. nucifera*.~~

Additional files

Additional file 1 Identification and characterization of antiporter genes in the genome of Cocos nucifera

Additional file 2 Identification and ~~charaterization~~characterization of ion channel genes in the genome of Cocos nucifera

Formatted: Font: Times New Roman, 9 pt

Formatted: Font: Times New Roman

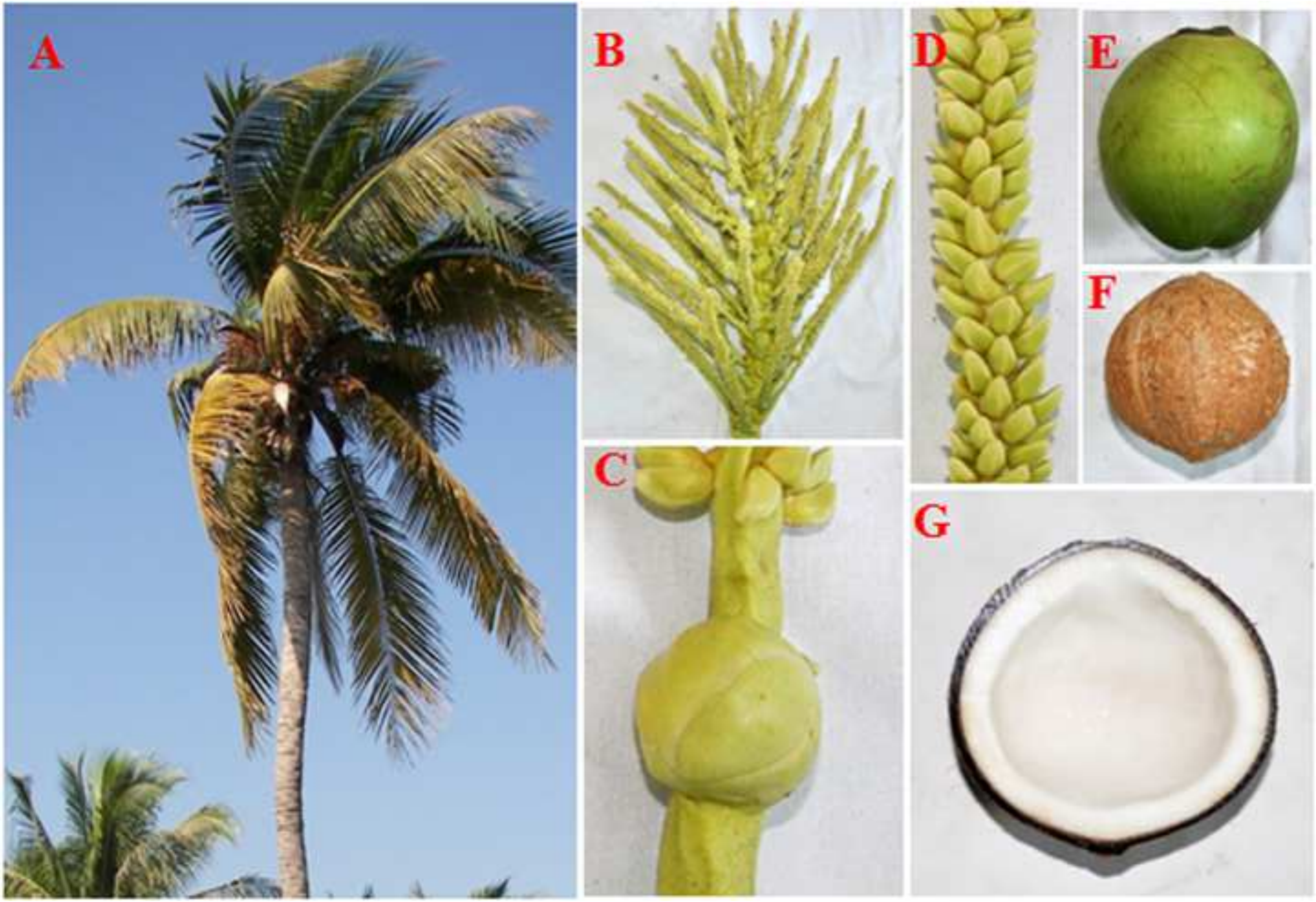
A
B
C
D
E
F
G

Click here to download Figure Figure 2.tiff

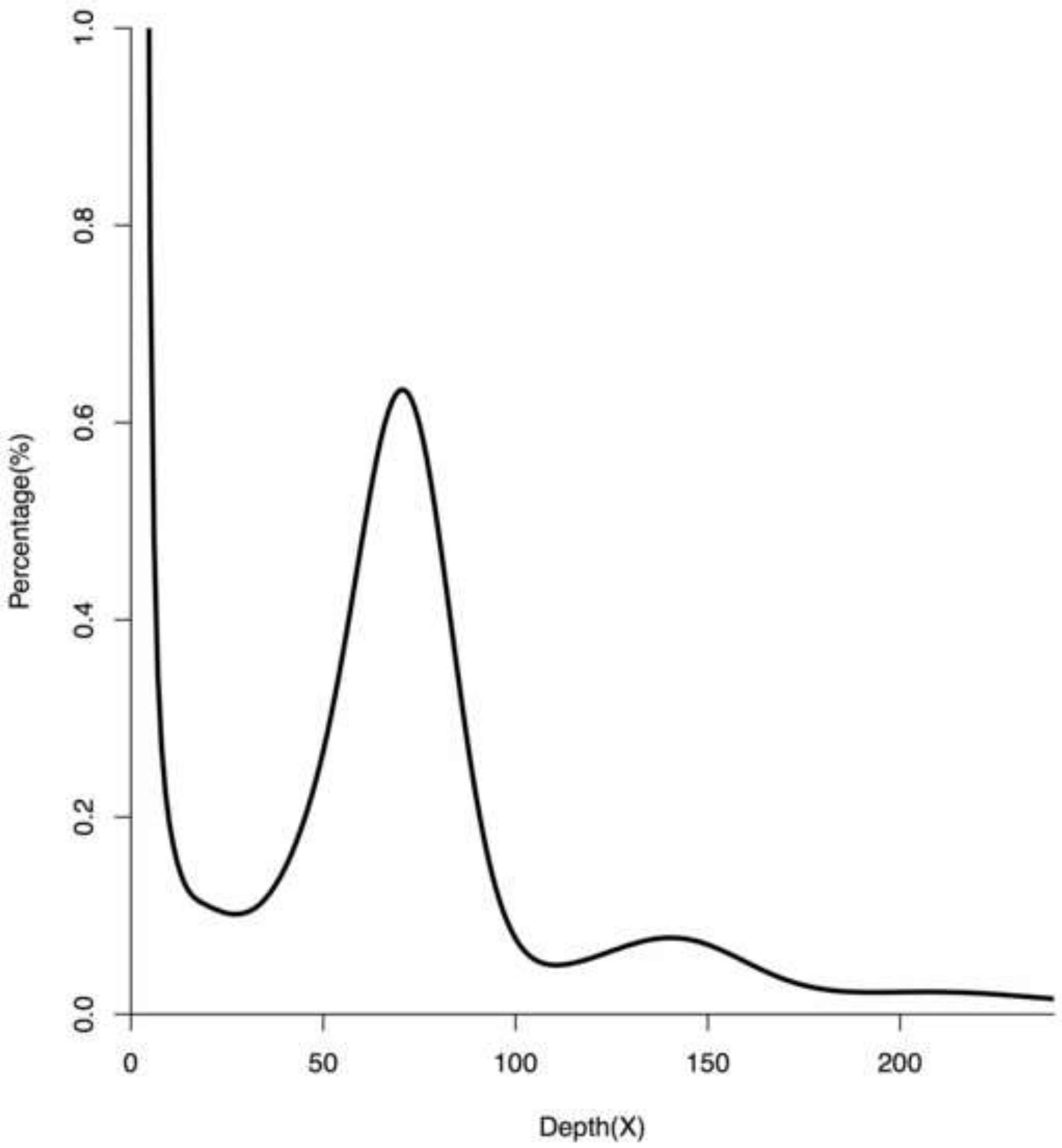

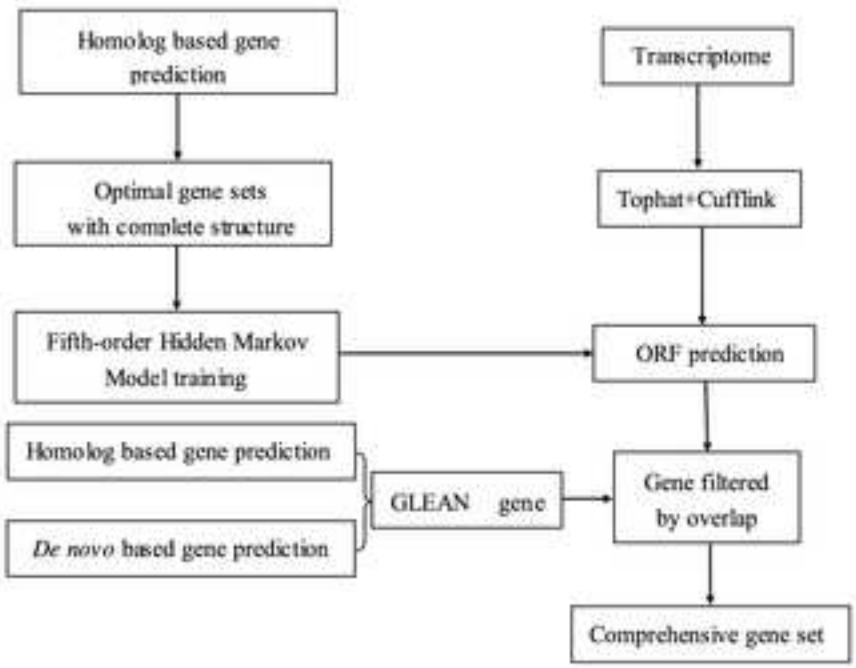
Homolog based gene prediction
Transcriptome
Optimal gene sets with complete structure
Tophat+Cufflink
Fifth-order Hidden Markov Model training
ORF prediction
Homolog based gene prediction
GLEAN gene
Gene filtered by overlap
De novo based gene prediction
Comprehensive gene set

Figure4 Click here to download Figure Figure 4-2.jpg

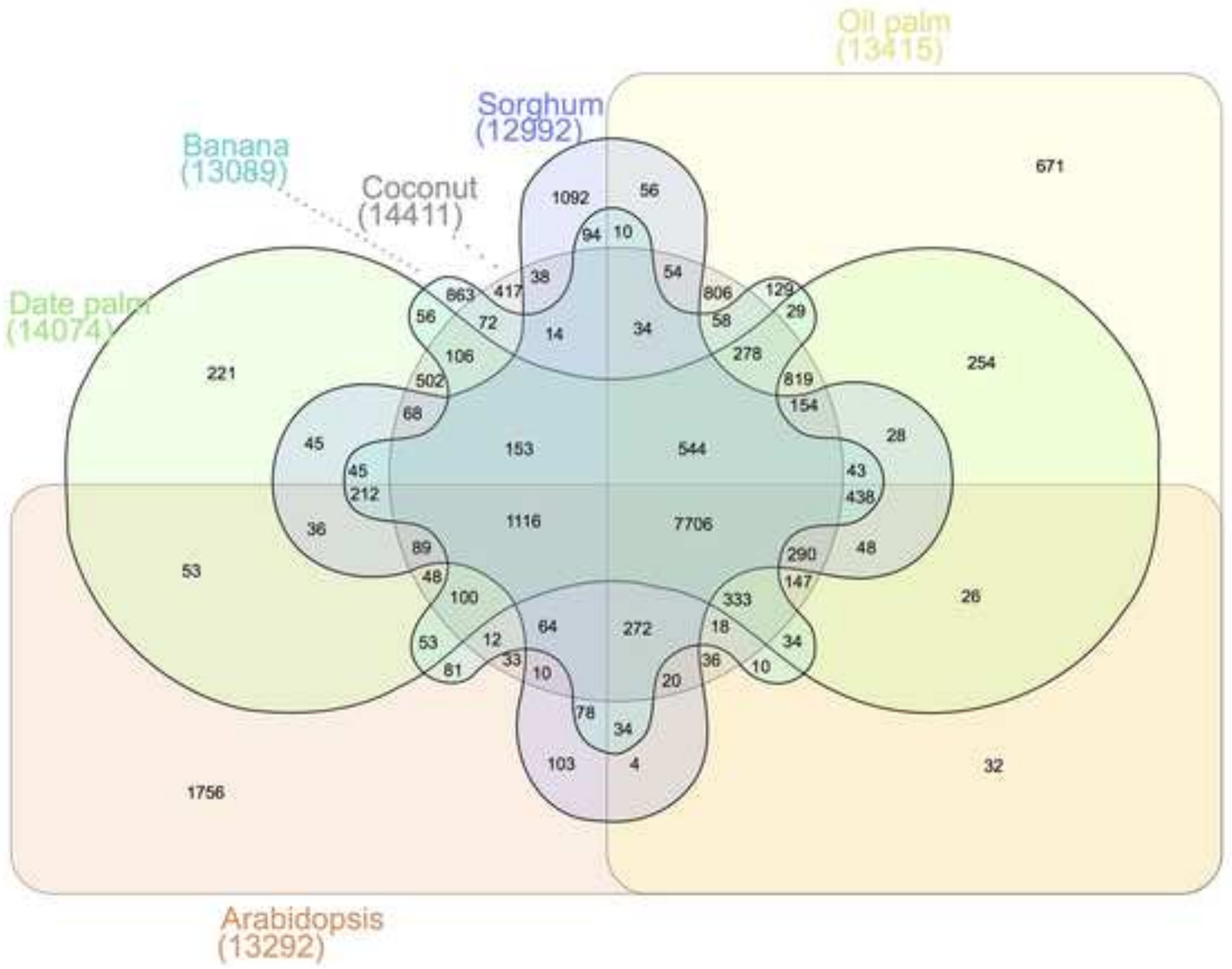

Click here to download Figure Figure 5-2.jpg

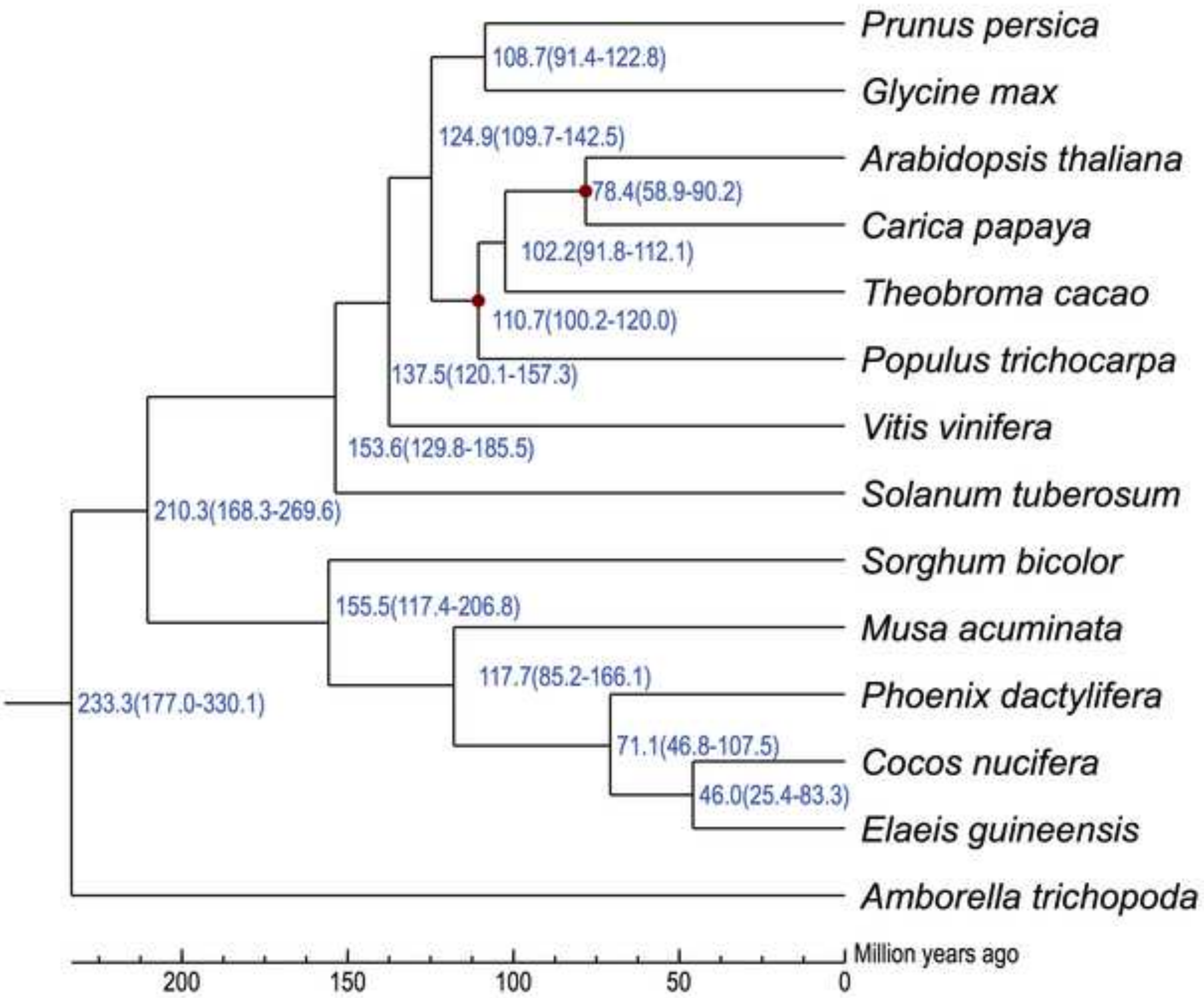

Figure6

Click here to download Figure Figure 6.tif

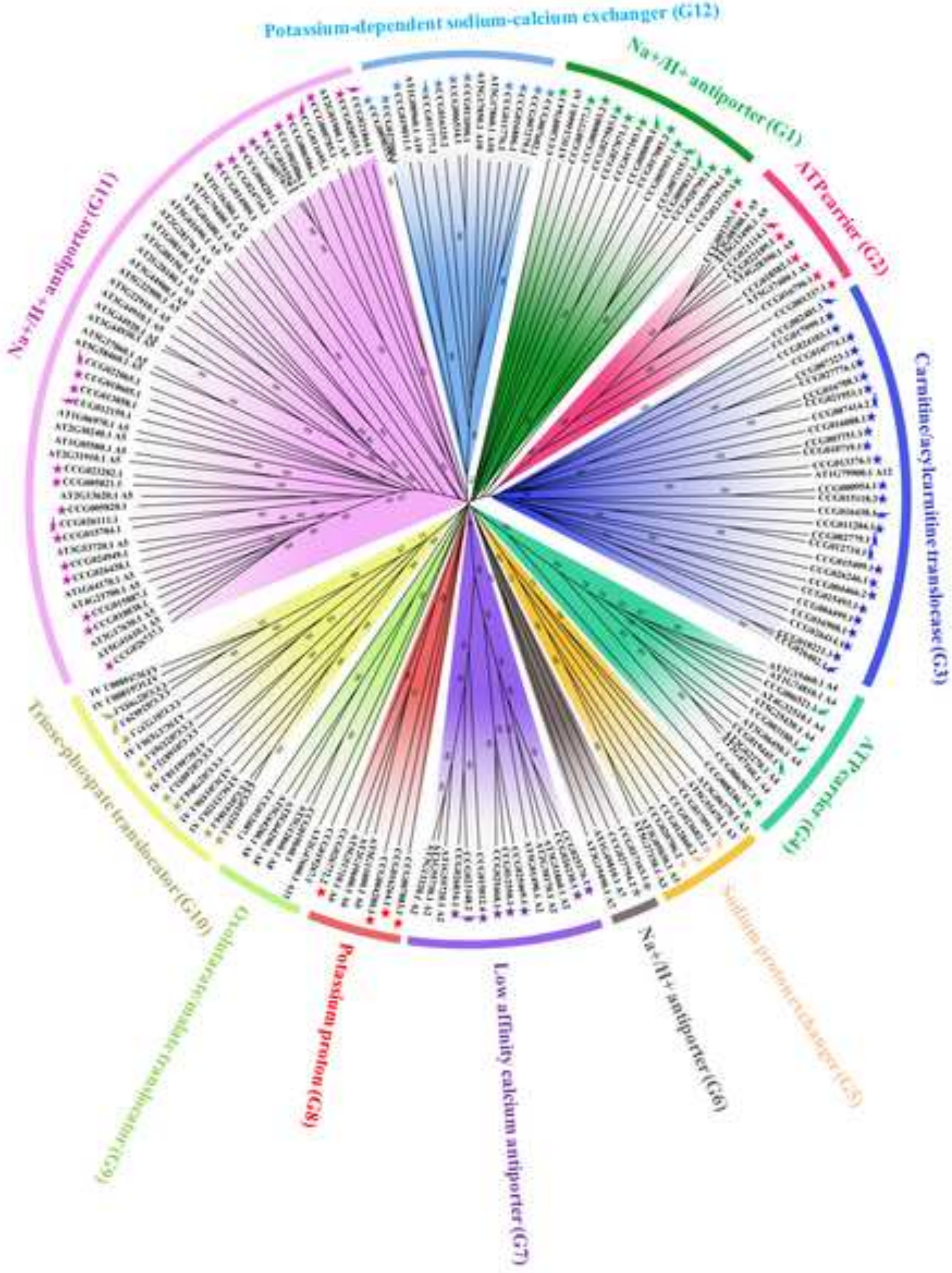

Figure7

Click here to download Figure Figure 7.tif

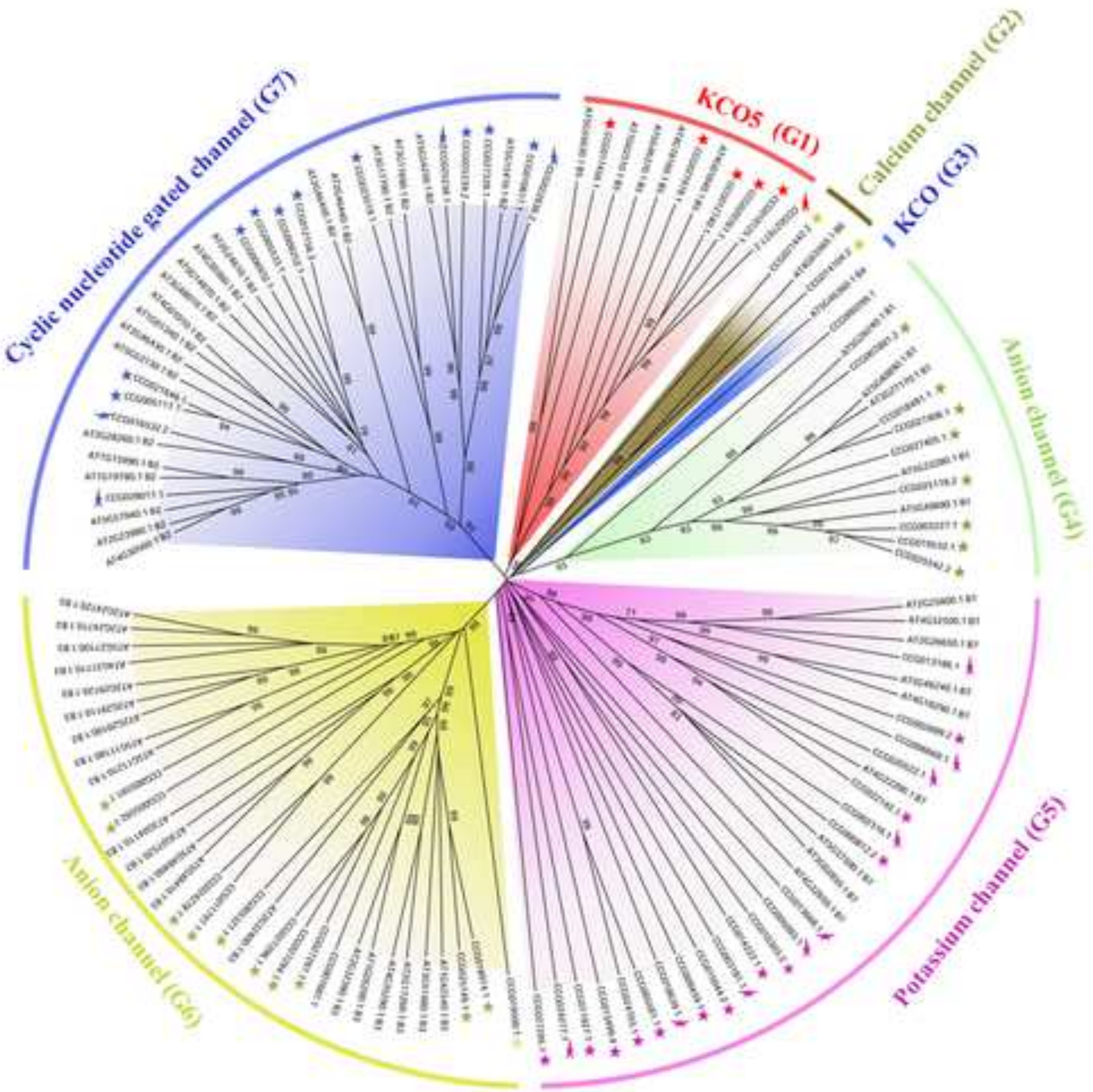

Additional file 1

Click here to access/download
Supplementary Material
Additional file 1.xlsx

Additional file 2

Click here to access/download
Supplementary Material
Additional file 2.xlsx

 Click here to download Personal Cover cover letter and response-6.11-20170719-xiao.docx

Response to editor and reviewers

Dear editor and reviewers

Thank you very much for your crucial comments for our manuscript entitled "The genome draft of the Coconut (*Cocos nucifera*)" (GIGA-D-17-00038). We have made a thorough revision to the ms based on all of comments from editor and reviewers. Each comments raised by the reviewers had been carefully answered in the response sheet. We hope the revised version can meet the requirement of "GigaScience"

Sincerely yours,

Yaodong Yang

PHONE NUMBER: 0086-898-63330602
FAX NUMBER: 0086-898-63330673
EMAIL: yyang@catas.cn
POSTAL ADDRESS:
Coconuts Research Institute, Chinese Academy of Tropical Agricultural Sciences,
496 Wenqing Av., Wenchang, Hainan 571339, P.R.China

Response to editor and reviewers,

Reviewer 1

1. Line 40: in 93 countries -> the introduction (line 70) say 89 countries

>>>Response: Thank you for your suggestion. We have re-checked the document reported by Batugal et al., 2005. The corresponding revision has been done in the Introduction part of the revised manuscript.

2. 11 million ha ->the introduction (line 72) says 12 million ha

>>>Response: Thank you for your suggestion; we have re-checked the plant area of coconut in the website of Food and Agriculture Organization of the United Nations (http://www.fao.org/faostat/en/). The corresponding revision has been done in the Abstract part of the revised manuscript.

3. Hinders progress in genetic breeding. Do you mean 'marker assisted breeding' or 'genomic assisted breeding'?

>>>Response: Thank you for your suggestion; we meant to say ‘conventional breeding’. Revisions have been made in the Abstract part of the revised manuscript to make our opinions clearer.

4. Genetic improvement is slow. Do you mean trait improvement with marker or genetic assisted

>>>Response: We meant to say the improvement made by ‘conventional breeding’ is slow. The corresponding revision has been done in the revised manuscript.

5. Line 48: The coverage does not add up. 714.67 Gb on a 2.42 Gb genome is 295× coverage. In any case, only the coverage of the cleaned reads should be shown (177×)`

>>>Response: Thank you for your suggestion; in revised manuscript, only the cleaned reads were used for the coverage depth analysis and the coverage is173.32× read depth.

6. Line54: Do you mean 41,166 genes

>>>Response: Thank you for your suggestion; we have re-checked the annotated gene number for datepalm based on the document reported by AI-Mssallem et al., 2013 and 41 660 genes were annotated. The corresponding revisions have been made in the Abstract part of revised manuscript.

7. Line60: space missing between facilitating and future

>>>Response: Thank you for your suggestion, a space has been added between facilitating and future.

8. Line 61: should be ‘molecular assisted breeding’

>>>Response: Thank you for your suggestion, corresponding revisions have been done in the Abstract part of revised version.

9. Line 78: ‘…wide range to environment…’ -> unclear, should be explained. Also ‘environment’

>>>Response: Some sentences have been added to the revised manuscript for explaining ‘…wide range to environment…’ in Line 240– Line 242|Page 3.

10. Line78: ‘…especially for high tolerance to high salt density.’ , please clarify

>>>Response: Coconut palm can disseminate through ocean currents: floating nuts sprout and

grow naturally upon washing up on beaches. The ability to adapt to a high salt environment is closely related to this dissemination feature and to these natural growth conditions. Corresponding revision has been done in Line 243– Line 244|Page 3 of revised manuscript.

11. Line 80: '…making it possible to understand its adaptation to high salinity.' You do not investigate this, you should change the statement to something milder such as: 'This study forms the basis for future research investigating the coconuts tolerance to salt stress'

>>>Response: Thank you for your suggestion, We also present the genome sequence of HAT coconut and added an analysis of the antiporter and ion channel gene families, relevant to salinity tolerance, into the revised version. Corresponding revision had been added into in Line 237– Line 238|Page 3.

12. Line 82: provide references. The way this sentence reads at the moment, make it seem like you are also reporting those genome sequence.

>>>Response: The corresponding references have been added into Line 423|Page 4 of revised manuscript.

13. Line 92: space between 'Illumina', 'Hiseq2000' and 'sequencer'

>>>Response: Two spaces had been added into between Illumina, Hiseq2000 and sequencer in Line 436|Page 4 of revised manuscript.

14. Line129: The data shows that you have higher coverage and a longer N50, it does not show that the assembly is of better quality.

>>>Response: Thank you for your suggestion, the sentence has been replace by other sentence: "The comparative results of the BUSCO estimation in coconut and in the four other palm genome sequences indicates that the smallest fraction of missing genes as predicted by BUSCO was found in the coconut genome assmebly", in Line 724 – Line 726|Page 6 of revised version.

15. Line 131: 'tissues', not 'issues'

>>>Response: Thank you for your suggestion, corresponding revisions has been done in the revised version.

16. Line134: table 4 and 5 are mixed up

>>>Response: We repeatedly checked Table 4 and 5. Corresponding revisions has been done in revised manuscript.

17. Line 165: BLAST not BLSAT

>>>Response: Thank you for your suggestion, 'BLSAT' had been modified in revised manuscript.

18. Line 175 (and others): keep a space between numbers and units, consistently.

>>>Response: we re-checked all numbers and units throughout the manuscript. All needed spaces have been added between numbers and units.

19. Line195: Change start of sentence (e.g. 'After the above described steps…')

>>> Response: Thank you for your suggestion, corresponding revision has been done in Line 970|Page 8 of the revised manuscript.

20. Line 196: should read: 'than the predicted gene markers..'

>>>Response: Thank you for your suggestion, corresponding revision has been done in Line 971 | Page 8 of revised version.

21. Line203: space between 'by' and 'sequence'

>>>Response: Thank you for your suggestion, a space had been added between by and sequence

22. Line211: after ref 38, just one dot

>>>Response: Thank you for your suggestion, the ref 38 and dot has been deleted in revised version.

23. Line 219: remove space between 'mapping' and ','

>>>Response: Thank you for your suggestion, the space has been deleted between 'mapping' and ','.

24. References: need a lot of editing to uniform

>>>Response: All references of the manuscript have been reviewed and edited based on the author guideline of "Gigascience" in the revised manuscript.

25. Tables: Headers are unclear and many abbreviations within tables are not explained

>>>Response: Thank you for your suggestion, revisions have been done for the table headers. Meanwhile, the abbreviations have been explained and replaced with corresponding full name.

26. What is the difference between Table 4 and Table 7? Both show BUSCO assessments of palm species. Clarify both in tables and in the text.

>>>Response: Thank you for your suggestion, Table 7 has been changed into Table 6 in the revised version. Table 4 referred to the comparative analysis of the assembled genome sequences for four palm species using BUSCO software, while Table 6 referred to the comparative analysis of the predicted gene from the four palm species using BUSCO software. Revisions have been done to make Table 4 and Table 6 legends more clearly in “Table” part of revised version.

27. Figure legend: Figure 1 does not contain any morphological characteristics; they are photographs of coconut plants.

>>>Response: Figure 1 had been substantially revised in the revised version.

Reviewer 2

1. My only major concern about the manuscript is that the written style is not ready for publication. There are many type and grammatical mistakes all over the main text, figure captions and table legends. The manuscript needs some extensive copy editing to be published.

>>>Response: Thank you for your suggestion, the manuscript has been reviewed and edited throughout the manuscript by the native experts (Annaliese Mason, Baudouin Luc and Amjad Iqbal).

Reviewer 3

1. Homologous gene families using a larger set of genomes would allow a gain-/loss analysis (check the Zostera (seagrass) genome paper Figure 1a for a recent example), some venn diagrams based on this showing how many gene are shared with close relative (e.g. *Elaeis*), other monocots (e.g. rice) and dicots (e.g. Arabidopsis) could also be generated based on this (e.g. orchid genome paper figure 1a). Asynteny/collinearity analyisis is usually included, often combined with a Ks analysis (see the orchid genome paper Figure 2, Zostera genome paper Figure 2).

>>>Response: Thank you for your suggestion, we added venn diagrams between different species and analyzed the divergence time between different species into Line 990| Page 8 - Line 1270 | Page 10 of the revised version. Meanwhile, we identified and characterized antiporter and ion channel gene family in Line 1271 | Page 10 – Line 1578 | Page 11 of revised manuscript.

2. No case study is included, I feel there should be at least one (though as the paper is submitted as a data note the journal might not require one). The authors are the first ones to have a glimpse at the genome of this species. I would make sense to check a few relevant gene families (coconut are clearly very different from seeds of other monocots, so seed related gene families would be likely candidates for a more in depth study)

>>>Response: Thank you for your suggestion. It is known that coconut palm can disseminate

through ocean currents: floating nuts sprout and grow naturally upon washing up on beaches. The ability to adapt to a high salt environment is closely related to this dissemination feature and to these natural growth conditions. In the revised manuscript, we identified antiporter and ion channel genes in the genome of *Cocos nucifera*, some of which had been validated to be associated with salt stress in Arabidopsis. In the gene expansions analysis, some gene families showed significant expansion in compared to Arabidospsis, including Na+/H+ antiporter family, Carnitine/acylcarnitine translocase family, Potassium-dependent sodium antiporter, and potassium channel. The expansion of Na+/H+ antiporter family and Potassium-dependent sodium antiporter may be associated with coconut salt tolerance. The expansion of carnitine/acylcarnitine translocase family may be associated with the accumulation of fatty acid in coconut pulp. At last, the expansion of potassium channel may be associated with the accumulation of potassium ion in coconut water. Corresponding revision had been added into Line Line 1271 | Page 10 – Line 1578 | Page 11 of revised manuscript.

3. For non-bioinformaticians a supplemental website which offers a BLAST interface would certainly be welcome.

>>>Response: we have uploaded coconut genome raw data into Sequence Read Archive (SRA) of the National Center for Biotechnology Information. The assembled and annotated data were uploaded into GigaDB database. Meanwhile, the assembled and annotated data have been uploaded into pirate website for blast analysis and genome browse. However, currently, this website is not available for all people. The website will be available after further website improvement and paper publication

4. Line 128 -129: The N50 by itself is not a direct measure for the quality of the assembly. Avoid over-interpretation.

>>>Response: Thank you for your suggestion, the sentence has been replace by other sentence: “The comparative results of the BUSCO estimation in coconut and in the four other palm genome sequences indicates that the smallest fraction of missing genes as predicted by BUSCO was found in the coconut genome assmebly”, in Line 724 – Line 726|Page 6 of revised version.

5. Line 54 and abstract: (DVP01, 4166) -> the number of genes for the date palm genome is incorrect. In table 2 the authors report 41, 660 !

>>>Response: Thank you for your suggestion; we have re-checked the annotated gene number for datepalm based on the document reported by AI-Mssallem et al., 2013 and 41 660 genes were annotated. The corresponding revisions have been made in the Abstract part of revised manuscript.

6. Line 60: facilitating future: missing space Line 78-79

>>>Response: Thank you for your suggestion, a space has been added into between facilitating and future.

7. Line 78-79: For high tolerant to high salt density: revise grammar

>>>Response: Thank you for your suggestion, revisions have been done in Line 240– Line 242 | Page 3 of revised manuscript

8. Line 80: …present Hainan Tall… -> …present the Hainan Tall

>>>Response: Thank you for your suggestion, the sentence has been rewrite and the usage of the phrase “Hainan Tall” has been carefully checked throughout the revised manuscript.

9. Line 82: …about genome

>>>Response: Revisions have been done in revised manuscript.

10. Line 88 (and other places): …pair end… -> …paired end…

>>>Response: Thank you for your suggestion, all ‘pair end’ has been modified into ‘paired end’ throughout the revised manuscript.

11. Line 96: …removed by using … -> …removed using…

>>>Response: Thank you for your suggestion, corresponding revision had been done in revised manuscript

12. Line 116: …SOAPdenovo2 map… -> …SOAPdenovo2 maps…

>>>Response: Thank you for your suggestion, corresponding revision had been done in Line 572 | Page 5 of revised manuscript.

13. Line 132: ...reported in previous Fan’s research …: incorrect grammar should be revised (as previously reported by Fan et al…).

>>>Response: Thank you for your suggestion, corresponding revision had been done in Line 598 | Page 5 in revised manuscript

14. Line 181: Previous Fan’s research: revise grammar

>>>Response: ‘Previous Fan’s research’ had been modified into ‘as previously reported by Fan et al.’ in Line 874 | Page 7 of revised manuscript

15. Line 192: … a diagrammic pipeline is showed…: revise grammar

>>>Response: Thank you for your suggestion, corresponding revisions had been done in Line

968|Page 8 revised version.

16. Line 199: …completely… -> complete

>>>Response: Thank you for your suggestion, corresponding revision has been done in Line 973 | Page 8 of revised manuscript.

17. Line 203 -204: In sequence similarity step: revise

>>>Response: ‘In sequence similarity step’ has been modified into ‘Firstly’ in Line 979|Page 8 of the revised manuscript.

18. Line 232-233: Font is suddenly somewhat bigger

>>>Response: Thank you for your suggestion, corresponding revision have been done in “Funding” part of revised manuscript.
