# Supplementary material for: The genome draft of coconut (Cocos nucifera)
Source: Gigascience. 2017 Oct 5;6(11):1–11. doi: 10.1093/gigascience/gix095 (PMC5714197; doi:10.1093/gigascience/gix095)
Supplement: GIGA-D-17-00038_Revision-2.pdf [file gix095_giga-d-17-00038_revision-2.pdf]

GigaScience

The genome draft of the coconut (Cocos nucifera)

--Manuscript Draft--

**Manuscript Number:**	GIGA-D-17-00038R2
**Full Title:**	The genome draft of the coconut (Cocos nucifera)
**Article Type:**	Data Note

Funding Information:

International Science and Technology Cooperation projects of Hainan Province (No. KJHZ2014-24)	Dr Yaodong Yang
Hainan Natural Science Foundation (313058)	Dr Wei Xia
the fundamental Scientific Research Funds for Chinese Academy of Tropical Agriculture Sciences (1630032012044)	Dr Yaodong Yang
the fundamental Scientific Research Funds for Chinese Academy of Tropical Agriculture Sciences (1630052014002)	Dr Yaodong Yang
the fundamental Scientific Research Funds for Chinese Academy of Tropical Agriculture Sciences (1630052015050)	Dr Yong Xiao
The major Technology Project of Hainan (ZDZX2013023-1)	Dr Ming Peng
the fundamental Scientific Research Funds for Chinese Academy of Tropical Agriculture Sciences (1630152017019)	Dr Yaodong Yang
the fundamental Scientific Research Funds for Chinese Academy of Tropical Agriculture Sciences (1630152016006)	Dr Yong Xiao
Central Public-interest Scientific Institution Basal Research Fund for Innovative Research Team Program of CATAS (17CXTD-28)	Dr Yaodong Yang

Abstract:

Background

Coconut palm (Cocos nucifera, 2n = 32), a member of genus Cocos and family Arecaceae (Palmaceae), is an important tropical fruit and oil crop. Currently, coconut palm is cultivated in 93 countries, including Central and South America, East and West Africa, Southeast Asia and the Pacific island, with a total growth area of more than 12 million hectares (www.fao.org/faostat/en/). Coconut palm is generally classified into two main categories: "Tall" (flowering 8-10 years after planting) and "Dwarf" (flowering 4-6 years after planting), based on morphological characteristics and breeding habits. This Palmae species has a long growth period before reproductive years which hinders conventional breeding progress. In spite of initial successes, improvements made by conventional breeding have been very slow. In the present study, we obtained de novo sequences of Cocos nucifera genome: a major genomic resource which could be used to facilitate molecular breeding in Cocos nucifera and accelerating the breeding process in this important crop.

Findings

A total of 419.67 gigabases (Gb) of raw reads were generated by the IlluminaHiSeq 2000 platform using a series of paired-end and mate-pair libraries, covering the predicted Cocos nucifera genome length (2.42Gb, variety "Hainan Tall") to an estimated 173.32× read depth. A total scaffold length of 2.20 Gb was generated (N50 = 418 Kb), representing 90.91% of the genome. The coconut genome was predicted to harbor 28,039 protein-coding genes, which is less than in Phoenix dactylifera (PDK30 variety: 28,889), Phoenix dactylifera (DPV01 variety: 41,660) and Elaeis guineensis (34,802). BUSCO evaluation demonstrated the obtained scaffold sequences covered 90.8% of the coconut genome, and that the genome annotation was 74.1% complete. Genome annotation results revealed that 72.75% of the coconut

	genome was consisted of transposable elements. of which long-terminal repeat retrotransposons elements (LTRs) accounted for the largest proportion (92.23%).<br>Comparative analysis of the antiporter gene family and ion channel gene families between C. nucifera and Arabidopsis thaliana indicated that significant gene expansion may occurred in coconut involving Na+/H+ antiporter, Carnitine/acylcarnitine translocase, Potassium-dependent sodium-calcium exchanger, and potassium channel genes.<br>Conclusions<br>Despite its agronomic importance, C. nucifera is still under-studied. In this report, we made an attempt to construct a draft genome of C. nucifera and provide an enormous amount of genomic information that will facilitate future functional genomics and molecular assisted breeding in this crop species.
**Corresponding Author:**	Yaodong Yang, Ph.D<br>Coconut Research Institute<br>Wenchang City, Hainan CHINA
**Corresponding Author Secondary Information:**	
**Corresponding Author's Institution:**	Coconut Research Institute
**Corresponding Author's Secondary Institution:**	
**First Author:**	Yong Xiao
**First Author Secondary Information:**	
**Order of Authors:**	Yong Xiao
	Pengwei Xu
	Haikuo Fan
	Luc Baudouin
	Wei Xia
	Stéphanie Bocs
	Junyang Xu
	Qiong Li
	Anping Guo
	Lixia Zhou
	Jing Li
	Yi Wu
	Zilong Ma
	Alix Armero
	Auguste Emmanuel Issali
	Na Liu
	Ming Peng
	Yaodong Yang
**Order of Authors Secondary Information:**	
**Response to Reviewers:**	Response to editor and reviewers<br><br>Dear editor<br><br>Thank you very much for your crucial comments for our manuscript entitled “The genome draft of the coconut (Cocos nucifera)” (GIGA-D-17-00038). We have made some revisions to the ms based on your comments. We removed revision mode and

Powered by Editorial Manager® and ProduXion Manager® from Aries Systems Corporation

	upload the clean manuscript without any tracking of changes. Meanwhile, we cited the reference database "Xiao Y, Xu P, Fan H, Baudouin L, Xia W, Bocs S et al. Supporting data for The genome draft of coconut (Cocos nucifera). Gigascience Database. 2017" in the revised manuscript. We hope the revised version can meet the requirement of "GigaScience".<br><br>Sincerely yours,<br><br>Yaodong Yang<br><br>PHONE NUMBER: 0086-898-63330602<br>FAX NUMBER: 0086-898-63330673<br>EMAIL: yyang@catas.cn<br>POSTAL ADDRESS:<br>Coconuts Research Institute, Chinese Academy of Tropical Agricultural Sciences, 496 Wenqing Av., Wenchang, Hainan 571339, P.R.China
**Additional Information:**	
**Question**	**Response**
Are you submitting this manuscript to a special series or article collection?	No
**Experimental design and statistics**<br><br>Full details of the experimental design and statistical methods used should be given in the Methods section, as detailed in our Minimum Standards Reporting Checklist. Information essential to interpreting the data presented should be made available in the figure legends.<br><br>Have you included all the information requested in your manuscript?	Yes
**Resources**<br><br>A description of all resources used, including antibodies, cell lines, animals and software tools, with enough information to allow them to be uniquely identified, should be included in the Methods section. Authors are strongly encouraged to cite Research Resource Identifiers (RRIDs) for antibodies, model organisms and tools, where possible.<br><br>Have you included the information requested as detailed in our Minimum Standards Reporting Checklist?	Yes
**Availability of data and materials**<br><br>All datasets and code on which the conclusions of the paper rely must be either included in your submission or	Yes

Powered by Editorial Manager® and ProduXion Manager® from Aries Systems Corporation

deposited in publicly available repositories (where available and ethically appropriate), referencing such data using a unique identifier in the references and in the “Availability of Data and Materials” section of your manuscript.<br><br>Have you have met the above requirement as detailed in our Minimum Standards Reporting Checklist?	

Powered by Editorial Manager® and ProduXion Manager® from Aries Systems Corporation

Manuscript
Click here to download Manuscript Coconut_Manuscript_ED_final.doc

The genome draft of coconut (*Cocos nucifera*)

Yong Xiao[1]*, Pengwei Xu[3]*, Haikuo Fan[1]*, Luc Baudouin[4,5]*, Wei Xia[1], Stéphanie Bocs[4,5], Junyang Xu[3], Qiong Li[2], Anping Guo[2], Lixia Zhou[1], Jing Li[1], Yi Wu[1], Zilong Ma[2], Alix Armero[4,6], Auguste Emmanuel Issali[7], Na Liu[3], Ming Peng[2&], Yaodong Yang[1&]

[1]Hainan Key Laboratory of Tropical Oil Crops Biology/Coconut Research Institute, Chinese Academy of Tropical Agricultural Sciences, Wenchang, Hainan 571339, P.R.China

[2]Institute of Tropical Bioscience and Biotechnology, Chinese Academy of Tropical Agricultural Science, Haikou, Hainan 571101, P. R. China

[3]BGI-Shenzhen, Shenzhen 518083, China

[4]AGAP, Université de Montpellier, CIRAD, INRA, Montpellier Supagro, F-34398 Montpellier, France

[5]CIRAD , UMR AGAP, F-34398, Montpellier France

[6]Montpellier Supagro , UMR AGAP, F-34398, Montpellier France

[7]Station Cocotier Marc Delorme, Centre National De RechercheAgronomique (CNRA)07 B.P. 13, Port Bouet,Côte d'Ivoire

*The authors have equal contribution to the manuscript

[&]Corresponding author

Yong Xiao: xiaoyong1980@catas.cn

Pengwei Xu: xupengwei@genomics.cn

Haikuo Fan: vanheco@163.com

Luc Baudouin: luc.baudouin@cirad.fr

Wei Xia: saizjxiawei@hainu.edu.cn

Stéphanie Bocs: stephanie.sidibe-bocs@cirad.fr

Junyang Xu: xujy@genomics.cn

Qiong Li: liqiong4416@126.com

Anping Guo: gap211@126.com

Lixia Zhou: glzz_2009@163.com

Jing Li: lijing002x@catas.cn

Yi Wu: wuyi-scuta@163.com

Zilong Ma: mzl900@163.com

Alix Armero: alix.armero_villanueva@cirad.fr

Auguste Emmanuel Issali: issaliemma@yahoo.com

Na Liu: naliu@genomics.cn

Ming Peng: mmpeng_2000@yahoo.com

Yaodong Yang: yyang@catas.cn

Background

Coconut palm (*Cocos nucifera*, 2n = 32), a member of genus *Cocos* and family Arecaceae (Palmaceae), is an important tropical fruit and oil crop. Currently, coconut palm is cultivated in 93 countries, including Central and South America, East and West Africa, Southeast Asia and the Pacific island, with a total growth area of more than 12 million hectares (www.fao.org/faostat/en/). Coconut palm is generally classified into two main categories: "Tall" (flowering 8-10 years after planting) and "Dwarf" (flowering 4-6 years after planting), based on morphological characteristics and breeding habits. This Palmae species has a long growth period before reproductive years which hinders conventional breeding progress. In spite of initial successes, improvements made by conventional breeding have been very slow. In the present study, we obtained *de novo* sequences of *Cocos nucifera* genome: a major genomic resource which could be used to facilitate molecular breeding in *Cocos nucifera* and accelerating the breeding process in this important crop.

Findings

A total of 419.67 gigabases (Gb) of raw reads were generated by the Illumina HiSeq 2000 platform using a series of paired-end and mate-pair libraries, covering the predicted *Cocos nucifera* genome length (2.42Gb, variety "Hainan Tall") to an estimated 173.32× read depth. A total scaffold length of 2.20 Gb was generated (N50 ＝418 Kb), representing 90.91% of the genome. The coconut genome was predicted to harbor 28,039 protein-coding genes, which is less than in *Phoenix dactylifera* (PDK30 variety: 28,889), *Phoenix dactylifera* (DPV01 variety: 41,660) and *Elaeis guineensis* (34,802). BUSCO evaluation demonstrated the obtained scaffold sequences covered 90.8% of the coconut genome, and that the genome annotation was 74.1% complete. Genome annotation results revealed that 72.75% of the coconut genome was consisted of transposable elements. Of which long-terminal repeat retrotransposons elements (LTRs) accounted for the largest proportion (92.23%).

Comparative analysis of the antiporter gene family and ion channel gene families between *C. nucifera* and *Arabidopsis thaliana* indicated that significant gene expansion may occurred in coconut involving Na+/H+ antiporter, Carnitine/acylcarnitine translocase, Potassium-dependent sodium-calcium exchanger, and potassium channel genes.

Conclusions

Despite its agronomic importance, *C. nucifera* is still under-studied. In this report, we present a draft genome of *C. nucifera* and provide genomic information that will facilitate future functional genomics and molecular assisted breeding in this crop species.

Keywords: Coconut palm, genome, Assembly, Annotation

Data description

Background

Coconut palm (*Cocos nucifera*, 2n = 32), the only species in genus *Cocos* in the family *Arecaceae*, is a tropical oil crop and widely cultivated in tropical regions due to its extensive application in agriculture and industry. Coconut palm is thought to be originated from the Southwest and Western Pacific region (including the Malay Peninsula and Archipelago, New Guinea, and the Bismarck Archipelago). At present, this tropical tree crop is distributed across 93 tropical countries [1], including Central and South American, East and West African, Southeast Asia and the Pacific Islands, and is grown over 12 million hectares of land (www.fao.org/faostat/en/).

In China, coconut palm grows in the subtropical regions - Hainan and Yunnan provinces - as an economic and ornamental plant. Coconut palm is cultivated over approximately 43,000 hectares in Hainan, with the “Hainan Tall” (HAT) variety covering 36,000 hectares [2]. The HAT coconut needs eight to ten years to enter its reproductive stage and has a height of 20-30 meters with a medium to large sized nut. The HAT cultivar is highly tolerant to salt and drought stress, but sensitive to temperatures below 10 °C. Coconut palm can disseminate through ocean currents: floating nuts sprout and grow naturally upon washing up on beaches. The ability to adapt to a high salt environment is closely related to this dissemination feature and to these natural growth conditions. The morphological characteristics of the HAT cultivar are shown in Figure 1. Here, we present the genome sequence of the Hainan Tall coconut and an analysis of the antiporter and ion channel gene families,

relevant to salinity tolerance. As draft genome sequences of coconut relatives (e.g. *Elaeis guineensis* [3] and *Phoenix dactylifera* [4, 5]) have previously been reported, we also performed a comparative analysis between coconut and these relative species for genome assembly and annotation characteristics.

Data description

Sample collection and sequencing strategy

The genomic DNA was extracted from the spear leaf of the variety "Hainan Tall" coconut (*Cocos nucifera* L. Taxonomy ID: 13894; 19°33'3"N, 110°47'25" E) individual from the coconut garden of the Coconut Research Institute (Wenchang, Hainan province, China) by using the CTAB extraction method [6]. Subsequently, four paired-end (PE) libraries with insert sizes of 170 bp, 500 bp, 450 bp and 800 bp and five mate-pair (MP) libraries with insert sizes of 2 Kb, 5 Kb, 10 Kb, 20 Kb and 40 Kb were constructed using the standard procedure provided by Illumina (San Diego, USA). After library preparation and quality control of the DNA samples, template DNA fragments were hybridized to the surface of the flow cells on an Illumina HiSeq2000 sequencer and amplified to form clusters and then sequenced by following the standard Illumina manual. Finally, we generated 714.67 Gb of raw reads from all constructed libraries. The raw outputs for each sequenced library are summarized in Table 1. Before assembly, the raw reads were pretreated using the following stringent filtering processes via the SOAPfilter (v2.2) [7] software: (1) removed reads with 25% low-quality bases (quality scores ≤ 7); (2) removed reads with N bases more than 1%; (3) discarded reads with adapter contamination and/or PCR duplicates; (4) removed reads with undersized insert sizes. Finally, 419.08 Gb (estimated 173.17× read depth) of high-quality sequences were obtained for genome assembly.

De novo assembly of short reads of *Cocos nucifera*

We used 209.38 Gb clean reads of the short-insert libraries (excluding the 450bp library) to estimate the coconut genome size by k-mer frequency distribution analysis [7]. The genome size (G) of *Cocos nucifera* could be estimated by the following formula:

$$G = N \times (L - K + 1) / K\_depth$$

where N represents the total of number of reads, L represents the read length, K represents the k-mer value used in the analysis and K_depth refers to the main peak in the k-mer distribution curve. In our

calculations, N was 2,049,520,223, L was 100 and K_depth was 71 for K=17. As a result, *Cocos nucifera* genome was estimated to be 2.42 gigabases (Gb). K-mer size distribution analysis (Figure 2) indicated that *Cocos nucifera* was a diploid species with low heterozygosity and a high proportion of repetitive sequences.

We then assembled the *Cocos nucifera* genome using the software SOAPdenovo2 (SOAPdenovo2, RRID:SCR_014986) in three steps: contig construction, scaffold construction and gap filling. In the contig construction step: the SOAPdenovo2 was run with the parameters 'pregraph -K 63 -R -d 1' to construct de Bruijn graphs from paired-end libraries with insert sizes ranging from 170 to 800 bp. The k-mers from the de Bruijn graphs were then used to form contiguous sequences (contigs) with the parameters 'contig -R' by clipping tips, merging bubbles and removing low coverage links. In the scaffold construction step: the orders of the contigs were determined by using paired-end and mate-pair information with parameters 'map -k 43' and 'scaff -F -u'. In more detail, SOAPdenovo2 maps the reads from paired-end and mate pair libraries to contigs based on a hash table (keys are unique k-mers on contigs; values are positions). In such cases, two contigs are considered to be linked if the bridging of the contigs are supported by five paired-end read pairs or three mate-pair read pairs. In the gap filling step: gaps within scaffolds were filled by utilizing KGF [7] v1.06 and GapCloser v1.12-r6 (GapCloser, RRID:SCR_015026) [7] with paired-end libraries (having an insert size from 170 to 800 bp in cases, where one end could be mapped to one contig and the other end extended into a gap). To optimize the assembled sequence, Rabbit (a Poisson-based k-mer model software [8]) was used to remove the redundant sequences. A final length of 2.20 Gb for the scaffolds was obtained and used for further analysis, accounting for 90.91% of the predicted genome size and larger than the African oil palm and datepalm genomes (Table 2). Meanwhile, the N50 of the obtained contigs was 72.64 Kb and 418.06 Kb for the scaffolds which have excluding scaffolds less than 100 bp. The comparison of N50 values for the assembled coconut genome and for four previously published palm genomes *Elaeis guineensis* [3], *Elaeis oleifera* [3], *Phoenix dactylifera* (PDK30) [4] and *Phoenix dactylifera*(DPV01) [5] is listed in Table 2.

Genome evaluation

The 57,304 unigenes (transcript obtained from three different tissues, spear leaves, young leaves and fruit flesh) as previously reported by Fan et al. [9] were aligned to the assembled genome of *Cocos nucifera* using BLAT (BLAT, RRID:SCR_011919) [10] with default parameters. The alignment

results indicated that the assembled genome of *Cocos nucifera* covered 96.78% of the expressed unigenes, suggesting a high level of coverage has been reached for the assembled genome (Table 3).

We also evaluated the level of genome completeness for the assembled sequences by using BUSCO v2.0 (BUSCO, RRID:SCR_015008) [11], which quantitatively assesses genome completeness using evolutionarily-informed expectations of gene content from near-universal single-copy orthologs selected from OrthoDB v9 (OrthoDB, RRID:SCR_011980; http://busco.ezlab.org/, plant set). BUSCO analysis showed that there are separate 90.8% and 3.4% of the 1,440 expected plant genes were identified as complete and fragmented genes respectively, while 5.8% of genes were considered to be missing from the assembled coconut genome sequence. The comparative results of the BUSCO estimation in coconut and in the four other palm genome sequences indicates that the smallest fraction of missing genes as predicted by BUSCO was found in the coconut genome assembly (Table 4).

Repeat annotation

We combined homology - based annotation and *de novo* method to identify transposable elements (TEs) and the tandem repeats in the *Cocos nucifera* genome. In homology - based annotation step: TEs were identified by searching against the Repbase library (version 20.04) [12] with RepeatMasker (RepeatMasker, RRID:SCR_012954) (v4.0.5) [13] and RepeatProteinMasker (v4.0.5) [13]. In the *de novo* step: *de novo* libraries were constructed based on the genome sequences using the *de novo* prediction program RepeatModeler (RepeatModeler, RRID:SCR_015027) and LTR_FINDER (LTR_FINDER, RRID:SCR_015247) [14] by removing contaminant and multi-copy genes. Subsequently, novel transposable elements were identified and classified using RepeatMasker. Tandem repeat sequences were identified by TRF (Tandem Repeat Finder) software [15] with the following parameters ‘Match = 2, Mismatch = 7, Delta = 7, PM = 80, PI = 10, Minscore = 50 and MaxPerid = 2000’. The total length of the tandem repeat sequences predicted by the software was 151,229,585 bp, comprising 6.86% of the coconut genome. Finally, 1.6 Gb of non-redundant repetitive elements were identified, accounting for 74.48% of the coconut genome. Transposable elements took up 72.75% of the total 1.6Gb of repetitive elements with the long-terminal repeat retrotransposon (LTR) class accounting for 92.23% of all TEs and 67.1% of the coconut genome (Table 5).

Gene prediction

We combined three strategies to predict genes in *Cocos nucifera* genome: homology - based, *de novo* and transcript alignment. For homology - based annotation: the protein sequences of *Arabidopsis thaliana* [16], *Oryza sativa*[17], *Sorghum bicolor* [18], *Zea mays* [19], *Elaeis guineensis*, and *Phoenix dactylifera* (DPV01) were downloaded from each corresponding source (see "Availability of data sources"). The coconut genome was aligned against these downloaded databases using TBLASTN[20] with parameter '-e 1e-5 -F -m 8' and BLAST results were processed by solar (v0.9) with parameter '-aprot 2 genome2 -z' to determine the candidate gene loci. Next, we extracted the genomic sequences of candidate gene loci along with 1kb flanking sequences, and applied GeneWise 2.2.0 (GeneWise, RRID:SCR_015054) [21] to define the intron - exon boundaries. The genes with pre-stop codon or frame-shifts were excluded from further analysis.

For *de novo* prediction: we randomly selected 1000 full-length genes (GeneWise score equal 100, intact structure: start codon, stop codon, perfect intron-exon boundary) from gene models predicted by homology-based methods to train the model parameters for AUGUSTUS 2.5 (Augustus: Gene Prediction, RRID:SCR_008417) [22]. Two software programs, AUGUSTUS 2.5 and GENSCAN (GENSCAN, RRID:SCR_012902) 1.0 [23], were used to do *de novo* prediction on the repeat-masked genome of *Cocos nucifera*. Genes with incomplete structure or protein coding length less than 150bp were filtered out.

Subsequently, genes from both homology-based and *de novo* methods were combined to obtain non-redundant gene sets by using GLEAN [24] with the following parameters: minimum coding sequence length 150 bp and maximum intron length 50 kb. Genes were filtered with the same thresholds as were used for homology-based annotation.

For transcriptome-based prediction: RNA-seq data (SRR606452) as previously reported by Fan et al. [9] was mapped onto the coconut genome to identify the splice junctions using the software TopHat v2.1.1 (TopHat, RRID:SCR_013035) [25]. The software Cufflinks v2.2.1 (Cufflinks, RRID:SCR_014597) [26] was then used to assemble transcripts with the aligned reads. The coding potential of these transcripts was identified using a fifth-order Hidden Markov Model, which was estimated with the same gene sets used in AUGUSTUS training by train GlimmerHMM, an application in the GlimmerHMM package (GlimmerHMM, RRID:SCR_002654) [27]. The transcripts with intact open reading frames (ORFs) were extracted and the longest transcript was retrieved as a representative of a gene whiles multiple transcripts from on a same locus.

Finally, we merged the GLEAN and the transcriptome result to form a comprehensive gene set using an in-house annotation pipeline with the following steps: firstly, all-to-all BLASTP analysis of protein sequences was performed between GLEAN results and transcript assemblies with an E-value cutoff of 1e-10. These transcript assemblies were added to the GLEAN result to form (untranslated region) UTRs or alternative splicing products, depending on whether the coverage and identity of the alignment results reached 0.9 or not. If the transcript assemblies had no BLAST hit with the GLEAN results, these transcript assemblies were added to the final gene set as novel gene. The protocol for integrating GLEAN and transcriptome data is shown in Figure 3.

Gene evaluation

The annotation processes identified 28,039 protein-coding genes (Table 2), which is less than the predicted gene numbers of *Phoenix dactylifera* (PDK30,28,889), *Phoenix dactylifera* (DPV01, 41,660) and *Elaeis guineensis* (34,802). Meanwhile, the BUSCO evaluation showed that 74.1% and 11.2% of 1,440 expected plant genes were identified as complete and fragmented, with 14.7% of genes considered missing in the gene sets. The BUSCO results showed that our gene prediction was more complete than that of *Phoenix dactylifera* (PDK30) and *Elaeis guineensis*, but less complete than that of *Phoenix dactylifera* (DPV01) (Table 6),

Gene Function

Gene function annotation was done based on sequence similarity and domains conservation. Firstly, the coconut protein coding genes were aligned against the KEGG (KEGG, RRID:SCR_012773) protein databases [28], SwissProt and TrEMBL [29] using BLASTP at a cut-off E-value threshold of 10^{-5}. Subsequently, the best match from the alignment was used to represent the gene function. We obtained 18,445 KEGG, 18,867 Swissprot and 24,882 Tremble annotated genes. Secondly, InterProScan (InterProScan, RRID:SCR_005829) 5.11-51.0 software [30] was employed to identify the motif and domain based on the public databases Pfam (Pfam, RRID:SCR_004726) [31], PRINTS (PRINTS, RRID:SCR_003412) [32], ProDom (ProDom, RRID:SCR_006969) [33], SMART (SMART, RRID:SCR_005026) [34], PANTHER (PANTHER, RRID:SCR_004869) [35], TIGRFAM (JCVI TIGRFAMS, RRID:SCR_005493) [36] and SUPERFAMILY (SUPERFAMILY, RRID:SCR_007952) [37]. The gene function annotation demonstrated that 21,087 of the coconut proteins had conserved motifs and 1,622 Gene Ontology (GO) terms were assigned to 15,705 coconut proteins from the corresponding InterPro (InterPro, RRID:SCR_006695) entry [38]. In total,

approximately 89.41% of these genes were functionally annotated using the above methods.

Gene Family Construction

Protein sequences of thirteen angiosperms, including *Elaeis guineensis*, *Phoenix dactylifera* (DPV01), *Sorghum bicolor*, *Prunus persica*, *Solanum tuberosum*, *Glycine max*, *Arabidopsis thaliana*, *Theobroma cacao*, *Vitis vinifera*, *Musa acuminata*, *Carica papaya*, *Populus trichocarpa* and *Amborella trichopoda*, were download from each corresponding ftp site (see "Availability of data sources"). For genes with alternative splicing variants, the longest transcripts were selected to represent the gene. The gene numbers of *Elaeis guineensis* and *Phoenix dactylifera* (DPV01) were greatly different from the research paper published in 2013[3, 5], because genes of these two species were re-predicted using the NCBI Prokaryotic Genome Annotation Pipeline which seemed to be more reasonable. Similarities between paired sequences were calculated using BLASTP with an E-value threshold of 1e-5. OrthoMCL (OrthoMCL DB: Ortholog Groups of Protein Sequences, RRID:SCR_007839) [39] was used to identify gene family based on the similarities of the genes and a Markov Chain Clustering (MCL) with default parameters. About 79.80% of *Cocos nucifera* genes were assigned to 14,411 families, of which 282 families only existed in *Cocos nucifera* (coconut specific families) (Table 7). Figure 4 shows the shared gene families for orthologous genes. There are 544 orthologous families shared by five monocot species and 7706 orthologous families shared by all monocot and dicot species, suggesting 544 monocot unique functions shared by five monocot species and 7,706 ancestral functions in the most recent common ancestor of the angiosperms.

Phylogenetic analysis

We extracted 247 single copy orthologous genes derived from the gene family analysis step, and then aligned the protein sequences of each family with MUSCLE (MUSCLE, RRID:SCR_011812) (v3.8.31) [40]. Next, the protein alignments were converted to corresponding coding sequences (CDS) using an in-house Perl script. These coding sequences of each single copy gene family were concatenated to form one super gene for each species. The nucleotides at position 2 (phase one site) and 3 (four degenerate site) of codon were extracted separately to construct the phylogenetic tree by PhyML 3.0 (PhyML, RRID:SCR_014629) [41] using a HKY85 substitution model and a gamma distribution across sites. The tree constructed by phase one sites was consistent with the tree constructed by four degenerate sites.

Divergence time

The Bayesian relaxed molecular clock approach was used to estimate species divergence time using MCMCTREE in PAML (PAML, RRID:SCR_014932) [42], based on the four-degenerate sites and the data set used in phylogenetic analysis, with previously published calibration times [43] (divergence between *Arabidopsis thaliana* and *Carica papaya* was 54-90 Mya, divergence between *Arabidopsis thaliana* and *Populus trichocarpa* was 100-120 Mya). The divergence time between coconut and oil palm is about 46.0 (25.4-83.3) million years ago (Figure 5), which is less than the divergence time between coconut and date palm.

Identification of antiporter genes in coconut genome

Antiporters are transmembrane proteins involved in the exchange of substances within and outside the membrane. In Arabidopsis, the functions of antiporter genes have been well characterized experimentally, and this gene family was subdivided into thirteen different functional groups. Among them, three functional clusters involved in Na+/H+ antiporters, some of which were documented to be associated with salt tolerance [44, 45].

The amino acid sequences of 70 *antiporter* genes of Arabidopsis were downloaded from the Arabidopsis Information Resource TAIR website (TAIR, RRID:SCR_004618; http://www.arabidopsis.org) and used as queries for BLASTP against the predicted proteins in the *Cocos nucifera* genome with a cut-off e-value of 1e-10. A total of 126 *antiporter* genes were identified in coconut genome. Using local Hidden Markov Model-based HMMER (v3.0) searches and the Pfam database, seven antiporter genes were excluded from further analysis because of the lack of conserved domain. The detailed information of the 119 antiporter genes is listed in Additional file 1.

In order to elucidate the evolutionary relationship and potential functions of the antiporters identified in the study, we applied phylogenetic analysis of *Arabidopsis* and *C. nucifera* antiporter proteins using the neighbor joining method (Figure 6). Phylogenetic analysis showed that the 119 antiporter genes from *C. nucifera* can be subdivided into twelve groups and that almost all antiporter genes were clustered together with the functional groups in *Arabidopsis thaliana*.

Phylogenetic analysis showed that the number of antiporter genes was equal between *Arabidopsis thaliana* and *C. nucifera* for most groups except for G1 (one of three Na+/H+ antiporter family), G3 (carnitine/acylcarnitine translocase family) and G12 (potassium-dependent

sodium-calcium exchanger). The G1 group (one of three Na+/H+ antiporter families) contained only one Arabidopsis antiporter gene and but 14 *C. nucifera* antiporters (1-At/14-Cn), whereas G3 (carnitine/acylcarnitine translocase family) contained 1-At/29-Cn, and G13 (Potassium-dependent sodium-calcium exchanger) contained 3-At/11-Cn. The Na+/H+ antiporter family had been reported to be associated with salt stress. The expansion of the Na+/H+ antiporter gene family in coconut palm maybe associated with the high salt tolerance of coconut. Meanwhile, carnitine/acylcarnitine translocase is involved in fatty acid transport cross the mitochondrial membranes. This gene family expansion maybe associated with accumulation of fatty acid in coconut pulp. Moreover, coconut water contains a high density of potassium ion, approximately 312 mg potassium ion per 100 g coconut water [46]. In this study, the gene number of potassium-dependent sodium-calcium exchangers were also detected to be significantly increased compared to Arabidopsis.

Identification of ion channel genes in coconut genome

A total of 67 ion channel genes were identified in the coconut genome (Additional file 2). The amino acid sequences of 67 *C. nucifera* and 60 *Arabidopsis* ion channel genes were used to analyze their evolutionary relationship (Figure 7). Almost all ion channel genes from *C. nucifera* can be clustered into the function groups found in *Arabidopsis thaliana*. The number of ion channel genes was equal between *Arabidopsis thaliana* and *Cocos nucifera* in most groups except for G5 (potassium channel). Many more genes (21) from *C. nucifera* than from *Arabidopsis thaliana* (9 genes) were present in group 5 (potassium channels). The gene family expansion maybe associated with accumulation of potassium ions in coconut water.

Conclusion

Cocos nucifera (2n = 32) is an important tropical crop, and is also used as an ornamental plant in the tropics. In the present study, we sequenced and *de novo* assembled the coconut genome. A total scaffold length of 2.2 Gb was generated, with scaffold N50 of 418 Kb. The divergence time of *Cocos nucifera* and *Elaeis guineensis* is more recent than that of *Cocos nucifera* and *Phoenix dactylifera*, suggesting a closer relationship between *C. nucifera* and *E. guineensis*. Comparative analysis of antiporter and ion channels between *C. nucifera* and *Arabidopsis thaliana* showed significant gene family expansions maybe involving Na+/H+ antiporters, carnitine/acylcarnitine translocases, potassium-dependent sodium-calcium exchangers, and potassium channels. The expansion of these gene families may be associated with adaptation to salt stress, accumulation of fatty acid in coconut

pulp and potassium ions in coconut water. The data output of the coconut genome will provide a valuable resource and reference information for the development of high density molecular makers, construction of high density linkage maps, detection of QTL (quantitative trait loci), genome-wide association mapping, and molecular breeding.

Availability of supporting data

Supporting data are available in the GigaDB database (GigaDB, RRID:SCR_004002) [47]. Raw data were deposited in the Sequence Read Archive (SRA539146) with the project accession code PRJNA374600 for the *Cocos nucifera* genome. Previously published RNA-seq data used for transcriptome-based prediction is available under accession number SRR606452.

Availability of other angiosperms data sources

Arabidopsis thaliana, *Oryza sativa*, *Sorghum bicolor*, *Zea mays*, *Sorghum bicolor, Solanum tuberosum, Prunus persica*, *Theobroma cacao*, *Vitis vinifera*, *Musa acuminata*, *Carica papaya*, *Populus trichocarpa*, *Amborella trichopoda*: https://phytozome.jgi.doe.gov/pz/portal.html (phytozomev9.1)

Elaeis guineensis: ftp://ftp.ncbi.nlm.nih.gov/genomes/all/GCF/000/442/705/GCF_000442705.1_EG5/

Phoenix dactylifera (DPV01):

ftp://ftp.ncbi.nlm.nih.gov/genomes/all/GCF/000/413/155/GCF_000413155.1_DPV01/

Phoenix dactylifera (PDK30):

http://qatar-weill.cornell.edu/research/datepalmGenome/download.html

Competing interests

The authors declare that they have no competing interests.

Funding

This study was supported by International Science and Technology Cooperation projects of Hainan Province (No. KJHZ2014-24), Hainan Natural Science Foundation (No. 313058), the major Technology Project of Hainan (No. ZDZX2013023-1), the fundamental Scientific Research Funds for Chinese Academy of Tropical Agriculture Sciences (CATAS-No. 1630032012044, 1630052014002, 1630052015050, 1630152017019, and 1630152016006), Central Public-interest Scientific Institution Basal Research Fund for Innovative Research Team Program of CATAS (No.17CXTD-28).

Author's contribution

YX, HF, YY, MP, QL, AG designed the study and contribute to the project coordination; XY, PX, WX wrote the paper; LZ, JL, YW collected the samples and extracted the genomic DNA; YX, BL, BS, JX, AA, EI, NL conducted the genome analyses.

Acknowledgements

Annaliese S. Mason is gratefully acknowledged for assistance with language editing and manuscript revisions.

References

1. Batugal P, V Ramanatha Rao and J Oliver, editors. Coconut Genetic Resources. International Plant Genetic Resources Institute – Regional Office for Asia, the Pacific and Oceania (IPGRI-APO) Serdang, Selangor DE, Malaysia; 2005.
2. Tang B, Tang M, Chen C, Qiu P, Liu Q, Wang M, et al. Characteristics of soil fauna community in the Dongjiao coconut plantation ecosystem in Hainan, China. Acta Ecologica Sinica. 2006;26(1):26-32. doi:http://dx.doi.org/10.1016/S1872-2032(06)60003-6.
3. Singh R, Ong-Abdullah M, Low ET, Manaf MA, Rosli R, Nookiah R, et al. Oil palm genome sequence reveals divergence of interfertile species in Old and New worlds. Nature. 2013;500(7462):335-9. doi:10.1038/nature12309.
4. Al-Dous EK, George B, Al-Mahmoud ME, Al-Jaber MY, Wang H, Salameh YM, et al. De novo genome sequencing and comparative genomics of date palm (Phoenix dactylifera). Nature biotechnology. 2011;29(6):521-7. doi:10.1038/nbt.1860.
5. Al-Mssallem IS, Hu S, Zhang X, Lin Q, Liu W, Tan J, et al. Genome sequence of the date palm Phoenix dactylifera L. Nature communications. 2013;4:2274. doi:10.1038/ncomms3274.
6. Murray MG and Thompson WF. Rapid isolation of high molecular weight plant DNA. Nucleic acids research. 1980;8(19):4321-5.
7. Luo R, Liu B, Xie Y, Li Z, Huang W, Yuan J, et al. SOAPdenovo2: an empirically improved memory-efficient short-read de novo assembler. Gigascience. 2012;1(1):18. doi:10.1186/2047-217X-1-18.
8. Zhan, D. Rabbit Genome Assembler. 2017. https://github.com/gigascience/rabbit-genome-assembler
9. Fan H, Xiao Y, Yang Y, Xia W, Mason AS, Xia Z, et al. RNA-Seq analysis of Cocos nucifera: transcriptome sequencing and de novo assembly for subsequent functional genomics approaches. PloS one. 2013;8(3):e59997. doi:10.1371/journal.pone.0059997.
10. Kent WJ. BLAT--the BLAST-like alignment tool. Genome Research. 2002;12(4):656-64. doi:10.1101/gr.229202. .
11. Simao FA, Waterhouse RM, Ioannidis P, Kriventseva EV and Zdobnov EM. BUSCO: assessing genome assembly and annotation completeness with single-copy orthologs. Bioinformatics (Oxford, England). 2015;31(19):3210-2. doi:10.1093/bioinformatics/btv351.
12. Jurka J, Kapitonov VV, Pavlicek A, Klonowski P, Kohany O and Walichiewicz J. Repbase Update, a database of eukaryotic repetitive elements. Cytogenetic and genome research. 2005;110(1-4):462-7. doi:10.1159/000084979.

13. Tarailo-Graovac M and Chen N. Using RepeatMasker to identify repetitive elements in genomic sequences. Current protocols in bioinformatics. 2009;Chapter 4:Unit 4.10. doi:10.1002/0471250953.bi0410s25.

14. Xu Z and Wang H. LTR_FINDER: an efficient tool for the prediction of full-length LTR retrotransposons. Nucleic acids research. 2007;35(Web Server issue):W265-8. doi:10.1093/nar/gkm286.

15. Benson G. Tandem repeats finder: a program to analyze DNA sequences. Nucleic acids research. 1999;27(2):573-80.

16. The Arabidopsis Genome Initiative, Analysis of the genome sequence of the flowering plant Arabidopsis thaliana. Nature. 2000;408(6814):796-815. doi:http://www.nature.com/nature/journal/v408/n6814/suppinfo/408796a0_S1.html.

17. Goff SA, Ricke D, Lan TH, Presting G, Wang R, Dunn M, et al. A draft sequence of the rice genome (Oryza sativa L. ssp. japonica). Science (New York, NY). 2002;296(5565):92-100. doi:10.1126/science.1068275.

18. Paterson AH, Bowers JE, Bruggmann R, Dubchak I, Grimwood J, Gundlach H, et al. The Sorghum bicolor genome and the diversification of grasses. Nature. 2009;457(7229):551-6. doi:10.1038/nature07723.

19. Schnable PS, Ware D, Fulton RS, Stein JC, Wei F, Pasternak S, et al. The B73 maize genome: complexity, diversity, and dynamics. Science (New York, NY). 2009;326(5956):1112-5. doi:10.1126/science.1178534.

20. Altschul SF, Madden TL, Schaffer AA, Zhang J, Zhang Z, Miller W, et al. Gapped BLAST and PSI-BLAST: a new generation of protein database search programs. Nucleic acids research. 1997;25(17):3389-402.

21. Birney E, Clamp M and Durbin R. GeneWise and Genomewise. Genome Research. 2004;14(5):988-95. doi:10.1101/gr.1865504.

22. Stanke M, Keller O, Gunduz I, Hayes A, Waack S and Morgenstern B. AUGUSTUS: ab initio prediction of alternative transcripts. Nucleic acids research. 2006;34(Web Server issue):W435-9. doi:10.1093/nar/gkl200.

23. Burge C and Karlin S. Prediction of complete gene structures in human genomic DNA. Journal of molecular biology. 1997;268(1):78-94. doi:10.1006/jmbi.1997.0951.

24. Elsik CG, Mackey AJ, Reese JT, Milshina NV, Roos DS and Weinstock GM. Creating a honey bee consensus gene set. Genome biology. 2007;8(1):R13. doi:10.1186/gb-2007-8-1-r13.

25. Trapnell C, Pachter L and Salzberg SL. TopHat: discovering splice junctions with RNA-Seq. Bioinformatics (Oxford, England). 2009;25(9):1105-11. doi:10.1093/bioinformatics/btp120.

26. Trapnell C, Williams BA, Pertea G, Mortazavi A, Kwan G, van Baren MJ, et al. Transcript assembly and quantification by RNA-Seq reveals unannotated transcripts and isoform switching during cell differentiation. Nature biotechnology. 2010;28(5):511-5. doi:10.1038/nbt.1621.

27. Majoros WH, Pertea M and Salzberg SL. TigrScan and GlimmerHMM: two open source ab initio eukaryotic gene-finders. Bioinformatics (Oxford, England). 2004;20(16):2878-9. doi:10.1093/bioinformatics/bth315.

28. Ogata H, Goto S, Sato K, Fujibuchi W, Bono H and Kanehisa M. KEGG: Kyoto Encyclopedia of Genes and Genomes. Nucleic acids research. 1999;27(1):29-34.

29. Bairoch A and Apweiler R. The SWISS-PROT protein sequence database and its supplement

TrEMBL in 2000. Nucleic acids research. 2000;28(1):45-8.

30. Jones P, Binns D, Chang HY, Fraser M, Li W, McAnulla C, et al. InterProScan 5: genome-scale protein function classification. Bioinformatics (Oxford, England). 2014;30(9):1236-40. doi:10.1093/bioinformatics/btu031.

31. Bateman A, Birney E, Durbin R, Eddy SR, Howe KL and Sonnhammer EL. The Pfam protein families database. Nucleic acids research. 2000;28(1):263-6.

32. Attwood TK, Croning MDR, Flower DR, Lewis AP, Mabey JE, Scordis P, et al. PRINTS-S: the database formerly known as PRINTS. Nucleic acids research. 2000;28(1):225-7.

33. Corpet F, Gouzy J and Kahn D. Recent improvements of the ProDom database of protein domain families. Nucleic acids research. 1999;27(1):263-7.

34 Schultz J, Copley RR, Doerks T, Ponting CP and Bork P. SMART: a web-based tool for the study of genetically mobile domains. Nucleic acids research. 2000;28(1):231-4.

35. Mi H, Huang X, Muruganujan A, Tang H, Mills C, Kang D, et al. PANTHER version 11: expanded annotation data from Gene Ontology and Reactome pathways, and data analysis tool enhancements. Nucleic acids research. 2017;45(Database issue):D183-D9. doi:10.1093/nar/gkw1138.

36. Selengut JD, Haft DH, Davidsen T, Ganapathy A, Gwinn-Giglio M, Nelson WC, et al. TIGRFAMs and Genome Properties: tools for the assignment of molecular function and biological process in prokaryotic genomes. Nucleic acids research. 2007;35(Database issue):D260-D4. doi:10.1093/nar/gkl1043.

37. Wilson D, Pethica R, Zhou Y, Talbot C, Vogel C, Madera M, et al. SUPERFAMILY--sophisticated comparative genomics, data mining, visualization and phylogeny. Nucleic acids research. 2009;37(Database issue):D380-6. doi:10.1093/nar/gkn762.

38. Burge S, Kelly E, Lonsdale D, Mutowo-Muellenet P, McAnulla C, Mitchell A, et al. Manual GO annotation of predictive protein signatures: the InterPro approach to GO curation. Database : the journal of biological databases and curation. 2012;2012:bar068. doi:10.1093/database/bar068.

39. Li L, Stoeckert CJ, Jr. and Roos DS. OrthoMCL: identification of ortholog groups for eukaryotic genomes. Genome Research. 2003;13(9):2178-89. doi:10.1101/gr.1224503.

40. Edgar RC. MUSCLE: multiple sequence alignment with high accuracy and high throughput. Nucleic acids research. 2004;32(5):1792-7. doi:10.1093/nar/gkh340.

41. Guindon S, Dufayard JF, Lefort V, Anisimova M, Hordijk W and Gascuel O. New algorithms and methods to estimate maximum-likelihood phylogenies: assessing the performance of PhyML 3.0. Systematic biology. 2010;59(3):307-21. doi:10.1093/sysbio/syq010.

42. Yang Z. PAML 4: phylogenetic analysis by maximum likelihood. Molecular biology and evolution. 2007;24(8):1586-91. doi:10.1093/molbev/msm088.

43. Tuskan GA, Difazio S, Jansson S, Bohlmann J, Grigoriev I, Hellsten U, et al. The genome of black cottonwood, Populus trichocarpa (Torr. & Gray). Science (New York, NY). 2006;313(5793):1596-604. doi:10.1126/science.1128691.

44. Shi H, Lee BH, Wu SJ and Zhu JK. Overexpression of a plasma membrane Na+/H+ antiporter gene improves salt tolerance in Arabidopsis thaliana. Nature biotechnology. 2003;21(1):81-5. doi:10.1038/nbt766.

45. Brini F, Hanin M, Mezghani I, Berkowitz GA and Masmoudi K. Overexpression of wheat Na+/H+ antiporter TNHX1 and H+-pyrophosphatase TVP1 improve salt- and drought-stress

tolerance in Arabidopsis thaliana plants. Journal of experimental botany. 2007;58(2):301-8. doi:10.1093/jxb/erl251.

46. Yong JW, Ge L, Ng YF and Tan SN. The chemical composition and biological properties of coconut (Cocos nucifera L.) water. Molecules (Basel, Switzerland). 2009;14(12):5144-64. doi:10.3390/molecules14125144.

47. Xiao Y, Xu P, Fan H, Baudouin L, Xia W, Bocs S et al. Supporting data for "The genome draft of coconut (*Cocos nucifera*)". *Gigascience Database*. 2017. http://dx.doi.org/10.5524/100347

Tables

Table 1 Data outputs produced by sequencing different insert size libraries

Library type	Lane	Reads Length(bp)	Insert Size(bp)	Raw data (Gb)	Clean data(Gb)
PE101	3	100	170	128.75(53.20)	111.32(46)
PE251	2	250	450	73.86(30.52)	56.42(23.31)
PE101	2	100	500	64(26.45)	65.11(26.90)
PE101	2	100	800	78.16(32.30)	64.90(26.82)
MP50	3	49	2000	128.6(53.14)	60.70(25.08)
MP50	2	49	5000	71.75(29.65)	18.62(7.69)
MP50	2	49	10000	74.65(30.85)	18.53(7.66)
MP50	2	49	20000	70.7(29.21)	19.35(7.99)
MP50	1	49	40000	24.2(10.08)	4.13(1.71)
Total	19			714.67(295.32)	419.08(173.17)

Note: The sequencing depth was shown in parentheses, calculated based on a genome size of 2.42G. Clean data were obtained by filtering raw data with low-quality and duplicate reads. PE: paired-end, MP: mate pair.

Table 2 Comparison analysis of genome sizes, assembly and annotation of four palmae species, including coconut, *Phoenix dacylifera* (PDK30 and DPV01, two different versions), *Elaeis guineensis* (EG), and *Elaeis oleifera* (EO)

Species	Sequencing technology	Sequence coverage	Estimated size(Gb)	Assembly size(Gb)	Contig N50(Kb)	Scaffold N50(Kb)	Gene Number	TEs percent (%)
Phoenix dactylifera (PDK30)	Illumina GAIIx	53.4x	0.66	0.38	6.44	30.48	28,889	23.6
Phoenix dactylifera (DPV01)	454,SOLiD, ABI3730	139x	0.67	0.56	10.81	334.08	41,660	38.87
Elaeis guineensis (African oil palm)	454	16X	1.8	1.54	9.37	1045.41	34,802	43.24
Elaeis oleifera (American oil palm)	454	16x	1.8	1.40	8.45	333.11	--	--
Cocos nucifera (Hai nan Tall)	Illumina HiSeq	173X	2.42	2.20	72.64	418.07	28,039	72.75

Note: Coconut: *Cocos nucifera* (Hainan Tall); PDK30: *Phoenix dactylifera* (PDK30); DPV01: *Phoenix*

dactylifera (DPV01); EG: *Elaeis guineensis* (Africanoil palm E5 build); EO *Elaeis oleifera* (American oil palm, O8-build); TE results were obtained using the same pipeline as for the coconut genome

Table 3 The gene coverage of *Cocos nucifera* based on transcriptome data

Dataset	Number	Total length (bp)	Base coverage by assembly	Sequencecoverage by assembly (%)
All	57,304	43,090,665	96.78	99.57
>200bp	57,304	43,090,665	96.78	99.57
>500bp	25,713	33,470,388	96.36	99.85
>1000bp	13,796	25,004,919	95.99	99.94

Table 4 The comparative analysis of assembly results of five palm species with BUSCO software, including coconut, *Phoenix dacylifera* (PDK30 and DPV01, two varieties), *Elaeis guineensis* (EG), and *Elaeis oleifera* (EO)

	Coconut		PDK30		DPV01		EG		EO	
BUSCOs	N	P (%)	N	P (%)	N	P (%)	N	P(%)	N	P(%)
Total	1440		1440		1440		1440		1440	
Complete single-copy	1192	82.8	1042	72.4	1160	80.6	1100	76.4	1004	69.7
Complete duplicated	115	8.0	81	5.6	134	9.3	116	8.1	63	4.4
Fragment	49	3.4	98	6.8	42	2.9	60	4.2	84	5.8
Missing	84	5.8	219	15.2	104	7.2	164	11.3	289	20.1

Note: Coconut: *Cocos nucifera* (the Hainan Tall); PDK30: *Phoenix dactylifera* (PDK30); DPV01:*Phoenix dactylifera* (DPV01); EG: *Elaeis guineensis*(African oil palm E5 build); EO *Elaeis oleifera* (American oil palm, O8-build);

Table 5 Classification of predicted transposable elements in the coconut genome

	Repabse TEs length	Protein TEs length	*De novo* TEs length	Combined TEs length	percentage
DNA	20,936,158	24,655,089	35,131,002	58,119,982	2.64
LINE	4,251,185	9,631,472	7,610,172	19,197,064	0.87
SINE	85,717	0.00	186,364	270,055	0.012
LTR	361,968,154	512,700,933	1,419,281,798	1,478,182,089	67.10
Other	8,145	0.00	0.00	8,145	0.0004
Unknown	0.00	12,360	139,084,335	139,096,695	6.31

Total	385,037,442	546,965,774	1,552,582,881	1,602,630,396	72.75

Note: Repabse TEs means RepeatMask against Repbase; Protein TEs means RepeatProteinMask result against Repbase protein; *De novo* TEs means RepeatMask against the *de novo* library; Combined TEs: the combined results of these three steps.

Table 6 The comparative analysis of gene prediction results of four palm species with BUSCO software

	Coconut		PDK30		DPV01		EG	
BUSCOs	N	P (%)	N	P (%)	N	P (%)	N	P (%)
Total	1440		1440		1440		1440	
Complete single-copy	965	74.1	748	51.9	1195	83.0	555	38.5
Complete duplicated	102	7.1	81	5.6	159	11.0	53	3.7
Fragment	162	11.2	255	17.7	44	3.1	270	18.8
Missing	211	14.7	356	24.8	42	2.9	562	39.0

Note: Coconut: *Cocos nucifera* (the Hainan Tall); PDK30: *Phoenix dactylifera* (PDK30); DPV01: *Phoenix dactylifera* (DPV01); EG: *Elaeis guineensis* (African oil palm E5 build); The gene of *Elaeis oleifera* (American oil palm, O8-build) was missing, not attained from the public database;

Table 7 Statistical analysis of gene families of different species

Species	Genes number	Genes in families	Unclustered genes	Family number	Unique families	Average genes per family
C. nucifera	28,039	22,376	5,663	14,411	282	1.55
E. guineensis	30,430	22,021	8,409	13,415	262	1.64
P. dactylifera	24,908	22,193	2,715	14,074	112	1.58
S. bicolor	27,159	22,016	5,143	12,992	916	1.69
P. persica	27,792	24,276	3,516	14,443	497	1.68
S. tuberosum	34,879	28,288	6,591	13,206	1,119	2.14
G. max	42,859	38,104	4,755	14,589	1,145	2.61
A. thaliana	26,637	22,990	3,647	13,292	674	1.73
T. cacao	28,624	23,776	4,848	14,928	625	1.59
V. vinifera	25,329	19,122	6,207	13,309	599	1.44
M. acuminata	36,538	24,354	12,184	13,089	620	1.86

Figure legends

Figure 1 Morphological characteristic of coconut tree (A), spica (B), female flower (C), Male flower (D), coconut nut (E), coconut nut without skin (F), and vertical section of coconut nut (G).

Figure 2 Kmer analysis of the coconut genome.

Figure 3 The protocol for integrating GLEAN and transcriptome data.

Figure 4 Groups of orthologues shared among the angiosperms *Cocos nucifera* (Coconut), *Elaeis guineensis* (Oil palm), *Phoenix dactylifera* (Date palm), *Sorghum bicolor* (Sorghum), *Musa acuminate* (Banana) and *Arabidopsis thaliana* (Arabidopsis). Venn diagram generated by http://www.interactivenn.net/.

Figure 5. Estimation of divergence time. The blue numbers on the nodes are the divergence time from present (million years ago, Mya), the red nodes indicated the previously published calibration times.

Figure 6. Phylogenetic tree of antiporter genes from *C. nucifera* and *Arabidopsis thaliana*. Every cluster is indicated with a different colored arc line arc. The potential function of every cluster is indicated with the function groups found in *Arabidopsis thaliana*. Colored stars indicate antiporter genes of *C. nucifera*.

Figure 7. Phylogenetic tree of ion channel genes from *C. nucifera* and *Arabidopsis thaliana*. Every cluster was indicated with different colored arc line arc. The potential function of every cluster was indicated with the function groups found in *Arabidopsis thaliana*. Colored stars indicate ion channel genes of *C. nucifera*.

Additional files

Additional file 1 Identification and characterization of antiporter genes in the genome of Cocos nucifera

Additional file 2 Identification and characterization of ion channel genes in the genome of Cocos nucifera

 Click here to download Figure Figure 2.tif

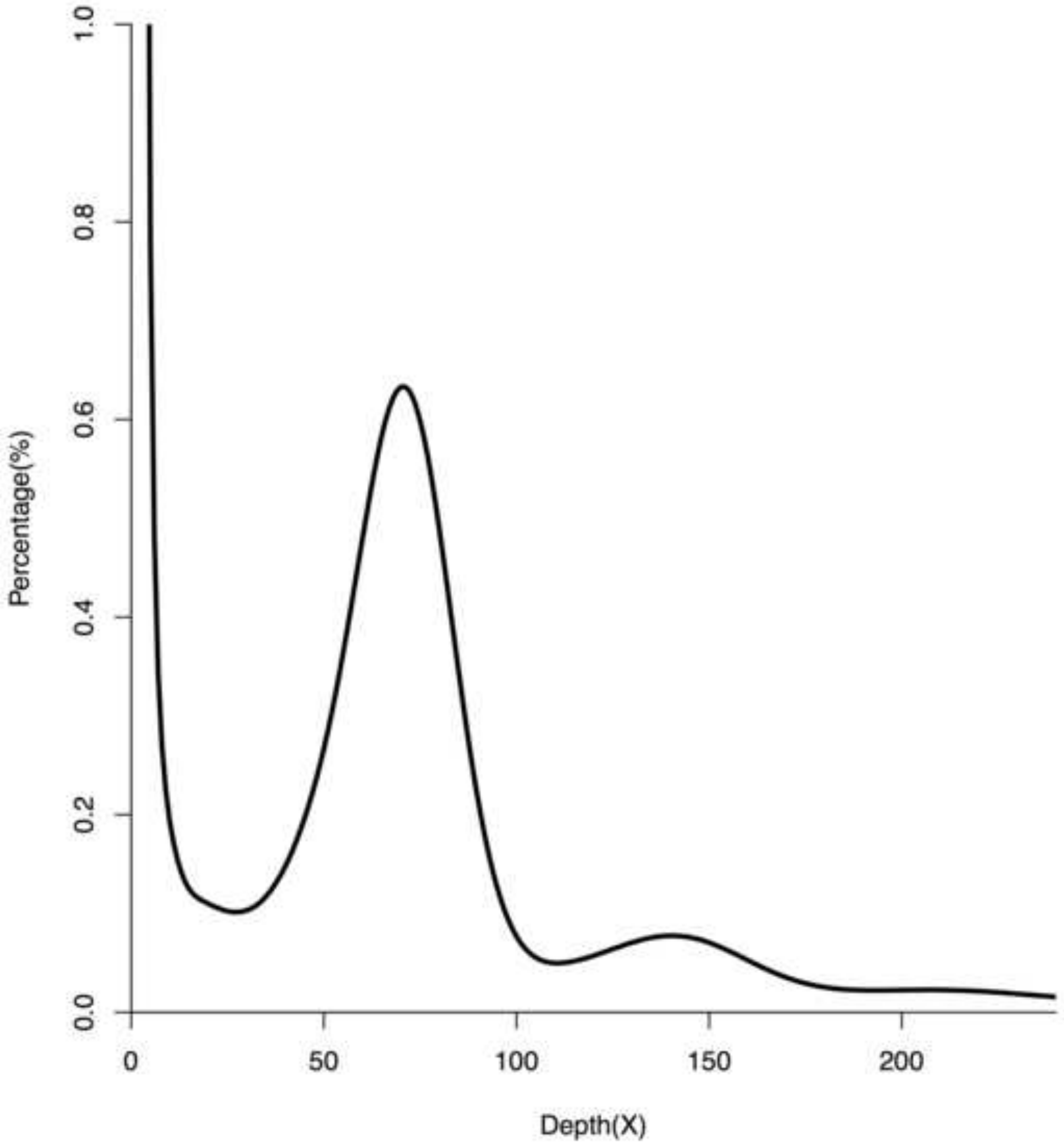

Figure

Click here to download Figure Figure 3-2.tif

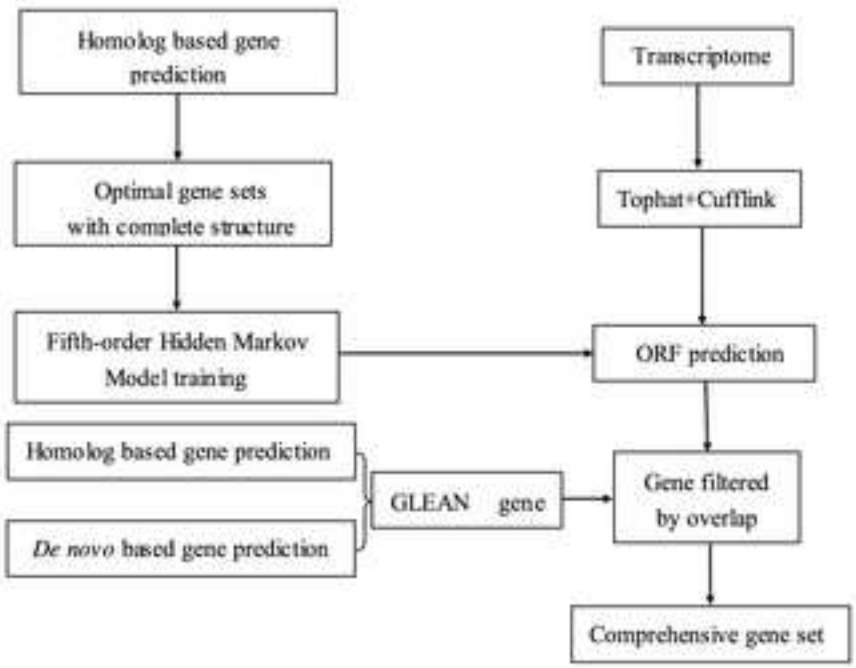

Figure

Click here to download Figure Figure 4-2.jpg

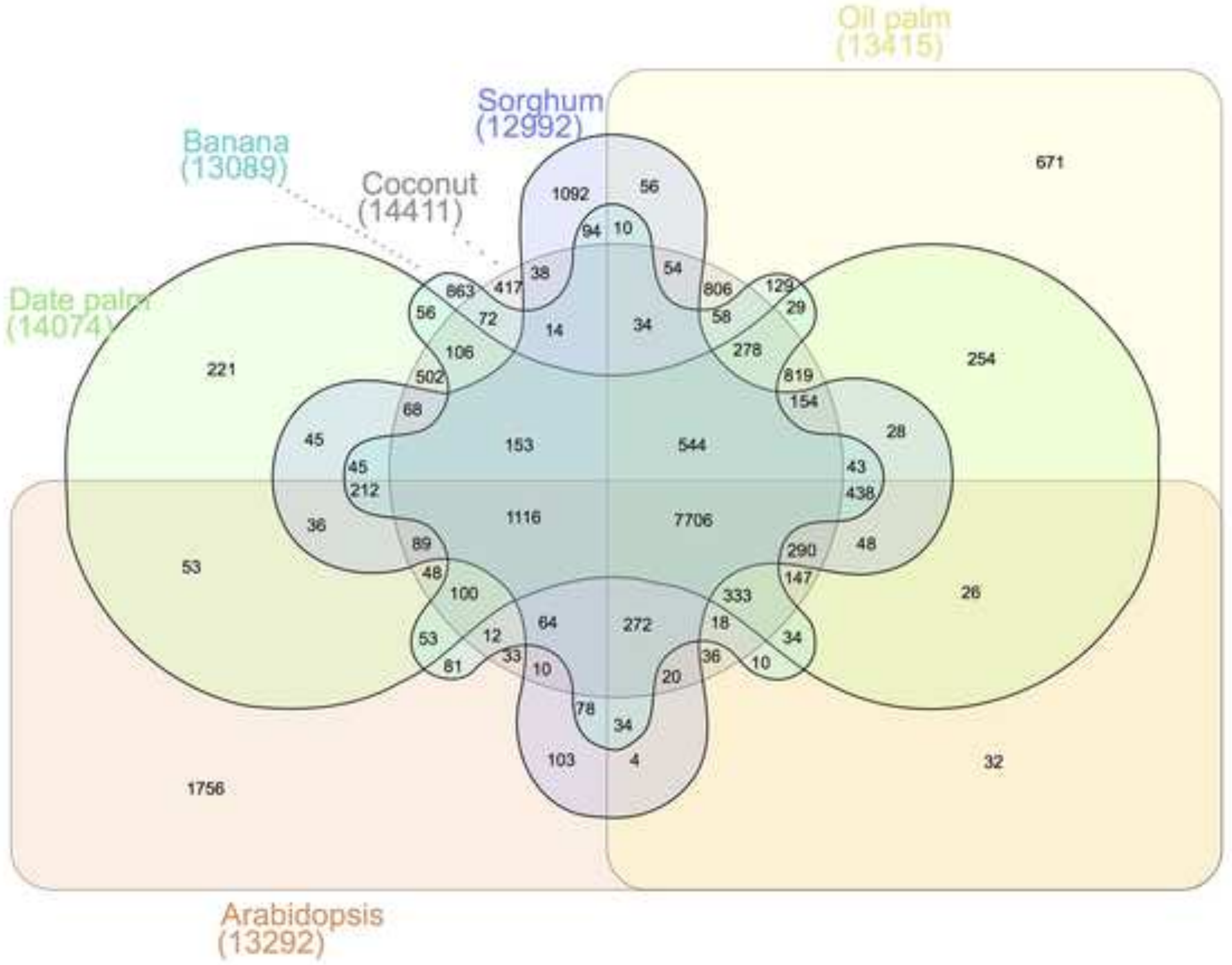

Figure

Click here to download Figure Figure 5-2.jpg

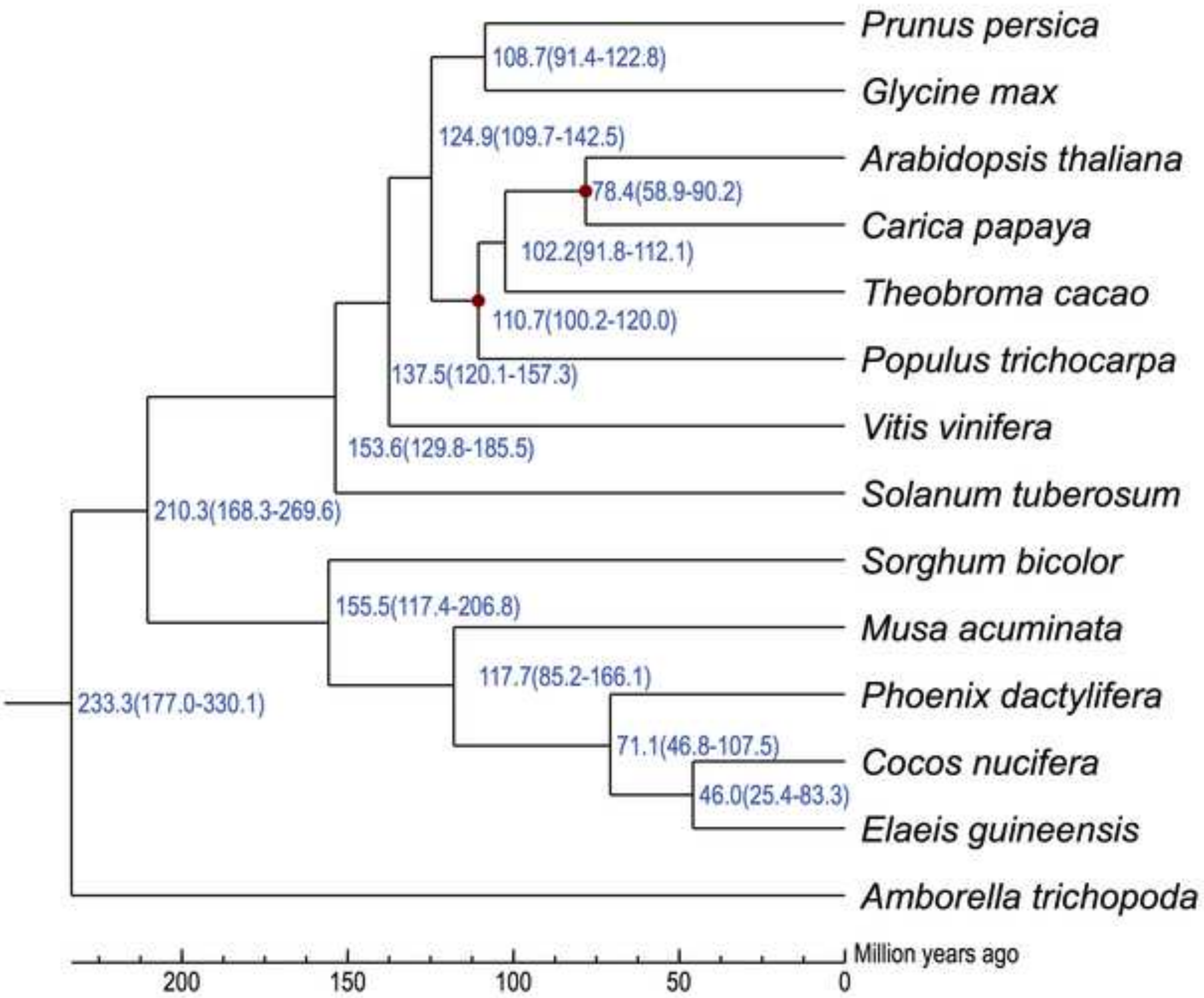

Figure

Click here to download Figure Figure 6.tif

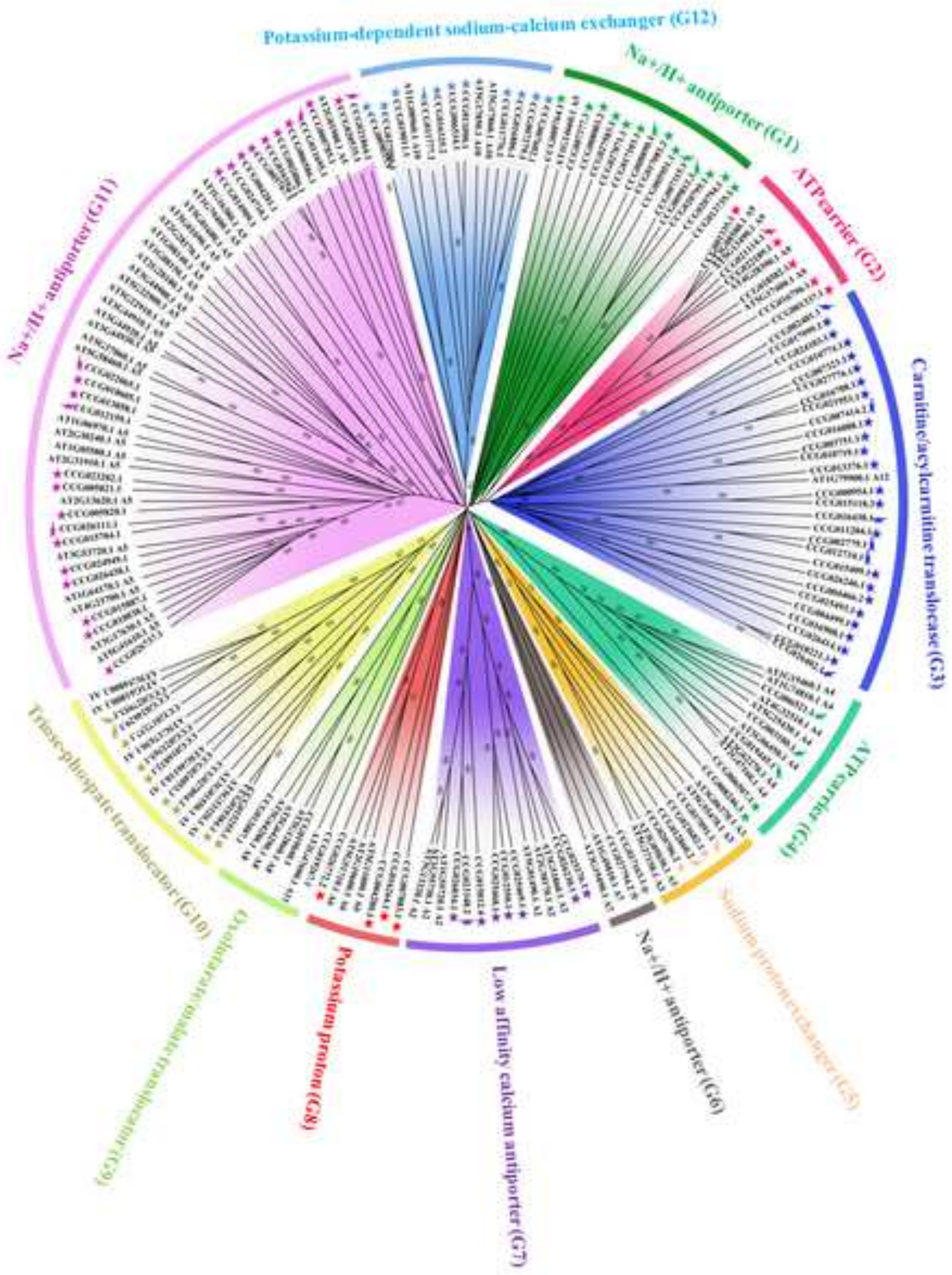

Figure

Click here to download Figure Figure 7.tif

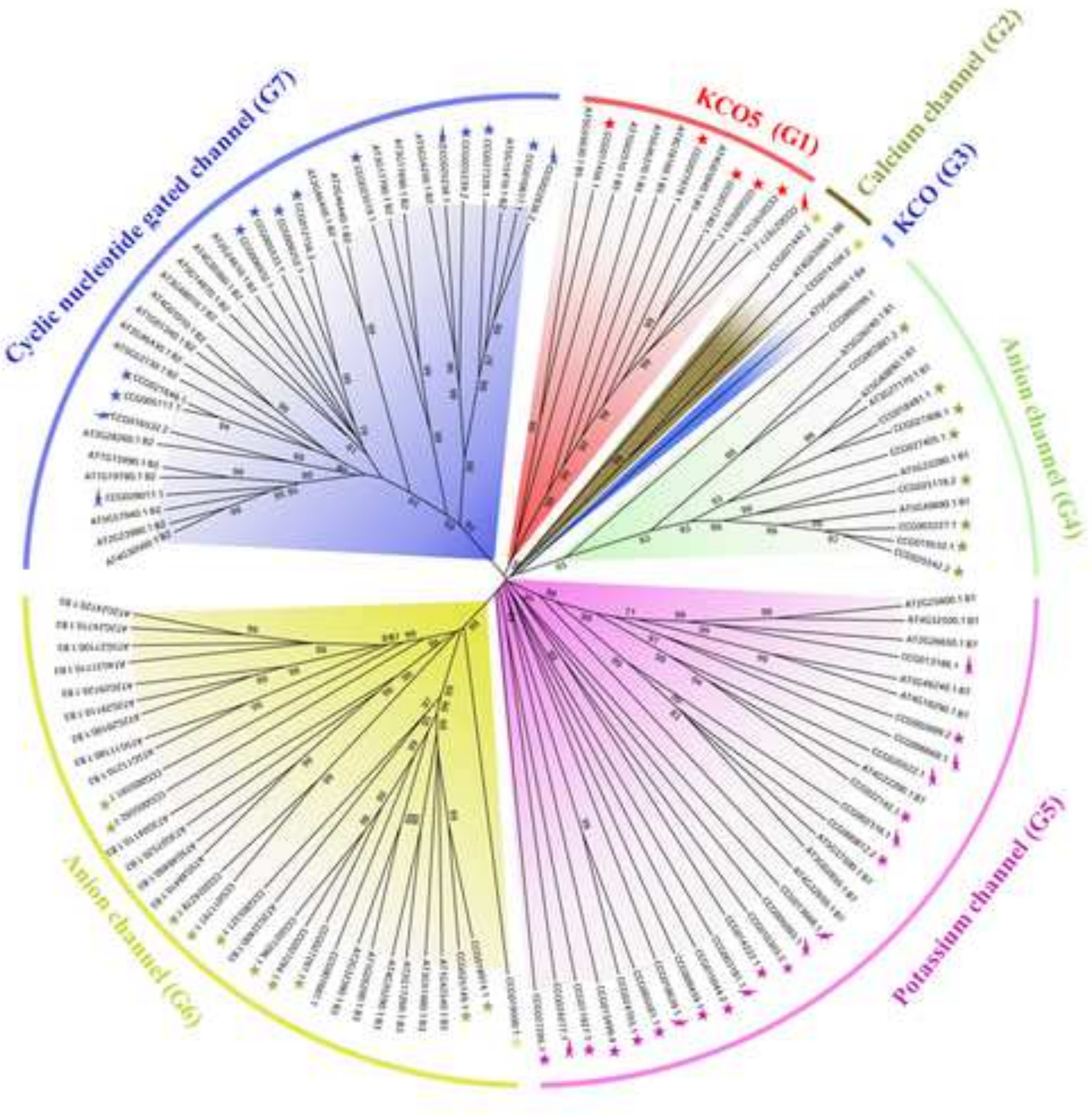

Figure

Click here to download Figure Figure 1.tif

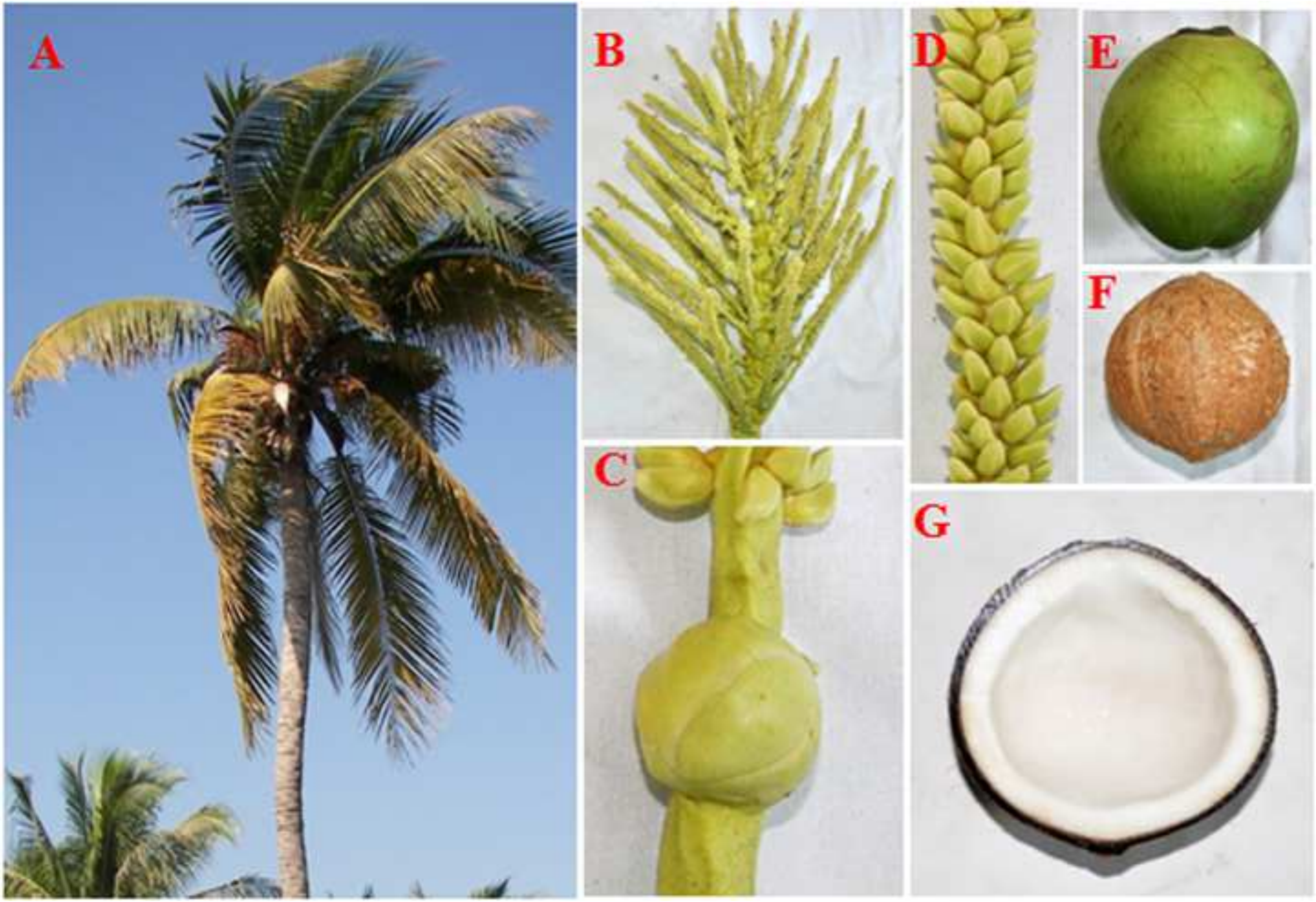

Click here to access/download
Supplementary Material
Additional file 1.xlsx

Click here to access/download
Supplementary Material
Additional file 2.xlsx
