# Supplementary material for: The genome draft of coconut (Cocos nucifera)
Source: Gigascience. 2017 Oct 5;6(11):1–11. doi: 10.1093/gigascience/gix095 (PMC5714197; doi:10.1093/gigascience/gix095)
Supplement: Response-to-Reviewer-Comments_Original-Submission.pdf [file gix095_response-to-reviewer-comments_original-submission.pdf]

Author's Response To Reviewer Comments

Response to editor and reviewers

Dear editor and reviewers

Thank you very much for your crucial comments for our manuscript entitled “The genome draft of the Coconut (Cocos nucifera)" (GIGA-D-17-00038). We have made a thorough revision to the ms based on all of comments from editor and reviewers. Each comments raised by the reviewers had been carefully answered in the response sheet. We hope the revised version can meet the requirement of “GigaScience”

Sincerely yours,

Yaodong Yang

PHONE NUMBER: 0086-898-63330602
FAX NUMBER: 0086-898-63330673
EMAIL: yyang@catas.cn
POSTAL ADDRESS:
Coconuts Research Institute, Chinese Academy of Tropical Agricultural Sciences,
496 Wenqing Av., Wenchang, Hainan 571339, P.R.China

Response to editor and reviewers,

Reviewer 1

1. Line 40: in 93 countries -> the introduction (line 70) say 89 countries

>>>Response: Thank you for your suggestion. We have re-checked the document reported by Batugal et al., 2005. The corresponding revision has been done in the Introduction part of the revised manuscript.

2. 11 million ha ->the introduction (line 72) says 12 million ha

>>>Response: Thank you for your suggestion; we have re-checked the plant area of coconut in the website of Food and Agriculture Organization of the United Nations (http://www.fao.org/faostat/en/). The corresponding revision has been done in the Abstract part of the revised manuscript.

3. Hinders progress in genetic breeding. Do you mean ‘marker assisted breeding’ or ‘genomic assisted breeding’?

>>>Response: Thank you for your suggestion; we meant to say ‘conventional breeding’. Revisions have been made in the Abstract part of the revised manuscript to make our opinions clearer.

4. Genetic improvement is slow. Do you mean trait improvement with marker or genetic assisted

>>>Response: We meant to say the improvement made by ‘conventional breeding’ is slow. The corresponding revision has been done in the revised manuscript.

5. Line 48: The coverage does not add up. 714.67 Gb on a 2.42 Gb genome is 295× coverage. In any case, only the coverage of the cleaned reads should be shown (177×)`

>>>Response: Thank you for your suggestion; in revised manuscript, only the cleaned reads were used for the coverage depth analysis and the coverage is173.32× read depth.

6. Line54: Do you mean 41,166 genes

>>>Response: Thank you for your suggestion; we have re-checked the annotated gene number for datepalm based on the document reported by AI-Mssallem et al., 2013 and 41 660 genes were annotated. The corresponding revisions have been made in the Abstract part of revised manuscript.

7. Line60: space missing between facilitating and future

>>>Response: Thank you for your suggestion, a space has been added between facilitating and future.

8. Line 61: should be ‘molecular assisted breeding’

>>>Response: Thank you for your suggestion, corresponding revisions have been done in the Abstract part of revised version.

9. Line 78: ‘…wide range to environment…’ -> unclear, should be explained. Also ‘environment’

>>>Response: Some sentences have been added to the revised manuscript for explaining ‘…wide range to environment…’ in Line 240– Line 242|Page 3.

10. Line78: ‘…especially for high tolerance to high salt density.’ , please clarify

>>>Response: Coconut palm can disseminate through ocean currents: floating nuts sprout and grow naturally upon washing up on beaches. The ability to adapt to a high salt environment is closely related to this dissemination feature and to these natural growth conditions. Corresponding revision has been done in Line 243– Line 244|Page 3 of revised manuscript.

11. Line 80: ‘…making it possible to understand its adaptation to high salinity.’ You do not investigate this, you should change the statement to something milder such as: ‘This study forms the basis for future research investigating the coconuts tolerance to salt stress’

>>>Response: Thank you for your suggestion, We also present the genome sequence of HAT coconut and added an analysis of the antiporter and ion channel gene families, relevant to salinity tolerance, into the revised version. Corresponding revision had been added into in Line 237–Line 238|Page 3.

12. Line 82: provide references. The way this sentence reads at the moment, make it seem like you are also reporting those genome sequence.

>>>Response: The corresponding references have been added into Line 423|Page 4 of revised manuscript.

13. Line 92: space between 'Illumina', 'Hiseq2000' and 'sequencer'

>>>Response: Two spaces had been added into between Illumina, Hiseq2000 and sequencer in Line 436|Page 4 of revised manuscript.

14. Line129: The data shows that you have higher coverage and a longer N50, it does not show that the assembly is of better quality.

>>>Response: Thank you for your suggestion, the sentence has been replace by other sentence: "The comparative results of the BUSCO estimation in coconut and in the four other palm genome sequences indicates that the smallest fraction of missing genes as predicted by BUSCO was found in the coconut genome assmebly", in Line 724 – Line 726|Page 6 of revised version.

15. Line 131: 'tissues', not 'issues'

>>>Response: Thank you for your suggestion, corresponding revisions has been done in the revised version.

16. Line134: table 4 and 5 are mixed up

>>>Response: We repeatedly checked Table 4 and 5. Corresponding revisions has been done in revised manuscript.

17. Line 165: BLAST not BLSAT

>>>Response: Thank you for your suggestion, 'BLSAT' had been modified in revised manuscript.

18. Line 175 (and others): keep a space between numbers and units, consistently.

>>>Response: we re-checked all numbers and units throughout the manuscript. All needed spaces have been added between numbers and units.

19. Line195: Change start of sentence (e.g. 'After the above described steps…')

>>> Response: Thank you for your suggestion, corresponding revision has been done in Line 970|Page 8 of the revised manuscript.

20. Line 196: should read: ‘than the predicted gene markers..’

>>>Response: Thank you for your suggestion, corresponding revision has been done in Line 971 | Page 8 of revised version.

21. Line203: space between ‘by’ and ‘sequence’

>>>Response: Thank you for your suggestion, a space had been added between by and sequence

22. Line211: after ref 38, just one dot

>>>Response: Thank you for your suggestion, the ref 38 and dot has been deleted in revised version.

23. Line 219: remove space between ‘mapping’ and ‘,’

>>>Response: Thank you for your suggestion, the space has been deleted between ‘mapping’ and ‘,’.

24. References: need a lot of editing to uniform

>>>Response: All references of the manuscript have been reviewed and edited based on the author guideline of “Gigascience” in the revised manuscript.

25. Tables: Headers are unclear and many abbreviations within tables are not explained

>>>Response: Thank you for your suggestion, revisions have been done for the table headers. Meanwhile, the abbreviations have been explained and replaced with corresponding full name.

26. What is the difference between Table 4 and Table 7? Both show BUSCO assessments of palm species. Clarify both in tables and in the text.

>>>Response: Thank you for your suggestion, Table 7 has been changed into Table 6 in the revised version. Table 4 referred to the comparative analysis of the assembled genome sequences for four palm species using BUSCO software, while Table 6 referred to the comparative analysis of the predicted gene from the four palm species using BUSCO software. Revisions have been done to make Table 4 and Table 6 legends more clearly in “Table” part of revised version.

27. Figure legend: Figure 1 does not contain any morphological characteristics; they are photographs of coconut plants.

>>>Response: Figure 1 had been substantially revised in the revised version.

Reviewer 2

1. My only major concern about the manuscript is that the written style is not ready for publication. There are many type and grammatical mistakes all over the main text, figure captions and table legends. The manuscript needs some extensive copy editing to be published.

>>>Response: Thank you for your suggestion, the manuscript has been reviewed and edited throughout the manuscript by the native experts (Annaliese Mason, Baudouin Luc and Amjad Iqbal).

Reviewer 3

1. Homologous gene families using a larger set of genomes would allow a gain-/loss analysis (check the Zostera (seagrass) genome paper Figure 1a for a recent example), some venn diagrams based on this showing how many gene are shared with close relative (e.g. Elaeis), other monocots (e.g. rice) and dicots (e.g. Arabidopsis) could also be generated based on this (e.g. orchid genome paper figure 1a). Asynteny/collinearity analyisis is usually included, often combined with a Ks analysis (see the orchid genome paper Figure 2, Zostera genome paper Figure 2).

>>>Response: Thank you for your suggestion, we added venn diagrams between different species and analyzed the divergence time between different species into Line 990| Page 8 - Line 1270 | Page 10 of the revised version. Meanwhile, we identified and characterized antiporter and ion channel gene family in Line 1271 | Page 10 – Line 1578 | Page 11 of revised manuscript.

2. No case study is included, I feel there should be at least one (though as the paper is submitted as a data note the journal might not require one). The authors are the first ones to have a glimpse at the genome of this species. I would make sense to check a few relevant gene families (coconut are clearly very different from seeds of other monocots, so seed related gene families would be likely candidates for a more in depth study)

>>>Response: Thank you for your suggestion. It is known that coconut palm can disseminate through ocean currents: floating nuts sprout and grow naturally upon washing up on beaches. The ability to adapt to a high salt environment is closely related to this dissemination feature and to these natural growth conditions. In the revised manuscript, we identified antiporter and ion channel genes in the genome of Cocos nucifera, some of which had been validated to be associated with salt stress in Arabidopsis. In the gene expansions analysis, some gene families showed significant expansion in compared to Arabidospsis, including Na+/H+ antiporter family, Carnitine/acylcarnitine translocase family, Potassium-dependent sodium antiporter, and potassium channel. The expansion of Na+/H+ antiporter family and Potassium-dependent sodium antiporter may be associated with coconut salt tolerance. The expansion of carnitine/acylcarnitine translocase family may be associated with the accumulation of fatty acid in coconut pulp. At last, the expansion of potassium channel may be associated with the accumulation of potassium ion in coconut water. Corresponding revision had been added into Line Line 1271 | Page 10 – Line 1578 | Page 11 of revised manuscript.

3. For non-bioinformaticians a supplemental website which offers a BLAST interface would certainly be welcome.

>>>Response: we have uploaded coconut genome raw data into Sequence Read Archive (SRA) of the National Center for Biotechnology Information. The assembled and annotated data were uploaded into GigaDB database. Meanwhile, the assembled and annotated data have been uploaded into pirate website for blast analysis and genome browse. However, currently, this website is not available for all people. The website will be available after further website improvement and paper publication

4. Line 128 -129: The N50 by itself is not a direct measure for the quality of the assembly. Avoid over-interpretation.

>>>Response: Thank you for your suggestion, the sentence has been replace by other sentence: “The comparative results of the BUSCO estimation in coconut and in the four other palm genome sequences indicates that the smallest fraction of missing genes as predicted by BUSCO was found in the coconut genome assmebly”, in Line 724 – Line 726|Page 6 of revised version.

5. Line 54 and abstract: (DVP01, 4166) -> the number of genes for the date palm genome is incorrect. In table 2 the authors report 41, 660 !

>>>Response: Thank you for your suggestion; we have re-checked the annotated gene number for datepalm based on the document reported by AI-Mssallem et al., 2013 and 41 660 genes were annotated. The corresponding revisions have been made in the Abstract part of revised manuscript.

6. Line 60: facilitating future: missing space Line 78-79

>>>Response: Thank you for your suggestion, a space has been added into between facilitating and future.

7. Line 78-79: For high tolerant to high salt density: revise grammar

>>>Response: Thank you for your suggestion, revisions have been done in Line 240– Line 242 | Page 3 of revised manuscript

8. Line 80: …present Hainan Tall… -> …present the Hainan Tall

>>>Response: Thank you for your suggestion, the sentence has been rewrite and the usage of the phrase “Hainan Tall” has been carefully checked throughout the revised manuscript.

9. Line 82: …about genome

>>>Response: Revisions have been done in revised manuscript.

10. Line 88 (and other places): …pair end… -> …paired end…

>>>Response: Thank you for your suggestion, all ‘pair end’ has been modified into ‘paired end’ throughout the revised manuscript.

11. Line 96: …removed by using … -> …removed using…

>>>Response: Thank you for your suggestion, corresponding revision had been done in revised manuscript

12. Line 116: …SOAPdenovo2 map… -> …SOAPdenovo2 maps…

>>>Response: Thank you for your suggestion, corresponding revision had been done in Line 572 | Page 5 of revised manuscript.

13. Line 132: ...reported in previous Fan’s research …: incorrect grammar should be revised (as previously reported by Fan et al…).

>>>Response: Thank you for your suggestion, corresponding revision had been done in Line 598 | Page 5 in revised manuscript

14. Line 181: Previous Fan’s research: revise grammar

>>>Response: ‘Previous Fan’s research’ had been modified into ‘as previously reported by Fan et al.’ in Line 874 | Page 7 of revised manuscript

15. Line 192: … a diagrammic pipeline is showed…: revise grammar

>>>Response: Thank you for your suggestion, corresponding revisions had been done in Line 968|Page 8 revised version.

16. Line 199: …completely… -> complete

>>>Response: Thank you for your suggestion, corresponding revision has been done in Line 973 | Page 8 of revised manuscript.

17. Line 203 -204: In sequence similarity step: revise

>>>Response: ‘In sequence similarity step’ has been modified into ‘Firstly’ in Line 979|Page 8 of the revised manuscript.

18. Line 232-233: Font is suddenly somewhat bigger

>>>Response: Thank you for your suggestion, corresponding revision have been done in “Funding” part of revised manuscript.
