# Supplementary material for: The genome draft of coconut (Cocos nucifera)
Source: Gigascience. 2017 Oct 5;6(11):1–11. doi: 10.1093/gigascience/gix095 (PMC5714197; doi:10.1093/gigascience/gix095)
Supplement: Response-to-Reviewer-Comments_Revision-1.pdf [file gix095_response-to-reviewer-comments_revision-1.pdf]

Author's Response To Reviewer Comments

Response to editor and reviewers

Dear editor

Thank you very much for your crucial comments for our manuscript entitled “The genome draft of the coconut (Cocos nucifera)” (GIGA-D-17-00038). We have made some revisions to the ms based on your comments. We removed revision mode and upload the clean manuscript without any tracking of changes. Meanwhile, we cited the reference database “Xiao Y, Xu P, Fan H, Baudouin L, Xia W, Bocs S et al. Supporting data for The genome draft of coconut (Cocos nucifera). Gigascience Database. 2017” in the revised manuscript. We hope the revised version can meet the requirement of “GigaScience”.

Sincerely yours,

Yaodong Yang

PHONE NUMBER: 0086-898-63330602
FAX NUMBER: 0086-898-63330673
EMAIL: yyang@catas.cn
POSTAL ADDRESS:
Coconuts Research Institute, Chinese Academy of Tropical Agricultural Sciences,
496 Wenqing Av., Wenchang, Hainan 571339, P.R.China
