# Supplementary material for: The genome draft of coconut (Cocos nucifera)
Source: Gigascience. 2017 Oct 5;6(11):1–11. doi: 10.1093/gigascience/gix095 (PMC5714197; doi:10.1093/gigascience/gix095)
Supplement: Reviewer-1-Report-(Original-Submission).pdf [file gix095_reviewer-1-report-(original-submission).pdf]

Reviewer Report

Title: The genome draft of coconut (Cocos nucifera)

Version: Original Submission **Date:** 4/12/2017

Reviewer name: Carsten Kulheim

Reviewer Comments to Author:

The manuscript titled 'The genome draft of the Coconut (Cocos nucifera)' describes the sequencing, assembly and annotation of the coconut genome. While there are several indicators that the work presented here is well done, the quality of the written work is so poor that it is made difficult to read and assess. The manuscript is full of typographical errors, the formating is poor and it appears that many small mistakes are present throughout the manuscript that could have been avoided by a careful final evaluation. That said, I believe that the work can be made publishable with some editing and care. Since the authors mention adaptation to salinity, a few results regarding GO or KEGG annotations to this topic would make the study more exciting.Some examples are shown below:line 40: in 93 countries -> the introduction (line 70) says 89 countriesline 41: 11 million ha -> the introduction (line 72) says 12 million haline 44: hinders progress in genetic breeding. Do you mean 'marker assisted breeding' or 'genomic assisted breeding'?line 45: genetic improvement is slow. Do you mean trait improvement with marker or genetic assisted breeding?line 48: The coverage does not add up. 714.67 Gb on a 2.42 Gb genome is 295 x coverage. In any case, only the coverage of the cleaned reads should be shown (177 x)line 54: Do you mean 41,166 genes?line 60: space missing between facilitating and futureline 61: should be 'molecular assisted breeding'line 78: '... wide range to environment...' -> unclear, should be explained. Also 'environments'line 78: '... especially for high tolerant to high salt density.' please clarifyline 80: '... making it possible to understand its adaptation to to high salinity.' You do not investigate this, you should change the statement to something milder such as: 'This study forms the basis for future research investigating the coconuts tolerance to salt stress.'line 82: provide references. The way this sentence reads at the moment, makes it seem like you are also reporting those genome sequences.line 92: space between 'Illumina', "HiSeq2000' and 'sequencer'line 129: The data shows that you have higher coverage and a longer N50, it does not show that the assembly is of better quality.line 131: 'tissues', not 'issues'line 134: tables 4 and 5 are mixed upline 165: BLAST not BLSATline 175 (and others): keep a space between numbers and units, consistently.line 195: Change start of sentence (e.g. 'After the above described steps...')line 196: should read: 'than the predicted gene numbers...'line 203: space between 'by' and 'sequence'line 211: after ref 38, just one dotline 219: remove space between 'mapping' and ','References: need a lot of editing to uniform.Tables: Headers are unclear and many abbreviations within tables are not explained.What is the difference between Tables 4 and 7? Both show BUSCO assessments of palm species. Clarify both in tables and in the text!Figure legend: Figure 1 does not contain any morphological characteristics, they are photographs of coconut plants.

Level of Interest

Please indicate how interesting you found the manuscript: An article whose findings are important to those with closely related research interests

Quality of Written English

Please indicate the quality of language in the manuscript: Not suitable for publication unless extensively edited

Declaration of Competing Interests

Please complete a declaration of competing interests, considering the following questions:

- Have you in the past five years received reimbursements, fees, funding, or salary from an organisation that may in any way gain or lose financially from the publication of this manuscript, either now or in the future?
- Do you hold any stocks or shares in an organisation that may in any way gain or lose financially from the publication of this manuscript, either now or in the future?
- Do you hold or are you currently applying for any patents relating to the content of the manuscript?
- Have you received reimbursements, fees, funding, or salary from an organization that holds or has applied for patents relating to the content of the manuscript?
- Do you have any other financial competing interests?
- Do you have any non-financial competing interests in relation to this paper?

If you can answer no to all of the above, write 'I declare that I have no competing interests' below. If your reply is yes to any, please give details below.

I declare that I have no competing interests

I agree to the open peer review policy of the journal. I understand that my name will be included on my report to the authors and, if the manuscript is accepted for publication, my named report including any attachments I upload will be posted on the website along with the authors' responses. I agree for my report to be made available under an Open Access Creative Commons CC-BY license (http://creativecommons.org/licenses/by/4.0/). I understand that any comments which I do not wish to be included in my named report can be included as confidential comments to the editors, which will not be published.

I agree to the open peer review policy of the journal
