# Supplementary material for: The genome draft of coconut (Cocos nucifera)
Source: Gigascience. 2017 Oct 5;6(11):1–11. doi: 10.1093/gigascience/gix095 (PMC5714197; doi:10.1093/gigascience/gix095)
Supplement: Reviewer-2-Report-(Original-Submission).pdf [file gix095_reviewer-2-report-(original-submission).pdf]

Reviewer Report

Title: The genome draft of coconut (Cocos nucifera)

Version: Original Submission **Date:** 4/19/2017

Reviewer name: David Chagné

Reviewer Comments to Author:

The manuscript by Xiao and colleagues presents a draft genome assembly of coconut. To my opinion the research is sound and the analysis of the data is mostly correct. It is a pity the authors did not attempt to anchor their scaffolds onto a linkage map or use modern assembly methods to achieve pseudo-chromosome assembly. Nevertheless, I believe this fragmented genome will be a useful starting point for people working in coconut genetics.My only major concern about the manuscript is that the written style is not ready for publication. There are many typos and grammatical mistakes all over the main text, figure captions and table legends. The manuscript needs some extensive copy editing to be publishable.

Level of Interest

Please indicate how interesting you found the manuscript: An article whose findings are important to those with closely related research interests

Quality of Written English

Please indicate the quality of language in the manuscript: Not suitable for publication unless extensively edited

Declaration of Competing Interests

Please complete a declaration of competing interests, considering the following questions:

- Have you in the past five years received reimbursements, fees, funding, or salary from an organisation that may in any way gain or lose financially from the publication of this manuscript, either now or in the future?
- Do you hold any stocks or shares in an organisation that may in any way gain or lose financially from the publication of this manuscript, either now or in the future?
- Do you hold or are you currently applying for any patents relating to the content of the manuscript?
- Have you received reimbursements, fees, funding, or salary from an organization that holds or has applied for patents relating to the content of the manuscript?
- Do you have any other financial competing interests?
- Do you have any non-financial competing interests in relation to this paper?

If you can answer no to all of the above, write 'I declare that I have no competing interests' below. If your reply is yes to any, please give details below.

No competing interest

I agree to the open peer review policy of the journal. I understand that my name will be included on my report to the authors and, if the manuscript is accepted for publication, my named report including any attachments I upload will be posted on the website along with the authors' responses. I agree for my report to be made available under an Open Access Creative Commons CC-BY license (http://creativecommons.org/licenses/by/4.0/). I understand that any comments which I do not wish to be included in my named report can be included as confidential comments to the editors, which will not be published.

I agree to the open peer review policy of the journal
