# Supplementary material for: The genome draft of coconut (Cocos nucifera)
Source: Gigascience. 2017 Oct 5;6(11):1–11. doi: 10.1093/gigascience/gix095 (PMC5714197; doi:10.1093/gigascience/gix095)
Supplement: Reviewer-3-Report-(Original-Submission).pdf [file gix095_reviewer-3-report-(original-submission).pdf]

Reviewer Report

Title: The genome draft of coconut (Cocos nucifera)

Version: Original Submission **Date:** 4/26/2017

Reviewer name: Sebastian Proost

Reviewer Comments to Author:

The authors present a draft of the Cocus nucifera aka Coconut genome. Apart from economic importance of the species it should also be noted that the Arecaceae provide an excellent outgroup for evolutionary studies of the Poaceae which include all major cereals. The manuscript mainly highlights the methods used to sequence the genome and assemble the reads (which has been done using current standards), but offers little more than that.MAJOR======This manuscript is little more than a claim the coconut genome has been sequenced. Some traditional analyses one would expect from a genome paper are missing. Homologous gene families using a larger set of genomes would allow a gain-/loss analysis (check the Zostera (seagrass) genome paper Figure 1a for a recent example), some venn diagrams based on this showing how many genes are shared with close relative (e.g. Elaeis), other monocots (e.g. rice) and dicots (e.g. Arabidopsis) could also be generated based on this (e.g. orchid genome paper figure 1a). A synteny/collinearity analysis is usually included, often combined with a Ks analysis (see the Orchid genome paper Figure 2, Zostera genome paper Figure 2).MINOR======No case study is included, I feel there should be at least one (though as the paper is submitted as a data note the journal might not require one). The authors are the first ones to have a glimpse at the genome of this species. I would make sense to check a few relevant gene families (coconuts are clearly very different from seeds of other monocots, so seed related gene families would be likely candidates for a more in depth study)For non-bioinformaticians a supplemental website which offers a BLAST interface would certainly be welcome.Line 128 - 129: the N50 by itself is not a direct measure for the quality of the assembly. Avoid over-interpretation.Various typos and grammatical mistakesLine 54 and abstract: (DVP01, 4,166) -> the number of genes for the date palm genome is incorrect. In table 2 the authors report 41,660 !Line 60: facilitatingfuture: missing space Line 78-79: for high tolerant to high salt density: revise grammar Line 80: …present Hainan Tall… -> …present the Hainan Tall… Line 82: …about genome… : revise grammar Line 88 (and other places) : …pair-end… -> …paired-end… Line 96: … removed by using … -> … removed using … Line 116: …SOAPdenovo2 map … -> …SOAPdenovo2 maps … Line 132: … reported in previous Fan's research … : incorrect grammar, should be revised (as previously reported by Fan et al. ..) Line 181: Previous Fan's study : revise grammar Line 192: … a diagrammic pipeline is shown …: revise grammar Line 199: … completely … -> complete Line 203 - 204: In sequence similarity step: revise Line 232-233: Font is suddenly somewhat bigger

Level of Interest

Please indicate how interesting you found the manuscript: An article whose findings are important to those with closely related research interests

Quality of Written English

Please indicate the quality of language in the manuscript: Needs some language corrections before being published

Declaration of Competing Interests

Please complete a declaration of competing interests, considering the following questions:

- Have you in the past five years received reimbursements, fees, funding, or salary from an organisation that may in any way gain or lose financially from the publication of this manuscript, either now or in the future?
- Do you hold any stocks or shares in an organisation that may in any way gain or lose financially from the publication of this manuscript, either now or in the future?
- Do you hold or are you currently applying for any patents relating to the content of the manuscript?
- Have you received reimbursements, fees, funding, or salary from an organization that holds or has applied for patents relating to the content of the manuscript?
- Do you have any other financial competing interests?
- Do you have any non-financial competing interests in relation to this paper?

If you can answer no to all of the above, write 'I declare that I have no competing interests' below. If your reply is yes to any, please give details below.

I declare that I have no competing interests

I agree to the open peer review policy of the journal. I understand that my name will be included on my report to the authors and, if the manuscript is accepted for publication, my named report including any attachments I upload will be posted on the website along with the authors' responses. I agree for my report to be made available under an Open Access Creative Commons CC-BY license (http://creativecommons.org/licenses/by/4.0/). I understand that any comments which I do not wish to be included in my named report can be included as confidential comments to the editors, which will not be published.

I agree to the open peer review policy of the journal
